# Mast-cell derived nerve growth factor drives ILC2 pro-tumoral functions in bladder cancer

**DOI:** 10.1038/s41467-026-69841-y

**Published:** 2026-02-21

**Authors:** Maryline Falquet, Hajar El Ahanidi, Alejandra Gomez-Cadena, Ziyang Su, Anthony Cornu, Tania Wyss, Burak Kizil, Robert Pick, Katayoun Falamaki, Pratyaksha Wirapati, Benedetta Fiordi, Isis Senoner, Daniela Claudia Maresca, Neil Kallal, Danaé Guedj, Mario Kreutzfeldt, Jean-Christophe Tille, Marine M. Leblond, Katarzyna Michaud, Silvia Pesce, Simona Candiani, Korneliusz Golebski, Julien Dagher, Melinda Charrier, Caroline Pressacco Brossier, Elisabeth Grobet-Jeandin, Romina Marone, Stéphanie Hugues, Lukas T. Jeker, Grégory Verdeil, Doron Merkler, Emanuela Marcenaro, Christoph Scheiermann, Mohammed Attaleb, Daniel Benamran, Petros Tsantoulis, Giuseppe Ercolano, Sara Trabanelli, Camilla Jandus

**Affiliations:** 1 Department of Pathology and Immunology, Faculty of Medicine, University of Geneva, Geneva, Switzerland; 2 Ludwig Institute for Cancer Research, Lausanne Branch, Lausanne, Switzerland; 3 Geneva Center for Inflammation Research, Geneva, Switzerland; 4 Institute of Genetics and Genomics of Geneva (iGE3), University of Geneva, Geneva, Switzerland; 5 Translational Research Centre in Onco-Hematology (CRTOH), Geneva, Switzerland; 6 Translational Data Science Facility, AGORA Cancer Research Center, SIB Swiss Institute of Bioinformatics, Lausanne, Switzerland; 7 Department of Internal Medicine Specialties, Faculty of Medicine, University of Geneva, Geneva, Switzerland; 8 Department of Pharmacy, University Federico II of Naples Italy, Naples, Italy; 9 Department of Diagnostics, Division of Clinical Pathology, University Hospital of Geneva, Geneva, Switzerland; 10 Department of Oncology, Faculty of Biology and Medicine, University of Lausanne, Lausanne, Switzerland; 11 University Centre of Legal Medicine Lausanne-Geneva, Lausanne University Hospital, University of Lausanne, Lausanne, Switzerland; 12 Department of Experimental Medicine, University of Genova, Genova, Italy; 13 RCCS Azienda Ospedaliera Metropolitana, Genova, Italy; 14 Department of Earth, Environment and Life Sciences, University of Genova, Genova, Italy; 15 Department of Otorhinolaryngology and Head and Neck Surgery, Amsterdam UMC, University of Amsterdam, Amsterdam, The Netherlands; 16 Institute of Pathology, Lausanne University Hospital and University of Lausanne, Lausanne, Switzerland; 17 Department of Oncology, Precision Oncology Service, University Hospital of Geneva, Geneva, Switzerland; 18 Division of Urology, Geneva University Hospitals, Geneva, Switzerland; 19 Department of Biomedicine, Basel University Hospital and University of Basel, Basel, Switzerland; 20 Transplantation Immunology & Nephrology, Basel University Hospital, Basel, Switzerland; 21 Centre National de l’Energie, des Sciences et Techniques nucléaires (CNESTEN), Rabat, Morocco; 22 Present Address: Human Islet Isolation Facility, Nuffield Department of Surgical Sciences, University of Oxford, Oxford, UK

**Keywords:** Immunosurveillance, Cancer microenvironment, Lymphocytes

## Abstract

Innate lymphoid cells type 2 (ILC2s) are key regulators of tissue homeostasis and inflammation. In cancer, ILC2s can exhibit pro-tumoral functions by increasing the myeloid derived suppressor cells (MDSC)/T-cell ratio. Nevertheless, the upstream ILC2 triggers remain poorly defined. Here, we identify nerve growth factor (NGF) as the driver of ILC2 pro-tumoral functions in patients with bladder cancer. We show that ILC2s express the NGF receptor TrkA and respond to NGF by secreting type-2 cytokines. In the tumor microenvironment, NGF-producing mast cells accumulate and activate ILC2s to induce regulatory T cells (Tregs), ultimately fostering tumor growth. In patients, NGF levels inversely correlate with survival in ILC2-rich tumors, underscoring the clinical significance of this axis. In vivo administration of a selective TrkA inhibitor improves survival in orthotopic tumor-bearing female mice and sensitizes them to immune checkpoint blockade (ICB). Overall, we identify NGF as an ILC2 activator that shapes pro-tumoral ILC2 functions. The blockade of TrkA^+^ ILC2s might represent a targetable strategy to improve survival, particularly in ICB-resistant patients.

## Abstract (web summary)

Neurotrophic factor Nerve Growth Factor (NGF) is involved in bladder physiopathology. Here the authors report that mast-cell derived NGF sustains the pro-tumoral functions of ILC2s in bladder cancer (BC), showing that selective targeting of the NGF receptor TrkA improves survival and response to immune checkpoint blockade in BC models.

## Introduction

Bladder cancer (BC) is the 10^th^ most diagnosed cancer, characterized by a poor response to conventional treatment and high relapse rate^1^. Up to 40% of patients with non-muscle invasive bladder cancer (NMIBC) fail to respond to intravesical bacillus Calmette-Guérin (BCG) therapy^2^, and relapse or progress to the muscle invasive (MIBC) or metastatic state. Despite encouraging clinical activity in multiple clinical settings, anti-PD-1/PD-L1 therapies only produce durable benefit in a minority of patients with BC^3^. There is thus an urgent need for the identification of new therapies for BC.

BC is distinguished by a predominant immunosuppressive immune cell infiltrate unable to mount an adequate response to immunotherapies^4^. The BC immunosuppressive microenvironment is sustained by different immune cells such as regulatory T cells (Tregs), tumor associated macrophages^5^ and type 2 innate lymphoid cells (ILC2s) that, via IL-13 secretion, modulate the T cell/monocytic myeloid-derived suppressor cell (M-MDSC) ratio in favor of immune evasion^6^.

ILC2s are a subset of innate lymphoid cells (ILCs) that release IL-5 and IL-13, driving a type 2 immune response^7–9^, which is involved in tissue homeostasis, infection, inflammation and cancer^10,11^. The ILC family represents the innate counterpart of helper CD4 T cells, sharing similar transcription factors and specialized cytokine secretion^10^. As tissue resident cells, ILCs respond quickly to various cues and are easily imprinted by different tissue-specific signals that can drive their tissue heterogeneity^7,12^. However, ILCs are also present in the periphery, where they contribute to systemic immunity^13^.

ILC2s have divergent roles in tumor immunity, displaying either pro-^14–20^ or anti-tumoral^21–25^ features, depending on which factors are sustaining their activation, on which subpopulation of ILC2 is present and on the cancer type. Besides the typical ILC2 activators (e.g., IL-33, prostaglandin D_2_ and IL-25^9,10,26^), distinct neuronal derived molecules^27–29^, such as neuropeptides, neurotransmitters and neurotrophic factors, can modulate ILC2 functions in different inflammatory contexts, such as allergy and infections^30^. However, whether neuronal-derived factors can regulate ILC2 functions in tumor immunity is unknown.

Besides its typical role as inducer of neuronal growth, the neurotrophic factor Nerve Growth Factor (NGF) is expressed in normal bladder, in *human*^31^ and *mice*^32^. NGF has been shown to regulate bladder function and to be involved in several bladder pathological conditions, such as overactive bladder and interstitial cystitis^33^, by binding to its high affinity and low affinity receptors. In the context of cancer, neurotrophins, including NGF, and their receptors have recently gained increasing therapeutic attention as drivers of cancer neurogenesis, and for their direct effect on tumor cell growth and angiogenesis^34^. As a consequence, a considerable number of studies have evaluated the therapeutic potential of recently developed anti-Trk (neurotrophin receptors) drugs, either alone or in combination with chemotherapy, in vitro in tumor cells or in vivo in tumor *mouse* models^35^. Strong pre-clinical efficacy was reported in different tumor types (e.g., gastric cancer, prostate cancer), with favorable safety profiles. However, the mode of action of these compounds on the tumor immune infiltrate is very limited^36^.

In this study, we identify NGF as a trigger of ILC2 activation in the context of BC, both in an orthotopic *mouse* model and in patients. We find that ILC2s specifically express the NGF high affinity receptor TrkA and respond to NGF stimulation by secreting IL-5 and IL-13. Moreover, we identify mast cells as source of NGF in the bladder and Tregs as key players in sustaining the pro-tumoral role of ILC2s. Further, we show that blockade of NGF-TrkA signaling reduces ILC2 effector functions and results in survival advantage in an orthotopic *mouse* model of bladder cancer, as monotherapy and in synergy with immune checkpoint blockade (ICB). Our work dissects the NGF-TrkA-ILC2s axis in BC development and progression and demonstrates TrkA blockade as a potential therapeutic strategy for patients with BC, particularly in ICB-resistant patients. Lastly, the association of NGF levels with reduced patients’ survival across ILC2-rich tumor types underscores the clinical significance of this axis in other *human* cancer types.

## Results

### Increased NGF and ILC2 levels in *human* bladder tumorigenesis

**Fig. 1:**
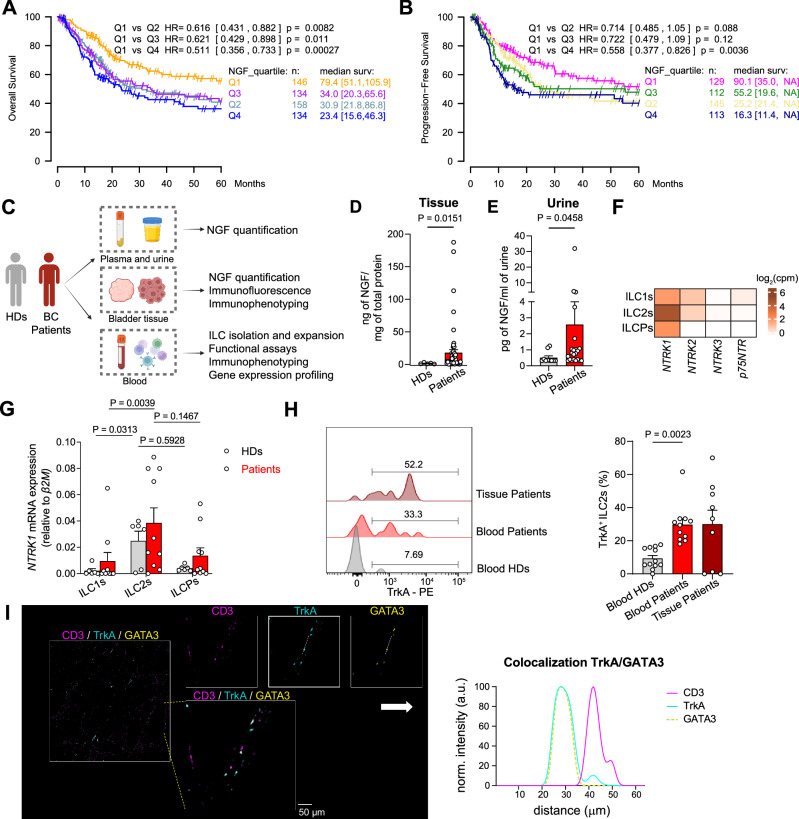
NGF and ILC2s are associated with *human* bladder tumorigenesis. **A** Overall survival (OS) analysis performed on the impact of *NGF* expression in patients with bladder cancer (BC), based on TCGA BLCA, GSE31684 and GSE48075. **B** Progression-Free Survival (PFS) analysis performed on the impact of *NGF* expression in BC patients, based on TCGA BLCA and GSE31684. **C** Pipeline used for the *human*-centered analyses. **D** NGF quantification in bladder tissue lysates from healthy cadaveric donors (HDs, *n* = 5) and BC patients (*n* = 59). **E** NGF quantification in the urine of healthy donors (HDs, *n* = 10) and BC patients (*n* = 22). **F** Heatmap generated using publicly available RNAseq data (accession number E-MTAB-8494) from healthy *human* peripheral ILCs representing the neurotrophic receptors’ expression. The value of each gene is the average from 3 donors. **G** Relative expression of *NTRK1* in ex vivo sorted ILC subsets from the peripheral blood of HDs (*n* = 6) and BC patients (*n* = 10). **H** Representative histograms (left) and quantification (right) of TrkA expression by ex vivo ILC2s from HDs (*n* = 12) and BC patients’ blood (*n* = 11) and tumor tissue (*n* = 9). **I** Representative confocal images of 10 independent tissue sections stained for ILC2s (GATA3, yellow), TrkA (blue), and T cells (CD3, magenta). A dashed box marks a region shown at higher magnification. TrkA-GATA3 colocalization is demonstrated by overlapping intensity profiles (TrkA: blue line; GATA3: dotted yellow line) along the segmented white dotted line. Scale bar, 50 µm (*n* = 10). Survival curves in **A**) and **B**) include 572 and 499 patients, respectively, categorized into NGF expression quartiles, with differences determined using two-sided Cox regression analysis. For **D**–**H**, data are represented as the mean ± SEM and pooled from two to three independent experiments on independent biological replicates. *P* values were determined by two-tailed Mann–Whitney test (**D**, **E**), by two-tailed Wilcoxon matched-pairs signed rank test (**G**) and by two-tailed Dunn’s multiple comparisons test (**H**). Panel C was “Created in BioRender. Jandus, C. (2026) https://BioRender.com/xmyyvwk”. Source data are provided as a Source Data file.

To assess the potential relevance of NGF in BC, we mined publicly available bulk RNA sequencing (RNAseq) datasets of *human* BC and assessed whether NGF expression was correlated with BC patient overall survival (OS) or progression-free survival (PFS). OS analysis was performed on a total of 572 patients, pooled from three datasets, namely TCGA BLCA^37^, GSE31684^38^ and GSE48075^39^. PFS was explored on TCGA BLCA and GSE31684 pooled together with a total of 499 patients. Expression levels of NGF were categorized into four quartiles. Patients in the highest NGF expression quartiles performed significantly worse in terms of survival, both overall and progression-free (Fig. 1A–B), arguing for a potential role of this neurotrophin in BC progression. To experimentally determine the pathophysiological role of the NGF-TrkA axis in BC, we collected specimens from patients with BC and healthy donors (HDs) (i.e., serum, urine, peripheral blood cells, tumor tissues) combined with the interrogation of bulk immune cell transcriptomic datasets and the quantification of NGF and its receptors by multiple methodologies (Fig. 1C). NGF concentration in *human* urine and bladder tissue samples was increased in patients with BC as compared to HDs (Fig. 1D–E), while no difference was observed when comparing serum samples in the two cohorts (Fig. S1A).

TrkA is the high affinity receptor for NGF^40^, and NGF signaling modulates inflammatory responses in the bladder^41^ and in other tissues. Given the role of ILC2s in BC recurrence in patients^6^ and their responsiveness to neural peptides, neurotrophins and neurotransmitters in inflammatory settings^27,42^, we reasoned that ILC2s might express the NGF receptors. By mining our previously published^43^ RNAseq dataset, as well as two other publicly available datasets^44,45^ of sorted *human* peripheral ILCs, we found that, among the neurotrophic receptors (*NTRK1*, encoding for TrkA; *p75NTR*, encoding for the NGF low-affinity receptor p75; *NTRK2*, encoding for Tropomyosin receptor kinase B, TrkB; *NTRK3*, encoding for Tropomyosin receptor kinase C, TrkC), only *NTRK1* was overexpressed by *human* ILC2s (Fig. 1F and S1B–C). To confirm this finding, we sorted the 3 main ILC subsets from the peripheral blood of HDs and BC patients and compared the neurotrophic receptors’ transcriptional levels by qPCR (Fig. S1D for the sorting strategy). We found that *NTRK1* was preferentially expressed by ILC2s both in HDs and in BC patients (Fig. 1G and S1E–F) and we confirmed its expression at protein level, with a significant increase in ILC2s in peripheral blood of BC patients, and similarly preserved also in patients’ tumor tissues (Fig. 1H). To uncover transcriptomic differences between TrkA^+^ and TrkA- ILC2s, we sorted these two subpopulations from three HDs and performed bulk RNAseq. Differential gene expression analysis revealed that 45 genes were significantly upregulated in TrkA^+^ ILC2s, while 27 genes were elevated in TrkA^-^ ILC2s. TrkA^+^ ILC2s showed markedly higher expression of *KLRC1* (encoding for NKG2A), *KLRK1* (NKG2D) and *KLRD1* (CD94), surface receptors typically enriched in NK cells, as well as *TYROBP (KARAP/DAP12)*^46^, which encodes a transmembrane signaling adapter containing an immunoreceptor tyrosine-based activation motif (ITAM) in its cytoplasmic domain. TYROBP is known to associate with the killer-cell inhibitory receptors (KIRs) and mediate activating signal transduction, suggesting that TrkA^+^ ILC2s may be functionally modulated through NK-like pathways. In contrast, TrkA^-^ ILC2s exhibited property of pro-inflammation, indicated by increased expression of *IL22* and TNF superfamily members such as *CD70* and *TNFSF9* (encoding 41BB-L) (Fig. S2). Finally, using colocalization analyses, we confirmed the preferential expression of TrkA on ILC2s (identified as CD45^+^CD3^-^GATA3^+^) in tissue sections from patients (Fig. 1I).

**Fig. 2:**
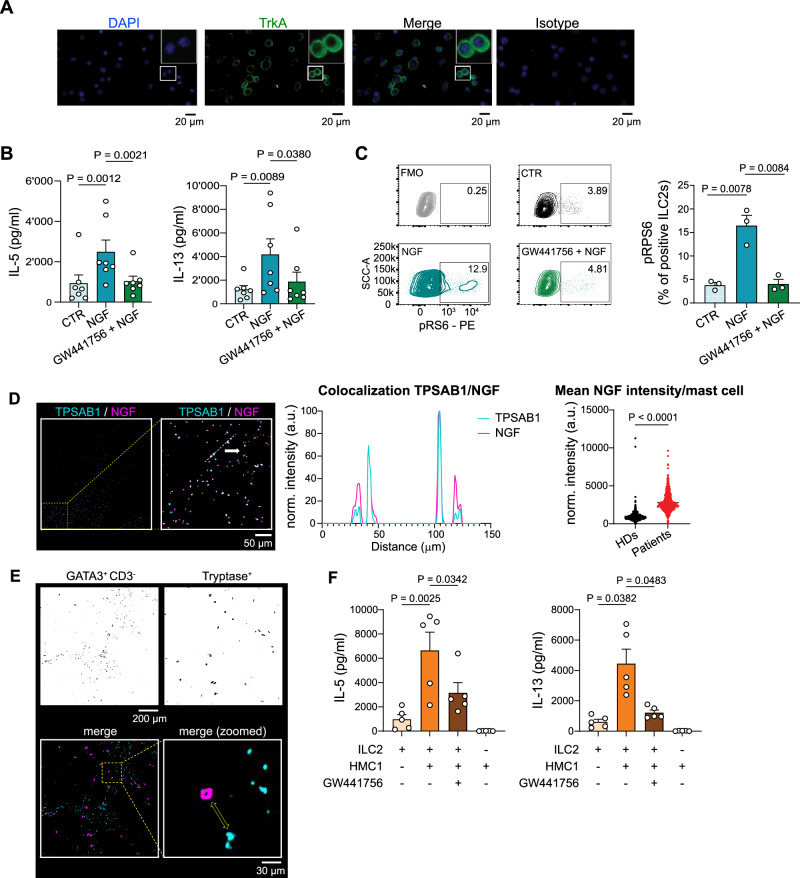
Mast-cell derived NGF drives ILC2 functions via TrkA engagement. **A** Immunofluorescence staining of TrkA on in vitro expanded ILC2s from HDs (*n* = 3). **B** IL-5 (left) and IL-13 (right) secretion by in vitro expanded ILC2s from 7 HDs unstimulated (CTR), stimulated with NGF (NGF) and pre-treated with GW441756 prior NGF stimulation (GW441756 + NGF) (*n* = 7). **C** Representative plots (left) and quantification (right) of phosphorylated S6 (pRPS6) of in vitro expanded ILC2s from 3 HDs, unstimulated (CTR), stimulated with NGF (NGF), and pre-treated with GW441756 prior NGF stimulation (GW441756 + NGF) (*n* = 3). Fluorescence minus one (FMO) control is shown as upper left plot. **D** Confocal images of tissue sections from HDs (*n* = 5) and BC patients (*n* = 5) immunostained for mast cells (TPSAB1, blue) and NGF (magenta). A dashed box marks a region shown at higher magnification. NGF-TPSAB1 colocalization is shown by overlapping intensity profiles (TPSAB1: blue; NGF: magenta) along the segmented line shown in the zoomed-in merged image. Scale bar, 50 µm. Mean NGF intensity per single mast cell (TPSAB1^+^) was quantified from confocal images after background subtraction. n_(field of view)_ = 10 (HDs)/ 11 (patients); n_(cells)_= 2752 (HDs)/ 3582 (patients). **E** Confocal images of tissue section showing ILC2s (GATA3^+^/ CD3^-^) and mast cells (Tryptase^+^). A dashed box marks a magnified region, and a yellow arrow highlights the ILC2-mast cell proximity. Scale bar, 30 µm (*n* = 6). **F** IL-5 (left) and IL-13 (right) secretion by in vitro expanded ILC2s from 5 HDs cultured alone, or co-cultured with pre-stimulated HMC1 cell line. ILC2s were pre-treated or not with GW441756 prior co-culture (*n* = 5). The pre-stimulated HMC1 cell line cultured alone is shown as right bar. Data are the mean ± SEM and pooled from two to three independent experiments on independent biological replicates. *P* values were determined by two-tailed Tukey’s multiple comparisons test (**B**, **C**), two-tailed Mann–Whitney test (**D**) and two-tailed Sidak’s multiple comparisons test (**F**). Source data are provided as a Source Data file.

The specific expression of TrkA by ILC2s was maintained after short-term in vitro expansion both at the mRNA (Fig. S3A) and protein level (Figs. 2A and S3B), validating these cells as a valuable tool to study NGF-driven functions in ILC2s in vitro. Of note, while tumor cells of different origins are known to express TrkA, a healthy *human* immortalized bladder urothelial cell line (HCV29) as well as several *human* bladder cancer cell lines showed barely detectable transcripts of neurotrophic receptors (Fig. S3C). Altogether, these results suggest a NGF-rich BC environment, where NGF might preferentially signal through TrkA^+^ ILC2s, rather than directly on tumor cells.

### Mast-cell-derived NGF drives ILC2 functions via TrkA engagement

The main function of ILC2s is the secretion of Type-2 cytokines. To define a putative role for NGF in ILC2s, we stimulated short-term expanded *human* ILC2s with recombinant *human* NGF and analyzed cytokine secretion. NGF-stimulated ILC2s secreted high levels of the Type-2 cytokines IL-5 and IL-13 (Fig. 2B). To assess the specificity of the NGF effect, we pretreated ILC2s with non-toxic concentrations of GW441756, a selective TrkA inhibitor^47^ (Fig. S3D). Upon treatment with the inhibitor, the NGF-mediated secretion of IL-5 and IL-13 was reverted to basal levels (Fig. 2B), demonstrating that NGF triggers ILC2 cytokine production via TrkA engagement. Upon NGF treatment, higher levels of IL-4 and IL-10 were observed in comparison to unstimulated cells, but not of IL-9, although these cytokines were secreted in very little amounts (Fig. S3E). NGF stimulation could also induce Type-2 cytokine production in ex vivo freshly sorted ILC2s from HDs (Fig. S3F). No difference was found in ILC2 viability or proliferation in control versus NGF-treated cells (Figs. S3G and S3H), suggesting that the increased cytokine production was not a consequence of ILC2 fitness or proliferation.

To gain insights into the mechanism of NGF stimulation in ILC2s, we characterized the NGF-TrkA-associated pathways such as the mTORC1, the mitogen-activated protein kinases (MAPK) and Akt pathways, known to be activated in NGF-stimulated tumor cells^48^. Upon NGF stimulation, we found increased phosphorylation of ribosomal protein S6 (pRPS6) (Fig. 2C and S3I). Given that previous studies have shown that ILC2s utilize both oxidative phosphorylation (OXPHOS) and glycolysis depending on their activation state^49^, we assessed whether NGF treatment affects mitochondrial fitness. However, no significant changes were observed following NGF stimulation (Fig. S3L).

To search for the source of NGF in the bladder we considered mast cells, since they have been shown to produce, store and release NGF^50^ and to infiltrate the bladder^51^. We found a positive correlation between *TPSAB1* (used as surrogate marker for mast cells) and *NGF* expression in the BC patients’ cohort of the TCGA dataset (Fig. S4A). We further confirmed increased infiltration of NGF expressing mast cells (identified by tryptase alpha-1 (TPSAB1) positivity) in bladder tissue sections from patients with cancer, compared to healthy controls (Fig. 2D). Furthermore, spatial analysis demonstrated close proximity between TrkA^+^ ILC2s and mast cells in tumor sections (Fig. 2E), arguing for an interaction between the two cell populations.

To simulate ILC2 activation by a more physiological source of NGF, rather than the recombinant one (Fig. 2B), we stimulated the *human* HMC1 mast cell line and confirmed NGF expression by immunofluorescence (Fig. S4B). Pre-activated HMC1 cells were then used to stimulate ILC2s in co-culture experiments, resulting in significant Type-2 cytokine secretion (Fig. 2F). This effect was inhibited by pre-treatment of ILC2s with the TrkA inhibitor GW441756 (Fig. 2F). Collectively, these results demonstrate that mast-cell derived NGF is a potent and fast ILC2 activator, and its downstream signaling involves, in part, mTOR activation.

### Mast cell - TrkA^+^ ILC2 axis in murine bladder cancer

**Fig. 3:**
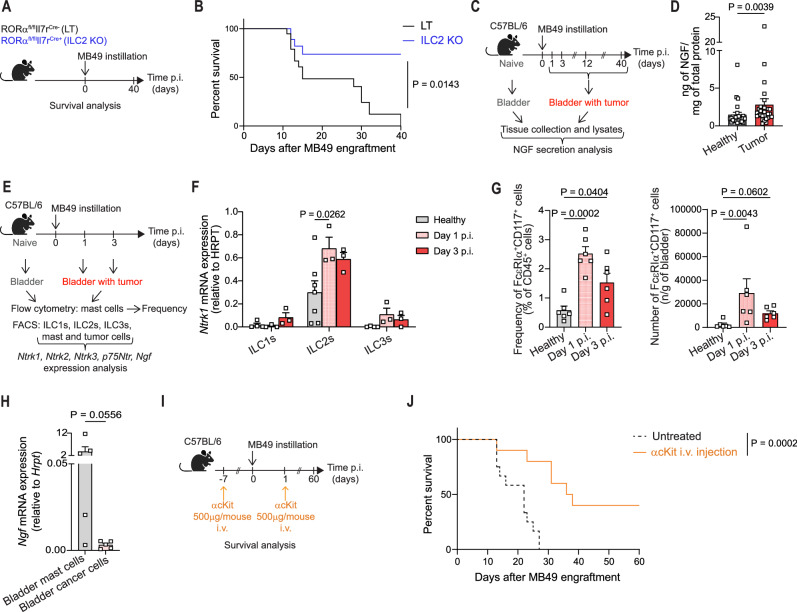
ILC2s specifically express TrkA in *mouse* bladder. **A** In vivo experimental design. **B** Survival curve of MB49 tumor-bearing *Rorα*^fl/fl^*Il7r*^Cre-^ (LT, *n* = 15) and *Rorα*^fl/fl^*Il7r*^Cre+^ (ILC2 KO, *n* = 15) *mice*. **C** Schematic representation of the in vivo experimental design for NGF secretion analysis. **D** Quantification of NGF in whole healthy (*n* = 25) and tumor-infiltrated (*n* = 29) bladder tissue lysates. **E** In vivo experimental design for ILC, mast cell and tumor cell analyses. **F** Relative expression of *Ntrk1* in ex vivo sorted *mouse* ILC subsets from healthy (*n* = 6) and tumor-infiltrated bladders at day 1 (Day 1 p.i., *n* = 3) and day 3 (Day 3 p.i., *n* = 3) post-MB49 instillation. **G** Frequency (left) and absolute numbers (right) of mast cells in *mouse* healthy (*n* = 6) and tumor-infiltrated bladders at day 1 (Day 1 p.i., *n* = 6) and day 3 (Day 3 p.i., *n* = 6) post-MB49 instillation, quantified by flow cytometry. **H** Relative expression of *Ngf* in *mouse* mast cells and tumor cells sorted from bladders of 6 individual *mice*, determined by qPCR analysis. **I** In vivo experimental design for mast cell depletion. **J** Survival curve of MB49 tumor-bearing WT *mice* treated (*n* = 10) or not (*n* = 12) with anti-cKit antibody. Data are the mean ± SEM and pooled from two to three independent experiments on independent biological replicates. *P* values were determined by Logrank (Mantel–Cox) test (**B**, **J**), two-tailed Mann–Whitney test (**D**, **H**) and two-tailed Tukey’s multiple comparisons test (**F**, **G**). Panels **A**, **C**, **E** and **I** were “Created in BioRender. Jandus, C. (2026) https://BioRender.com/xmyyvwk”. Source data are provided as a Source Data file.

To assess the in vivo relevance of our findings, we established an orthotopic *mouse* model of bladder cancer relying on the intravesical instillation of the MB49 bladder cancer cell line^52^. Validating our previous observations in BC patients^6^, we observed that BC bearing *Rorα*^*f/f*^ *IL7r*^*Cre+*^ *mice* that lack ILC2s (referred to as ILC2 KO, Fig. S5)^53^ exhibited a strong survival advantage compared to littermate controls (*Rorα*^*f/f*^ *IL7r*^*Cre-*^ *mice* referred to as littermates, LT) (Fig. 3A and 3B). In line with our *human* data, NGF was enriched in tumor-bearing *mouse* bladder lysates compared to healthy bladders (Fig. 3C and 3D). Further, ILC2s isolated from healthy bladders and tumor-infiltrated bladders at day 1 and day 3 post MB49 instillation (Fig. 3E) expressed high levels of *Ntrk1* (Fig. 3F and S6A for sorting strategy). Neither the low affinity NGF receptor nor the other neurotrophic receptors (*Ntrk*2 and *Ntrk3*) were specifically expressed by *mouse* ILC2s (Fig. S6B). Noteworthy, *Ntrk1* was not detected in ILC2s isolated from the *murine* lungs, an ILC2 rich organ (Fig. S6C for gating strategy and S6D), confirming and extending the concept of distinct ILC2 tissue programs^54^. Similar to our observations in *human* BC cell lines (Fig. S3C), the MB49 tumor cell line expressed very low level of the high affinity TrkA receptor, as compared to ILC2s (Fig. S6E). Next, similar to our approach with BC patients, we searched for the source of NGF in our orthotopic BC model. We assessed mast cell frequencies 1 and 3 days after the MB49 cell line instillation (Fig. 3G and S7A for gating strategy). Already 1 day post instillation, mast cells heavily infiltrated the bladder (Fig. 3G). Moreover, sorted mast cells from *murine* bladders showed higher levels of NGF transcripts in comparison to sorted cancer cells themselves (Fig. 3H). Notably, depleting mast cells using anti-cKit antibody injection (Fig. 3I and S7B) led to significant survival benefit in tumor bearing animals compared to untreated controls (Fig. 3J).

Altogether, our data suggest that NGF secretion by mast cells occurs in bladder cancer, as a shared phenomenon across species, promoting pro-tumorigenic ILC2 functions with a significant impact on survival.

### ILC2s are critical for Treg accumulation in the bladder

**Fig. 4:**
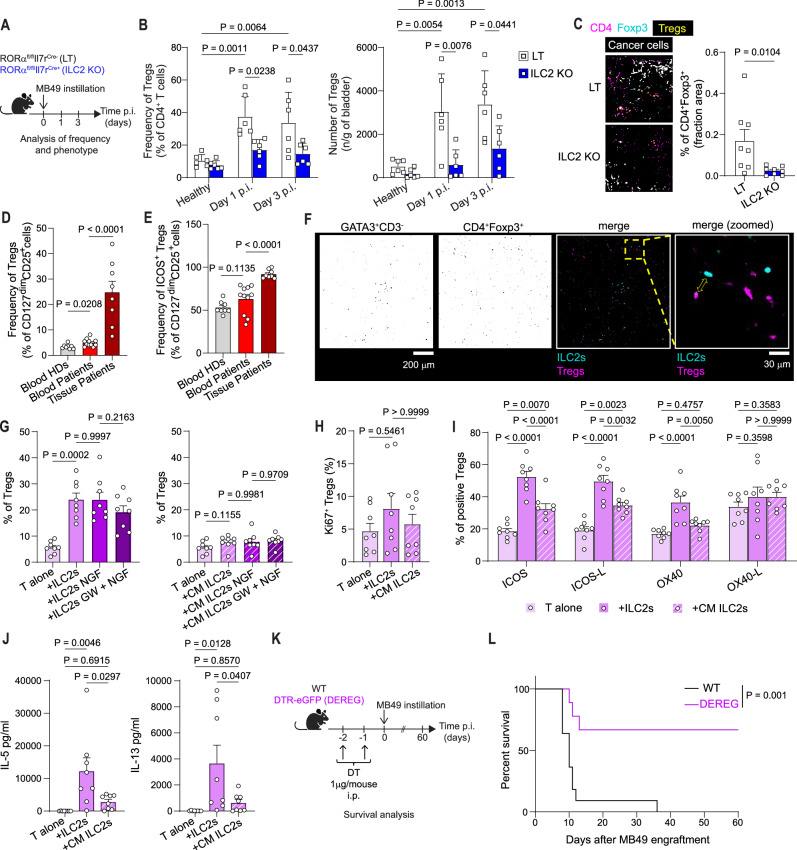
ILC2s are critical for Treg accumulation in the bladder. **A** Experimental design for tumor infiltrating immune analysis in *Rorα*^fl/fl^*Il7r*^Cre-^ (LT) and *Rorα*^fl/fl^*Il7r*^Cre+^ (ILC2 KO) *mice*. **B** Frequency (left) and absolute numbers (right) of Tregs in healthy (*n* = 6) and tumor bearing LT and ILC2 KO *mice* (*n* = 6) at day 1 (Day 1 p.i., *n* = 6), and day 3 (Day 3 p.i., *n* = 6) post-MB49 instillation. **C** Immunofluorescence of Tregs (yellow) in MB49-tumor-infiltrated LT (*n* = 8) and ILC2 KO (*n* = 8) bladders (left) and quantification (right). **D** Frequency of total and (**E**) ICOS expressing Tregs in blood of HDs (*n* = 8), BC patients (*n* = 11) and in bladder tumor tissues (*n* = 8). **F** Confocal images of ILC2s (GATA3^+^/CD3^-^) and Tregs (Foxp3^+^/CD4^+^). A dashed box marks a magnified region, and a yellow arrow highlights the ILC2-Treg proximity. Scale bar, 30 µm (*n* = 6). **G** Frequency of total, (**H**) Ki67^+^ and (**I**) phenotypic analysis of Tregs induced by naïve CD4^+^ T cell-ILC2 co-culture (left) or ILC2 conditioned-medium (CM, right) (*n* = 8 HDs). ILC2s were pre-stimulated or not with NGF, and where indicated they were pre-treated with GW441756. **J** IL-5 and IL-13 quantification in the SN from naive CD4^+^ T cells-ILC2s or CM co-cultures (*n* = 8 HDs). **K** In vivo experimental design. **L** Survival curve of MB49 tumor-bearing WT (*n* = 11) or DEREG (*n* = 9) animals. Data are the mean ± SEM from two to three independent experiments on independent biological replicates. *P* values were determined by two-tailed Mann–Whitney test (**C**), two-tailed Sidak’s multiple comparisons (**D**, **E**, **G** left graph), one-way Anova (*p* = 0.0816, **G** right graph), Kruskal–Wallis test (*p* = 0.5393, **H**), two-tailed Tukey’s multiple comparisons (**B**, **I**, **J**) and Logrank (Mantel–Cox) test (**L**). Panels **A** and **K** were “Created in BioRender. Jandus, C. (2026) https://BioRender.com/xmyyvwk”. Source data are provided as a Source Data file.

To identify the ILC2-downstream cell targets involved in bladder cancer pathogenesis, we instilled the MB49 cell line in ILC2KO and in their LT and we analyzed the immune cell composition within the bladder 1 and 3 days after tumor cell instillation (Fig. 4A). Among the immune cells analyzed (Figs. S8 and S9), beside MDSCs and eosinophils (Fig. S8A for gating strategy and S8B), Tregs were the immune cell population significantly enriched in LT compared to ILC2KO bladders, after MB49 instillation, as shown by flow cytometry and in whole bladder staining (Fig. 4B–C), indicating that the absence of ILC2s impairs the accumulation/differentiation of this cell type. As a consequence, although total T and NK/NKT cell populations did not show any significant change (Fig. S9A for gating strategy and S9B), the CD8 T cell/Treg ratio was higher in ILC2KO (Fig. S9C) and there was a trend for increased IFNγ^+^ CD8 T cells in the absence of ILC2s (Fig. S9D), possibly contributing to the heightened anti-tumor effect.

In *human*, BC patients showed higher frequency of Tregs in comparison to HDs (Fig. 4D), with a pronounced expansion of the highly suppressive ICOS^+^ subset^55^, particularly in the tumor bed (Fig. 4E). Tregs were positioned in proximity to ILC2s, suggesting a direct crosstalk (Fig. 4F). Tregs are themselves not sensing NGF since they are TrkA negative, both in HDs and patients (Fig. S10A). To test whether ILC2s were able to induce Tregs, we in vitro cultured naïve CD4^+^ T cells alone or in the presence of autologous ILC2s, or conditioned medium (CM) from autologous ILC2s, pre-stimulated or not with NGF, and pre-treated or not with TrkA inhibitor. Tregs were induced upon co-culture with ILC2s, in a contact-dependent and NGF-independent fashion (Fig. 4G). To understand the mechanism of Treg expansion we monitored Ki67 and excluded that the observed Treg increase was due to proliferation (Fig. 4H). To determine which molecules could be involved in the Treg induction upon ILC2 contact, we compared the phenotype of T cells either cultured alone, or with ILC2 or with CM. The markers preferentially upregulated in Tregs induced upon co-culture with ILC2s, but less or not at all with the CM, were ICOS and OX40, respectively (Fig. 4I), previously shown to be involved in ILC2-Treg cross-talk^56,57^. In line with our in vitro data, TCGA showed an enrichment of Treg-associated transcripts (Fig. S10B) and increase of both ICOS and OX40 expression in BC patients with a high NGF signature (Fig. S10C). In line with ICOS and OX40 upregulation on Tregs, we observed increased expression of ICOSL and OX40L on ILC2s (Fig. S10D) when co-cultured with the HMC1 line. However, blocking OX40-OX40L and ICOS-ICOSL interactions in the ILC2-Treg co-culture did not affect Treg induction (Fig. S10E), suggesting that other factors, including cytokines, produced following ILC2 and naïve T cell interaction, may contribute to sustaining Treg induction. To test this, we measured cytokine levels in the co-culture supernatant and found increased concentrations of IL-5 and IL-13 (Fig. 4J), previously shown to be involved in Treg induction^58^. In contrast, levels of IL-2 and IL-10, also known to favor Tregs, remained unchanged (Fig. S10F). To determine the functional relevance of these cytokines, we co-cultured ILC2s with naïve T cells in the presence of anti-IL-13 or anti-IL-5 antibodies. We observed a significant reduction in Treg induction with IL-13 blockade, while concomitant blockade of other molecules did not result in a significant effect (Fig. S10G). Finally, to assess the role of Tregs as the ILC2 downstream cell player sustaining tumor growth in vivo, we instilled the MB49 cell line into DEREG *mice* and observed a significant survival benefit upon Treg depletion via Diphtheria toxin administration, as compared to controls (Figs. 4K and 4L).

Altogether these observations indicate that bladder tumorigenesis favors the in-situ accumulation of ILC2s promoting the induction of Tregs. In the absence of either ILC2s or Tregs tumor growth is better controlled, supporting the existence of an ILC2-Treg axis.

### Pharmacologic TrkA inhibition favors survival in synergy with ICB in an orthotopic BC model

**Fig. 5:**
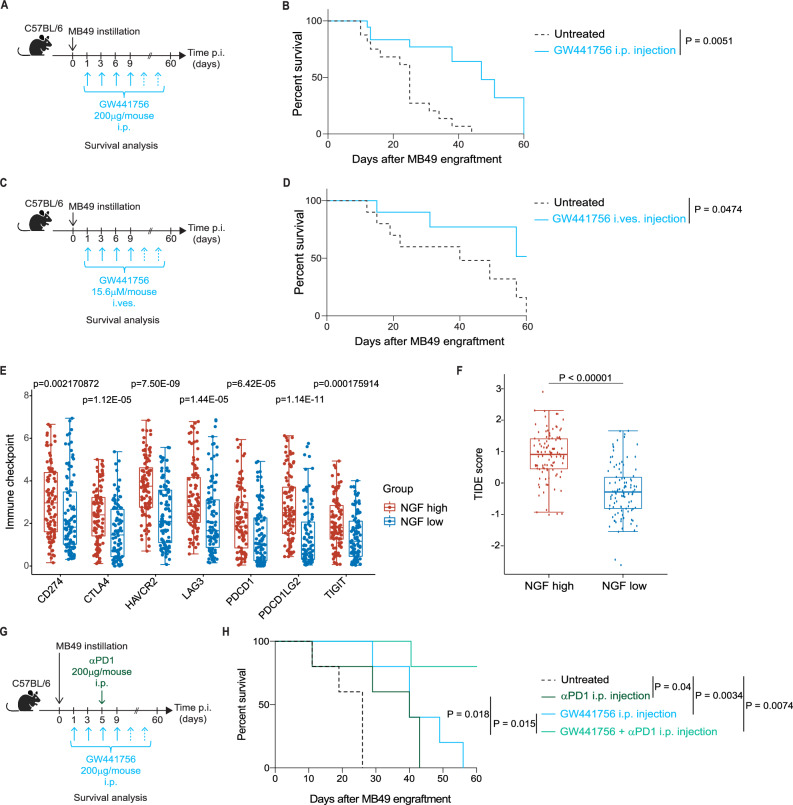
Pharmacologic TrkA inhibition favors survival in an orthotopic bladder cancer model. **A** In vivo experimental design for intraperitoneal injection of GW441756. **B** Survival curve of MB49 tumor-bearing WT *mice* treated with the TrkA inhibitor GW441756 (*n* = 18) or the solvent control (*n* = 18) intraperitoneally twice per week. **C** In vivo experimental design for intravesical delivery of GW441756. **D** Survival curve of MB49 tumor-bearing WT *mice* treated with the TrkA inhibitor GW441756 (*n* = 18) or the solvent control (*n* = 18) intravesically twice per week. **E** Immune checkpoint gene expression level in tumors of TCGA BC patients stratified into high and low *NGF* groups (*n* = 102 per group). The x-axis represents different sample groups, and the y-axis represents the distribution of gene expression, which is normalized using log2(TPM + 1). **F** TIDE scores calculated in TCGA BC patients stratified into high and low *NGF* groups (*n* = 102 per group). **G** In vivo experimental design for the combination of intraperitoneal injection of GW441756 and anti-PD1 antibody treatment. **H** Survival curve of MB49 tumor-bearing WT *mice* untreated (*n* = 5) or treated with either the TrkA inhibitor GW441756 alone (*n* = 5), or with anti-PD1 antibody alone (*n* = 5), or with a combination of both (*n* = 5). Data are the mean ± SEM pooled from one to three independent experiments on independent biological replicates. *P* values were determined by Log-rank (Mantel–Cox) test (**B**, **D**, **H**), two-tailed Wilcoxon rank-sum test (**E**) and two-tailed unpaired *t*-test (**F**). For **E**–**F**, the thick line represents the median value. The bottom and top of the boxes are the 25^th^ and 75^th^ percentiles (interquartile range). The whiskers encompass 1.5 times the interquartile range. Panels **A**, **C** and **G** were “Created in BioRender. Jandus, C. (2026) https://BioRender.com/xmyyvwk”. Source data are provided as a Source Data file.

Our data suggest that a mast cell-NGF-ILC2-Treg axis supports bladder tumor growth via NGF stimulation of TrkA^+^ILC2s, that in turn induce Tregs, favoring tumor immune escape. Therefore, we in vivo blocked the NGF-TrkA signaling by injecting the TrkA inhibitor GW441756 intraperitoneally^59^ (Fig. 5A) and evaluated *mouse* survival. We observed a significant survival advantage when MB49 tumor-bearing WT *mice* were treated with GW441756 (Fig. 5B), demonstrating that TrkA-blockade is sufficient to dampen tumor cell growth. No effect of the inhibitor was observed when administered in ILC2KO tumor bearing animals, arguing for an ILC2-dependent NGF-TrkA effect (Fig. S11A). We observed reduced ILC2s, IL-13^+^ ILC2s (Fig. S11B) and Tregs (Fig. S11C) in treated compared to untreated WT animals. Moreover, to reduce potential systemic toxicity, increase local delivery and be closer to the clinical setting, in which the conventional BCG therapy is delivered intravesically, we administered GW441756 intravesically (Fig. 5C), at concentrations not affecting tumor cell survival in vitro (Fig. S11D), and monitored animal survival. Similar to the previous experiment, *mice* receiving the TrkA inhibitor showed a survival advantage (Fig. 5D), supporting the therapeutic potential for TrkA-blockade in BC patients resistant to current treatment. Interestingly, by mining the TCGA BC patients’ dataset we observed significantly higher expression of immune checkpoint molecules in the NGF^high^ compared to NGF^low^ cohort (Fig. 5E) and low NGF concentrations predict better response to immunotherapy, as indicated by the lower Tumor Immune Dysfunction and Exclusion (TIDE) score^60^ of these patients (Fig. 5F). Therefore, we reasoned that by alleviating the local NGF-TrkA immune suppressive axis, ICB therapy would show superior efficacy. To test this, we treated tumor-bearing *mice* with GW441756 and anti-PD1 antibody (Fig. 5G), and observed synergy of the two treatments, as compared to either of the monotherapies (Fig. 5H). Importantly, analysis of the TCGA dataset confirms the deleterious impact on survival in patients with a high NGF-ILC2 enriched signature, observed in both stomach and colorectal cancers (Fig. S11E). These findings hold promising clinical implications, particularly for patients with resistant or relapsing disease in other cancer entities.

## Discussion

In the bladder, NGF has been identified as a key regulator of organ function and is involved in several bladder inflammatory pathologies^61,62^. Here, we highlight NGF as a potent regulator of tumor immunity in BC, both in patients and tumor bearing animals, and we demonstrate its mechanism of action on tissue infiltrating, pro-tumoral, TrkA^+^ ILC2s, in turn impacting Tregs. Our pre-clinical targeting of the NGF-TrkA axis and the observation that increased NGF expression correlates with poor survival in BC patients attest of its therapeutic potential for this highly deadly disease.

Neuronal-derived factors are emerging as key regulators of ILC functions, either by potentiating or by inhibiting their functions^27^. For ILC2s, significant tissue-specific molecular heterogeneity has been reported, with the expression of some receptors (e.g., IL-25, IL-33, IL-18 receptors) being restricted to a defined anatomic localization^63^. While receptors of neural derived factors and neurotransmitters (e.g., NMU, CGRP, norepinephrine) were reported in lung and gut ILC2s^14^, no significant TrkA expression was found at transcriptomic levels in ILC2s infiltrating these organs at steady state. It is tempting to speculate that the upregulation of TrkA is a peculiar event occurring in the mucosal-infiltrating ILC2s in the bladder microenvironment, or in other organs upon exposure to specific inflammatory triggers. Factors involved in its induction could include nicotine and tobacco extracts^64^, being associated with increased risk of BC^65^, or other xenobiotics known to accumulate in the bladder cavity. Alternatively, tumor derived mediators might be responsible for inducing neurotrophic factor receptors’ expression on ILC2s. If other tumor types known to be heavily ILC2 infiltrated (e.g., prostate cancer^15,20^, hepatocellular carcinoma^19^, pancreas^66^, lung^17^, gastric cancer^67^, colorectal cancer^18^) also display enriched TrkA^+^ innate lymphocytes remains to be established. Interestingly, TCGA mining of these ILC2-rich cancers for associations between survival and NGF concentrations showed strong survival reduction in patients with gastric and colorectal cancer with high NGF transcript levels and ILC2 infiltration (Fig. S11E). However, given the correlative nature of this observation, additional experimental work in these settings would be necessary to demonstrate causality.

TrkA expression was not observed homogenously in the entire ILC2 population: a variable proportion of ex vivo ILC2s are TrkA negative, with their frequency depending on the donor or the patient. It will be interesting to assess the ontogeny of TrkA expressing ILC2s. Although our side-by-side transcriptional profiling of TrkA^+^ vs TrkA^-^ ILC2s in the same individuals did not show major differences at steady state, additional transcriptomic analyses, jointly to epigenetic profiling, of these cell subsets in disease settings might provide further insights about specific functions, metabolism, or phenotype of TrkA^+^ cells that could be exploited therapeutically.

TrkA^+^ ILC2s respond to NGF by exacerbating their Type2 pro-tumoral phenotype. Besides its role in modulating neural plasticity, NGF has been described to act as an immunomodulator in different inflammatory processes, by activating immune cells^34,68^. However, in the context of tumor immunity, the role of NGF has been mainly centered on its effect on tumor cells^69^, rather than on the immune system. In breast cancer, NGF alters tumor cell migration, proliferation and invasiveness in vitro^70^ and in vivo^71^. Similar observations were made using prostate cancer models^59,72^, and high levels of the precursor of NGF has been correlated with increased Gleason score in patients with prostate cancer. In melanoma, it was recently reported on the NGF action on both malignant melanoma cells and TrkA^+^ low-affinity CD8 T cells^36^. Knowing that NGF-TrkA inhibition was shown to directly impact cancer cell proliferation, survival^35^, and sensitization to IFNγ^36^, synergistic effects can be expected by targeting tumors composed of both TrkA^+^ cancer cells and TrkA^+^ ILC2s, potentially resulting in the restoration of immune effector functions and impacting on survival.

Several cell types, including mast cells known to be present in the bladder tissue^73^, can synthetize and release NGF upon different stimuli^74^. The consequence of the observed increased NGF content in mast cells infiltrating the bladder TME is still unclear, as well as the nature of the NGF-triggers in these cells. Extracellular ATP, released by dying cells in the bladder TME, might be one of the mast cell activators, via P2X7 purinoceptors expressed on these cells, as reported in other inflamed tissues^75^. The ability to promptly secrete soluble mediators and chemoattractant positions mast cells as early interactors with several other TME components^76^. In our setting, bladder tissue-resident ILC2s, or circulating ILC2s, may be attracted to the tumor bed through a mast-cell derived NGF gradient and activated in situ to vigorously secrete type 2 cytokines. However, given that nerve fibers can also be a source of NGF^40^, and being the bladder a highly innervated tissue, we cannot exclude that nerve fibers might act as an additional source of NGF^75^ in BC.

Further, using tumor-bearing ILC2KO animals, we show that activated ILC2s shape the bladder TME, in particular by inducing MDSC accumulation, as we previously reported^6^, eosinophils and Tregs. Tumor eosinophilia is not very common but was reported in different tumors, with unclear clinical relevance. In BC, ILC2-derived IL-5 might drive local eosinophilia, that was prevented in the tumor-bearing ILC2KO animals. The presence of bladder eosinophils was reported to positively impact prognosis in patients with translational bladder tumors, but not in the cases with squamous differentiation^77^ or in recurring BC^78^, arguing for eosinophil pro-tumoral activities. Further, we found enriched Tregs in BC-bearing *mice* and in BC patients’ peripheral blood, and even more strikingly in patients’ tumor tissues. In addition, Tregs from bladder cancer patients’ blood and tumors expressed high level of ICOS, known to be upregulated by highly suppressive Tregs^79^. While these Tregs might be induced upon interaction with mast cells^80^, MDSCs^81^ or eosinophils^82^, by performing in vitro co-cultures we demonstrate that ILC2s directly convert naïve CD4 T cells into Tregs. This process depends on an initial cell-cell interaction, which triggers IL-13 secretion and subsequently induces Tregs.

Lastly, the in vivo TrkA blockade resulted in improved survival of tumor-bearing *mice*, when the inhibitor was delivered either intraperitoneally or intravesically. A number of TrkA inhibitors are currently tested in the clinics for treatment of chronic pain^83^ or other malignancies^84^, with very good safety profiles. The conventional therapy for NMIBC being the intravesical delivery of BCG, we can speculate that the use of TrkA inhibitors for non-BCG-responsive patients or as alternative to BCG may increase response rates and prevent relapse and disease progression, a major challenge in BC. Furthermore, while immune checkpoint blockers were introduced for the treatment of MIBC and five of them have been approved by the Food and Drug Administration^85^, only a limited number of patients respond, despite the positive predictive score of PD1/PD-L1 and the high tumor mutational burden of this cancer type^86^. Our in vivo combination treatments’ results suggest that the use of TrkA inhibitors might pre-condition NGF^high^ BC patients, or patients with other NGF^high^ tumors, for subsequent treatment with anti-PD-1/PDL1 blocking agents, rendering non-responsive tumors amenable to immune checkpoint blockade.

## Methods

### Human peripheral blood cell collection

Venous blood from healthy donors (HDs) was purchased from the local blood transfusion center, Geneva, Switzerland, and all subjects gave their written consent. Peripheral blood mononuclear cells (PBMCs) were freshly isolated by Lymphoprep (Promega) centrifugation (600 g, 20 min, without break, room temperature). Red blood cell lysis was performed using red blood lysis buffer (Qiagen) and platelets were removed by centrifugation (188 g, 10 min without break, room temperature). Cells were counted and immediately used or cryopreserved until use.

### Bladder cancer patient samples

Blood and tumor specimens were freshly obtained from patients with bladder cancer from the University Hospital of Geneva in the frame of the EC 2020-02375 protocol approved by the local Ethical Committee and upon written informed consent. Blood samples and serum were collected prior the surgery and tumor samples were excised by surgeons in the frame of trans urothelial resection of the bladder tumor (TURBT) or during cystectomy. Samples were immediately processed and used or cryopreserved until use. Patients’ clinical information is summarized in Supplementary Table 1.

Bladder cancer patients’ tissue sections were obtained from the Biobank of Institute of Pathology at the CHUV (study n°2019/00882) and in the frame of the study protocol n127/2022-DB id12223 for patients with bladder cancer and n. 39/2012 for healthy donors, approved by the ethics committee of the Liguria Region, Genova, Italy. All subjects gave written informed consent in accordance with the Declaration of Helsinki.

Urine samples and tumor lysates from patients with bladder cancer were obtained in the frame of the study protocol N82/19, approved by the Ethics Committee for Biomedical Research from the Faculty of Medicine and Pharmacy of Rabat-Morocco, upon written informed consent. Urine samples were centrifuged (2300 g, 15 min) and supernatant was collected and cryopreserved until use. Patients’ clinical information is summarized in Supplementary Table 1.

PBMCs were freshly isolated by Lymphoprep (Promega) centrifugation (600 g, 20 min, without break, room temperature). Red blood cell lysis was performed using red blood lysis buffer (Qiagen) and platelets were removed by centrifugation (188 g, 10 min without break, room temperature). Cells were counted and immediately used or cryopreserved until use.

Tumor biopsies were freshly collected in Leibovitz’s L-15 medium (Gibco), supplemented with Hepes (Gibco), Glucose (Sigma) and penicillin-streptomycin (Gibco). Small tumor pieces were cut and digested in complete Leibovitz’s L-15 medium containing 2.5 mg/ml of Liberase TM (Roche) for 30 min at 37 °C. For big tumor pieces, tumors were dissociated in complete Leibovitz’s L-15 medium containing 2.5 mg/ml of Liberase TM (Roche) using the gentleMACS™ dissociator instrument (Miltenyi), following the manufacturer’s protocol. Cell suspension was filtered using a 100 µm strainer and red blood cells lysis was performed using red blood cell lysis Buffer (Qiagen). Cells were then ready to be stained for flow cytometry analysis or were cryopreserved until use.

### Bladder tissues from cadaveric donors

Healthy bladder tissues were obtained from the Center Universitaire Romand de Médecine Légale (CURML) in the frame of the study protocol VD431/13 approved by the local Ethical Committee (CER-VD). Samples were collected from deceased donors in the course of an autopsy, and anonymised for research purposes, not requiring consent, in accordance with Chapter 5, Article 38 of the Swiss Human Research Act.

### Isolation of ILCs from PBMCs

ILCs were isolated from fresh PBMCs using a FACS Aria II (BD Biosciences). *Human* ILCs were identified as lineage (Lin) negative and CD127 positive cells. Lineage markers, all FITC-conjugated, include: anti-*human* CD3 (UCHT1, 1:200, Biolegend, Cat 300406, Lot B390953), anti-*human* CD4 (RPA-T4, 1:200, Biolegend, Cat 300538, Lot B374653), anti-*human* CD8 (SK1, 1:200, Biolegend, Cat 344704, Lot B367025), anti-*human* CD14 (HCD14, 1:400, Biolegend Cat 325604, Lot B416698), anti-*human* CD15 (HI98, 1:50, Biolegend Cat 394706, Lot B426569), anti-*human* CD16 (3G8,1:400, Biolegend Cat 302006, Lot B368715), anti-*human* CD19 (HIB19, 1:100, Biolegend Cat 392508, Lot B373946), anti-*human* CD20 (2H7, 1:400, Biolegend Cat 302304, Lot B373724), anti-*human* CD33 (HIM3-4, 1:400, Biolegend Cat 303304, Lot B396884), anti-*human* CD34 (581, 1:100, Biolegend Cat 343504, Lot B407007), anti-*human* CD203c (NP4D6, 1:25, Biolegend Cat 324614, Lot B372625) and anti-*human* FcεRIα (AER-37, 1:200, Biolegend Cat 334608, Lot B302018). Additional markers used for ILC subsets’ identification and characterization include: Brilliant Ultraviolet 395 (BUV395) anti-*human* CD45 (HI30, 1:100, BD Biosciences Cat 563792, Lot 3268377), Brilliant Violet 421 anti-*human* CD127 (IL-7Rα) (A019D5, 1:100, Biolegend Cat 351310, Lot B396895), Brilliant Violet 605 anti-*human* CD117 (c-Kit) (104D2, 1:200, Biolegend Cat 313218, Lot B362518), Alexa Fluor 647 anti-*human* CRTH2 (CD294) (BM16, 1:200, Biolegend Cat 350104, Lot B373153) and PE anti-human TrkA (REA430, 1: 100, Miltenyi Cat 130-117-705, Lot 5240509055). For some experiments, lineage markers were used as follows: PerCP-Cyanine5.5 anti-*human* CD3 (UCHT1, 1:200, Biolegend Cat 300430, Lot B361672), PerCP-Cyanine5.5 anti-*human* CD8 (SK1, 1:100, Biolegend Cat 344710, Lot B330644), PerCP-Cyanine5.5 anti-*human* CD14 (HCD14, 1:100, Biolegend Cat 325622, Lot B197134), PerCP-Cyanine5.5 anti-*human* CD16 (3G8, 1:800, Biolegend Cat 302028, Lot B337563) and PerCP-Cyanine5.5 anti-*human* CD19 (H1B19, 1:50, Biolegend Cat 302230, Lot B361746), PE-Cyanine7 TCRα/β (IP26, 1:100, Biolegend Cat 306719, Lot B298943) and PE-Cyanin7 TCRγ/δ (B1, 1:100, Biolegend Cat 331221, Lot B318208). Dead cells were excluded using the viability Zombie Green Dye (1:2000, Biolegend Cat 423112, Lot B314570).

### Bulk mRNAseq on TrkA^+^ and TrkA^-^ ILC2s

Total RNA was extracted using the RNEasy Micro Plus Kit (Qiagen) according to the manufacturer’s instructions. Briefly, cells were lysed in RLT Plus buffer containing β-mercaptoethanol and genomic DNA was removed using a gDNA Eliminator spin column. RNA was purified via silica membrane spin columns with DNase digestion, washed, and eluted in RNase-free water. Total RNA was quantified with a Qubit (fluorimeter from Life Technologies) and RNA integrity assessed with a Bioanalyzer (Agilent Technologies). The SMART-Seq mRNA kit from Takara was used for reverse transcription and cDNA amplification according to manufacturer’s specifications, starting with 2 ng of total RNA as input. 200 pg of cDNA were used for library preparation using the Nextera XT kit from Illumina. Library molarity and quality were assessed with the Qubit and Tapestation using a DNA High sensitivity chip (Agilent Technologies). Libraries were sequenced on a NovaSeq 6000 Illumina sequencer for SR100 reads.

The raw data were imported into the Subio Platform 64 for quality control, trimming, alignment (hg38), and counting (default setting). The counting matrix first manually sorted and then imported into iDEP 2.01 platform for normalization and differentially expressed genes (DEGs) analysis. Genes with minimal counts (0.5) per million (CPM) in at least 1 library were kept for downstream analysis. Counts were transformed by EdgeR: log2(CPM+c). DEGs were defined by DESeq2, using fold of change (FC) > 2, False Discovery Rate (FDR) = 0.1. RNAseq data of *human* ILC2s have been deposited in NCBI’s Gene Expression Omnibus and are accessible through GEO accession number GSE311046.

### Human ILC2 culture

Sorted *human* ILC2s were short-term (3-4 weeks) expanded in RPMI 1640 (Gibco), supplemented with 8% *human* serum (HS), 1% penicillin-streptomycin (Gibco), 1% L-Glutamine (Gibco), 10 mM Hepes (Gibco), 1% Kanamycin (Gibco), 0.1% β-mercaptoethanol (Gibco), 1% non-essential amino acide and 1% Na-Pyruvate with the addition of with 200U/ml recombinant *human* interleukin (IL)−2 (Roche), 10 ng/ml of recombinant *human* IL-7 (Peprotech) and 1 µg of Phytohaemagglutinin (PHA) at day 0 (Sigma). The medium was changed every 2 days. Cells were grown at 37 °C with 5% CO_2_.

*Human* bladder cell line HCV29 (Cat CVCL_8228) and bladder cancer cell lines: RT-4 (Cat HTB-2™), J82 (Cat HTB-1™), and TCC-Sup (Cat HTB-5™) were cultured in RPMI 1640 (Gibco), supplemented with 10% FCS (Gibco), 1% penicillin-streptomycin (Gibco), 10 mM Hepes (Gibco), 0,05 mg Cyproxin (Bayer) and a mix of L-Arginine, L-Asparagine and L-glutamine (AAG). Cells were grown at 37 °C with 5% CO_2_.

For NGF stimulation experiment, ILC2s were seeded at 2000 cells per well and stimulated with 10 ng/ml of recombinant *human* NGF (Peprotech). Supernatants and cell pellets were collected after 48 hours of stimulation.

For TrkA inhibition experiments, ILC2s were pre-treated with 10 µM of GW441756 (Selleckchem) for 45 min, washed and then stimulated with 10 ng/ml of NGF for 48 h.

The *human* mast cell line HMC1 (Cat SCC067, Sigma Aldrich) was purchased from Sigma-Aldrich and cultured in RPMI 1640 (Gibco), supplemented with 10% FCS (Gibco), 1% penicillin-streptomycin (Gibco), 10 mM Hepes (Gibco), and 50 μM β-mercaptoethanol (Sigma). Cells were grown at 37 °C with 5% CO_2_.

For mast-cell-ILC2 co-culture experiments, ILC2s were seeded at 5000 cells per well and co-cultured with HMC1 cells at 1:1 ratio, previously stimulated with 50 nM of Phorbol-12- myristate-13-acetate (PMA) (Sigma-Aldrich) and 10 μM of Ionomycin (Sigma-Aldrich), for 6 h, and extensively washed before co-culture. Control wells with either ILC2 alone or stimulated HMC1 alone were also included. Supernatants were collected after 48 h of stimulation, when ILC2s were stained for ICOSL and OX40L expression. For TrkA inhibition experiments, ILC2s were pre-treated with 10 µM of GW441756 (Selleckchem) for 45 min, washed and then co-cultured with HMC1 for 48 hours.

### Isolation of naïve CD4^+^ T cells from PBMCs

*Human* naïve CD4^+^ T cells were isolated from fresh PBMCs using a FACS Aria II (BD Biosciences) as CD3^+^CD4^+^CD45RA^+^. The following antibodies were used: BUV395 anti-*human* CD3 (SK7, 1:200, BD Cat 563546, Lot 1214287), PerCP-Cyanine5.5 anti-*human* CD4 (OKT4, 1:100, Biolegend Cat 317428, Lot B326199) and FITC anti-*human* CD45RA (HI100, 1:200, Biolegend Cat 304105).

### Regulatory T cell induction experiment

Donor-matched ILC2s (either pre-stimulated or not with NGF for 48 h, and either pre-treated or not with 10 µM of GW441756 for 45 min prior NGF exposure) were co-cultured with naïve CD4^+^ T cells at a ratio 1:10. Culture medium of ILC2s prior co-cultures was collected to be used as conditioned medium (CM) at 50/50 ratio with RPMI culture medium on donor-matched naïve CD4^+^ T cells. After 6 days, CD4^+^ T cell phenotype was checked by flow cytometry, using the LSR Fortessa Flow Cytometer (BD Biosciences). Data were analyzed using FlowJo (v. 10.7.1). Supernatant was collected for cytokine quantification at the same time point as cell harvesting. Where indicated, 10 μg/ml of Ultra-LEAF purified anti-*human* OX40 (W22033K, Biolegend Cat 632653, Lot B418814), anti-*human* ICOSL (W21036I, Biolegend Cat 387505, Lot B407137), anti-*human* IL-5 (TRFK5, Biolegend Cat 504313, Lot B461188), anti-*human* IL-13 (JES10-5A2, Biolegend Cat 501918, Lot B294618) were added on day 1 of the co-culture.

### Flow Cytometry analysis on human cells

Additional markers used for Treg immune phenotyping include PE anti-*human* TrkA (REA430, 1:100, Miltenyi Cat 130-117-705, Lot 5240509055), PerCP-Cyanine 5.5 anti-*human* ICOS (C398.4 A, 1:50, Biolegend Cat 313518, Lot B272324), FITC anti-*human* CD45RA (HI100, 1:200, Biolegend Cat 317428), PE Dazzle 594 anti-*human* CD127 (A019D5, 1:100, Biolegend Cat 351336, Lot B241297), Brilliant Violet 510 anti-*human* CD25 (BC96, 1:100, Biolegend Cat 302640, Lot B366218), PE-Cy7 anti-*human* ICOSL (2D3, 1:50, Biolegend Cat 309410, Lot B295275), PreCP-Cy5.5 anti-*human* OX40 (Ber-ACT35, 1:50, Biolegend Cat 350010, Lot B279300), Brilliant Violet 786 anti-*human* OX40L (11C3.1, 1:200, BD Biosciences Cat 743236, Lot 5268391) and Brilliant Violet 711 anti-*human* Ki67 (Ki-67, 1:200, Biolegend Cat 350516, Lot B468646). Intracellular staining was performed after fixation and permeabilization of the cells with the Foxp3 Transcription Factor Staining Buffer Set (00-5523-00, Invitrogen) using Alexa 700 anti-*human* FoxP3 (259D/C7, 1:50, BD Biosciences Cat 566935, Lot 2165173).

Phosphorylation staining was performed after fixation with Cytofix Fixation Buffer (BD Biosciences, 554655) and permeabilization with BD Phosflow™ Perm/Wash Buffer I (BD Biosciences, 557885) using PE anti-*human* RPS6 (Ser235/236, 1:25, Biolegend Cat 608603, Lot B398629).

For mitochondrial staining, ILC2s were stained by MitoTracker™ Green FM (Invitrogen), MitroTracker®Deep Red FM (Life Technologies) and TMRM (ThermoFisher) in RPMI 1640 medium without phenol red (Gibco), supplemented with 5% of FCS.

Samples were acquired on LSR Fortessa Flow Cytometer (BD Biosciences) and data were analyzed using FlowJo (v. 10.7.1).

### Quantitative polymerase chain reaction (qPCR)

Total RNA was isolated from cells using the TRIZOL reagent according to the manufacturer’s instructions (Invitrogen) and was reverse-transcribed with the iScript Reverse Transcription Supermix for RT-qPCR (Bio-Rad). The quantitative real-time PCR was carried out in the Applied Biosystems 7900HT Fast Real-Time PCR Sequence Detection System (Applied Biosystems) with specific primers: *human* beta-2-microglobulin (*β2M*) (5’- GAGGCTATCCAGCGTACTCCA-3’; 5’-CGGCAGGCATACTCATCTTTT-3’), *human* tropomyosin receptor kinase A (*NTRK1*) (5’-AACCTCACCATCGTGAAGAGT-3’; 5’-TGAAGGAGAGATTCAGGCGAC-5’), *human* tropomyosin receptor kinase B (*NTRK2*) (5’-ACCCGAAACAAACTGACGAGT-3’; 5’-AGCATGTAAATGGATTGCCCA-3’), *human* tropomyosin receptor kinase C (*NTRK3*) (5’-ACGAGAGGGTGACAATGCTG-3’; 5’-CCAGTGACTATCCAGTCCACA-3’), *human* nerve growth factor receptor (NGFR)/p75 neurotrophin receptor (*p75NTR*) (5’-CCTACGGCTACTACCAGGATG-3’; 5’-CACACGGTGTTCTGCTTGT-3’), *mouse* hypoxanthine phosphoribosyl transferase 1 (*Hrpt*) (5’-CCCAGCGTCGTGATTAGTGATG-3’; 5’-TTCAGTCCTGTCCATAATCAGTC-3’), *mouse* tropomyosin receptor kinase A (*Ntrk1*) (5’-GGC GATGACGTGTTTCTG C-3’; 5’-AGGAGACGCTGACTTGGACA-3’), *mouse* tropomyosin receptor kinase B (*Ntrk2*) (5’-CTGGGGCTTATGCCTGCTG-3’; 5’-AGGCTCAGTACACCAAATCCTA-3’), *mouse* tropomyosin receptor kinase C (*Ntrk3*) (5’ CTGAGTGCTACAATCTAAGCCC-3’; 5’-CACACCCCATAGAACTTGACAAT-3’), *mouse* nerve growth factor receptor (NGFR)/p75 neurotrophin receptor (*p75ntr*) (5’-CGAATGCGAGGAGATCCCTG-3’; 5’-GTCACCGTATCGGCCACTG-3’), *mouse* Nerve Growth Factor (*ngf*) (5’- CCTTGGCAAAACCTTTATTGG-3’; 5’- CCAGTGAAATTAGGCTCCCTG-3’) and using KAPA SYBR® FAST qPCR Kits (Roche). Samples were amplified simultaneously in duplicate and the Ct value for each sample was determined. The housekeeping genes *human β2M* and *mouse Hrpt* were used as an internal control to normalize the Ct values, using the 2^−ΔCt^ formula, in *human* and *mouse* samples, respectively.

### Proliferation assay

ILC2s were stained with Cell Trace™ Far Red Cell Proliferation Kit (Invitrogen) for 15 min at 37 °C, washed and incubated 5 min on ice. After a wash, ILC2s were plated and stimulated for 5 days. After 5 days, proliferation was quantified by flow cytometry using LSR Fortessa Flow Cytometer (BD Biosciences) and data were analyzed using FlowJo (v. 10.7.1).

### MTT assay

Toxicity of the TrkA inhibitor GW441756 was determined by MTT (3-(4,5-dimethylthiazol-2-yl)−2,5-diphenyltetrazolium bromide) assay. Briefly, ILC2s cells were seeded in 96-well plates (10000 cells/well in 200 µl) in triplicate and treated with different concentration of GW441756 (Selleckchem). After 48 h of incubation, 25 µL of MTT was added in each well (Sigma-Aldrich; 5 mg/mL in PBS). Cells were then incubated for additional 3 h at 37 °C. After this time interval, violet crystals were solubilized with 100 µl of pure DMSO (Sigma). The optical density of each well was measured with a microplate spectrophotometer (TitertekMultiskan MCC/340), equipped with a 570 nm filter.

### Viability assay

ILC2s unstimulated or stimulated with NGF were stained using FITC Annexin V Apoptosis Detection Kit with 7-AAD (Biolegend). Briefly, ILC2s were washed and resuspended in Annexin V Binding Buffer, before adding the FITC Annexin V and 7-AAD Viability Staining Solution. Samples were immediately acquired using LSR Fortessa Flow Cytometer (BD Biosciences) and data were analyzed using FlowJo (v. 10.7.1.)

### Cytokine quantification

The concentrations of various cytokines in cell supernatants, healthy donors’ and patients’ sera were determined using the multiplex immunoassay LEGENDplex™ (Biolegend) *human* Th Panel (12-plex) and *human* Th cytokine Mix and Match Subpanel (customized 5-plex) kits. Samples were acquired using the Attune NxT instrument and data were analyzed with the LEGENDplex™ Data Analysis software (v. 8.0).

### NGF quantification in human samples

The concentration of *human* NGF in cell supernatants, healthy donors’ and patients’ sera, urine and tissue was measured using the NGF 1-plex analyte flow assay kit (Biolegend). Samples were acquired using the Attune NxT instrument and the results were analyzed with the LEGENDplex™ Data Analysis software (v. 8.0).

### Immunofluorescence and immunohistochemical analyses of human cells and sections

Expanded *human* ILC2s were fixed with methanol and after a wash, cells were incubated overnight with the anti-TrkA polyclonal antibody (PA5-98018, Thermofisher), dilution 1:100 in PBS-tween with BSA (Sigma). The secondary FITC-conjugated antibody (Invitrogen) and DAPI were incubated for 1 h. Cells were washed, mounted and the images were captured with the Axiocam Fluo microscope (Zeiss).

Bladder cancer patient sections were deparaffined before undergoing antigen retrieval step. After the blockage of the non-specific sites, the monoclonal antibody TPSAB1 (MA5-11711, Invitrogen), dilution 1:1000 was incubated for 30 minutes. The secondary FITC-conjugated antibody (A11001; Invitrogen) and DAPI (D3571, Invitrogen) were incubated for 30 min. AutoFluo Quench (R37630, Invitrogen) was applied for 1 min. Finally, sections were washed, mounted and the images were captured with the Pannoramic scan 250 Flash II (3DHistech).

Immunohistochemical colocalization was performed by using rabbit nerve growth factor antibody (N-6655, dilution 1:100, Sigma Aldrich) and *mouse* monoclonal mast cell tryptase (G3) antibody (1 ml prediluted 342M-17, Sigma Aldrich) on FFPE sections of bladder cancer patient sections. For staining, a Tyramide SuperBoostTM kit with AlexaFluorTM 488 Tyramide (Invitrogen) was used as follows. Peroxydase activity was blocked using Blocking Buffer for 60 min at room temperature. Then sections were incubated with both primary antibodies diluted in 1% bovine serum albumin (BSA) overnight at 4 °C. Tryptase protein (red) was detected with goat anti-*mouse* Alexa FluorTM 568. For NGF (green) detection, slides were processed with Alexa Fluor™ 488 Tyramide SuperBoost™ Kit - Goat anti-rabbit IgG. Nucleus was labeled with Hoechst 33258 (blue) (Thermofisher). Images were taken on an IX71 inverted microscope (Olympus, Hamburg, Germany) equipped with a ColorViewII camera (Olympus, Hamburg, Germany). Merged images were assembled into figures using ImageJ.

HMC1 cells were fixed with methanol for 5 min at −20 °C, then centrifuged, and incubated with acetone for 5 min at −20 °C. The cells were spun down for 5 minutes in a microcentrifuge, and the cell pellets were washed in PBS. Non-specific staining was blocked by adding a blocking buffer (10% normal serum, 0,3% Triton X-100). HMC1 cells were incubated overnight with primary antibodies diluted in blocking buffer at 4 °C (rabbit nerve growth factor antibody (N-6655, dilution 1:100, Sigma Aldrich; and *mouse* monoclonal mast cell tryptase antibody, (G3), prediluted, 342M-17, Sigma Aldrich). Tryptase was detected with anti-*mouse* Alexa Fluor 568, and NGF was detected with anti-rabbit Alexa Fluor 488. Nuclei were labeled with Hoechst 33258 (Thermofisher). Images were taken on an IX71 inverted microscope (Olympus, Hamburg, Germany) equipped with a ColorViewII camera (Olympus, Hamburg, Germany). Merged images were assembled into figures using ImageJ.

### Tissue localization and expression level analysis using confocal fluorescence microscopy

To investigate spatial distribution and relative expression levels, immunofluorescence staining was performed on sections of *human* healthy and cancerous bladder tissue, followed by high-resolution confocal fluorescence imaging. Staining was carried out using individually labeled sections, with appropriate primary and fluorophore-conjugated antibodies. Imaging was performed with a spinning disk confocal microscope equipped with a 20 × /1.0 NA objective. For each sample, two fields of view were acquired, each composed of a 10 × 10 tile scan. Three-dimensional image stacks were acquired and subsequently maximum projected for further analysis. ILC2s were identified based on GATA3 expression using a *mouse* monoclonal anti-*human* GATA3 antibody (L50-823, Biocare Medical), followed by detection with a secondary anti-*mouse* antibody conjugated to Alexa Fluor 647 (Invitrogen). To exclude GATA3⁺/CD3⁺ T cells from analysis, sections were co-stained with anti-*human* CD3 conjugated to Brilliant Violet 421 (UCHT1, Biolegend), and logical subtraction was performed using the Image Calculator function in ImageJ to isolate GATA3⁺/CD3⁻ ILC2s. TrkA expression in ILC2s was assessed using a PE-conjugated anti-*human* TrkA antibody (REA430, Miltenyi), and colocalization was evaluated based on overlapping fluorescence intensity profiles. Mast cells were identified using an anti-*human* TPSAB1 antibody (6A10G1, Invitrogen), and NGF expression within mast cells was detected using a rabbit anti-*human* NGF antibody (clone S179-01, ThermoFisher), followed by a secondary anti-rabbit Alexa Fluor 546 antibody (Invitrogen). Colocalization of NGF and TPSAB1 signals was confirmed using intensity line profiling. Regulatory T cells (Tregs) were identified based on co-expression of CD4 and FOXP3, using anti-*human* CD4 conjugated to Alexa Fluor 488 (RPA-T4, Biolegend) and anti-*human* FOXP3 conjugated to Alexa Fluor 594 (PCH101, ThermoFisher). For quantification of NGF expression, background subtraction was applied using the rolling ball algorithm as described above. Binary masks were generated to define individual mast cells, and these masks were used to measure NGF fluorescence intensity within each cell. Mean NGF intensity was then calculated per mast cell and normalized to local background levels. Spatial proximity between ILC2s and mast cells, as well as between ILC2s and Tregs, was qualitatively assessed based on merged confocal images.

### Impact of NGF expression level on survival of patients with BC

We determined the association between BC patient survival and NGF expression using public bulk RNAseq and microarray datasets containing muscle-invasive BC samples with available survival data. Overall survival (OS) analysis was performed on a total of 572 MIBC patients from three datasets pooled, namely TCGA BLCA^37^, GSE31684^38^ and GSE48075^39^. Progression-free survival (PFS) was explored on TCGA BLCA and GSE31684 pooled together with a total of 499 patients. Survival analysis was performed using the survival package for R (v. 4.2.). The coxph function was used for Cox regression analysis. Patients were categorized into four quartiles based on their expression levels of NGF. Cox regression analysis stratified per quartile was then performed for NGF. Cox proportional hazard ratio and the respective *p*-value were calculated for NGF based on the expression level as a continuous variable.

### Expression level of neurotrophic receptors in human ILCs

The levels of expression of genes coding for neurotrophic receptors in *human* ILCs were assessed by mining previously published bulk and single-cell RNAseq data (ArrayExpress accession E-MTAB-8494; GEO accession GSE112591^44^; GEO accession GSE150050^45^). For the two bulk RNAseq datasets, the gene expression data of each ILC subset was normalized and converted to log_2_(counts per million) as described in ref. ^43^. For the single-cell RNAseq dataset, the raw counts of peripheral blood ILCs were converted to ln(normalized counts+1) using the Seurat package (v.4.0.3^87^) for R.

One value of expression per ILC subset per gene was obtained by averaging the biological replicates of each subset, and a heatmap was drawn using the ComplexHeatmap package (v. 1.20.0^88^), for each RNAseq dataset separately.

### TPSAB1, immune checkpoint- and Treg-associated gene expression in BC tumors

The transcriptome profiling (HTSeq counts) and the clinical data of BC tumor samples of the TCGA cohort were downloaded for 412 cases using the TCGAbiolinks package (v. 2.10.5^89^) for R. Cases were filtered to only retain the ones that derived from primary tumors (sample type 01 A) and that had clinical data present, resulting in 405 retained samples. Next, we filtered the genes to retain the ones that were detected at a minimum of 2 counts per million (cpm) in at least 2 samples. We estimated size factors using the Trimmed mean of M value method implemented in the edgeR package (v. 3.24.3^90^), and converted the raw gene expression counts into log_2_(cpm) using the voom function of the limma package (v. 3.38.3^91^). The association between the expression of *NGF* and *TPSAB1* per sample was determined by calculating Pearson’s correlation coefficient.

We also stratified the TCGA patients into a high-*NGF* and a low-*NGF* expression group (*n* = 102 per group). We compared the expression level of immune checkpoint genes (*CD274, CTLA4, HAVCR2, LAG3, PDCD1, PDCD1LG2, TIGIT*), of Treg-associated genes (*CD3E, CD3D, CD3G, CD4, FOXP3*), *ICOS* and *TNFRSF4* between the 2 groups using a Wilcoxon rank sum test for each gene.

#### Mice

Wild-type *mice* C57BL/6 were purchased from the Jackson laboratory. *Rora*^*fl/fl*^*Il7r*^*Cre/+*^ *mice* were kindly provided by Prof. A. McKenzie^53^. *Mice* used in this study were 8–12 weeks old females. All *mice* were bred and maintained in accredited animal facilities and all animal experiments were performed in compliance with relevant ethical guidelines for the use and the care of laboratory animals. Animals were maintained under a 12 h dark/light cycle, at 21 °C ± 1 °C and 55 ± 10% of relative humidity. Tumor progression was closely monitored according to an established and approved scoring system. Because tumor burden could not be directly measured due to tumor location, body weight, clinical signs, hydration status and behavioral parameters were assessed daily to ensure animal welfare and to determine humane endpoints. The monitoring was done as described and approved by the Veterinary Authority of the Swiss Geneva Canton (authorizations GE4 and GE300) and predefined burden limits were not exceeded.

### In vivo antibody injections

Mast cells were depleted by intravenous administration of 500 µg/*mouse* of InVivo anti-c-Kit antibody (BioXcell, clone ACK2) at day −7 and day +1 post MB49 instillation. Animals were monitored according to the GE300 license protocols and survival was assessed. Tregs were depleted by intraperitoneal injection of 1 µg/*mouse* of Diphtheria Toxin (DT) (Sigma) at day −2 and day −1 pre MB49 instillation. Animals were monitored according to the GE300 license protocols and survival was assessed.

### Mouse cell culture

The *murine* MB49 bladder tumor cell line (Cat SCC148, Sigma Aldrich) was cultured in DMEM GlutaMAX (Gibco), supplemented with 10% fetal calf serum (Gibco) and 5% penicillin-streptomycin (Gibco). Cells were grown at 37 °C with 5% CO_2_.

### MB49 tumor instillation and treatments

*Mice* were deeply anesthetized with a solution of ketamine (50 mg/ml) and xylazine (20 mg/ml) and were urethral catheterized using a 24-gage catheter (BD Insyte). After a 15 min pre-treatment with 50 µl of a 22% ethanol solution and a wash with 50 µl of PBS, 0.5 ×106 MB49 bladder tumor cells (Merck) were instilled in 50 µl of PBS. The catheter was removed 15 min after the instillation. *Mice* were weighted every 2 days and observed every day and scored based on pre-defined criteria. *Mice* were either monitored for survival studies or scarified at the indicated time points for immune cell phenotyping.

When indicated, 10 mg/kg per *mouse* of the TrkA inhibitor GW441756 (Selleckchem) or the solvent control was intraperitoneally injected twice a week. InVivo anti-PD1 (BioXcell, clone RMP1-14) treatment was done intraperitoneally using 200μg/*mouse* at day 5 post MB49 instillation, combined with 10 mg/kg of GW441756 intraperitoneally twice per week, when indicated. For the intravesical instillation of the TrkA inhibitor GW441756 (Selleckchem), *mice* were anesthetized with isoflurane and urethral catheterized using a 24-gage catheter (BD Insyte). 50 µl of 15,6 µM of GW441756 or the DMSO control solutions were instilled. The catheter was removed 5 min after the instillation.

### Isolation of murine ILCs

*Murine* ILCs were isolated from bladders and lungs. Bladder were cut in pieces and digested with Liberase TM (Roche) and DNAse I (Invitrogen) at 37 °C for 20 min, followed by the lysis of the red blood cells with a lysis buffer (Qiagen). For lung ILC isolation, lungs were cut in pieces and digested with Liberase TM (Roche) and DNAse I (Invitrogen) at 37 °C for 30 min, followed by a Lymphoprep (Promega) centrifugation (600 g, 20 min, without break, room temperature). The lysis of the red blood cells with a lysis buffer (Qiagen) was additionally performed. Cell suspension was filtered using a 100 µm strainer and then stained for cell sorting.

ILCs were sorted using a FACS Aria II (BD Biosciences) and were identified as lineage negative, CD90.2 positive cells. Lineage markers, all FITC-conjugated include: anti-*mouse* CD3e (REA641, 1:800, Miltenyi Cat 130-119-798, Lot 5191212768), anti-*mouse* CD5 (53-7.3, 1:100, Miltenyi Cat 130-102-574, Lot 52107000024), anti-*mouse* CD8a (53-6.7, 1:200, Miltenyi Cat 130-118-468, Lot 52107000103), anti-*mouse* CD11b (M1/70.15.11.5, 1:100, Miltenyi Cat 130-113-796, Lot 5191212765), anti-*mouse* CD11c (REA754, 1:1600, Miltenyi Cat 130-110-700, Lot 5191212780), anti-*mouse* CD19 (6D5, 1:800, Miltenyi Cat 130-119-800, Lot 5191212715), anti-*mouse* B220 (RA3-6B2, 1:400, Miltenyi Cat 130-118-462, Lot 5191212709), anti-*mouse* TCRδ/γ (GL3, 1:50, Miltenyi Cat 130-104-015, Lot 5160322159), anti-*mouse* TCRβ (REA318, 1:400, Miltenyi Cat 130-104-812, Lot 5191212689), anti-*mouse* TER119 (Ter-119, 1:800, Miltenyi Cat 130-117-538, Lot 5191212704) and FcεRIα (36951, 1:50, Miltenyi 130-102-264, Lot 5191212685).

Additional markers used for ILC subsets’ identification include Brilliant Ultraviolet 737 anti-*mouse* CD45 (30-F11, 1:1600, BD Biosciences Cat 748371, Lot 169519), Brilliant Violet 605 anti-*mouse* CD90.2 (53-2.1, 1:1000, Biolegend Cat 140318, Lot B264059), PE-Cyanine7 anti-*mouse* KLRG1 (2F1/KLRG1, 1:200, Biolegend Cat 138416, Lot B323077), PE anti-*mouse* ST2 (RMST2-2, 1:100, Invitrogen Cat 12-9335-82, Lot 4324692), Brilliant Violet 711 anti-*mouse* NK1.1 (PK136, 1:200, Biolegend, Cat 108745, Lot B267734) and Brilliant Violet 650 NKp46 (29A1.A, 1:50, Biolegend Cat 137635, Lot B374401).

### Isolation of murine bladder mast and tumor cells

*Murine* bladder cancer cells and mast cells were isolated from *mouse* bladders. Prior to cancer cell instillation, MB49 cells were stained with 5 μM CellTrace™ Violet (CTV) dye (Life Technologies) for 20 min at 37 °C. At sacrifice, bladders were cut in pieces and digested with Liberase TM (Roche) and DNAse I (Invitrogen) at 37 °C for 20 min. Red blood cells were lysed using a lysis buffer (Qiagen). The resulting cell suspension was filtered using a 100 µm strainer and then stained for cell sorting. Cancer cells were identified as CD45-CTV^+^ and mast cells as CD45^+^FcεRIα^+^CD117^+^. Cells were sorted using a FACS Aria II (BD Biosciences). The markers used include: Celltrace^TM^ Violet (Life Technologies) Alexa Fluor 647 anti-*mouse* FcεRIα (MAR-1, 1:200, Biolegend, Cat 134310, Lot B434819), Brilliant Violet 421 anti-*mouse* CD117 (2B8, 1:400. Biolegend Cat 105828, Lot B361614) and Brilliant Ultraviolet 737 anti-*mouse* CD45 (30-F11, 1:1600, BD Biosciences Cat 748371, Lot 169519).

### Flow Cytometry analysis on mouse immune cells

Additional markers used include Alexa Fluor 647 anti-*mouse* FcεRIα (MAR-1, 1:200, Biolegend Cat 134310, Lot B434819), Brilliant Violet 421 anti-*mouse* CD117 (2B8, 1:400, Biolegend Cat 105828, Lot B361614), Alexa 700 anti-*mouse* CD11b (M1/70, 1:200 Biolegend Cat 101222, Lot B428765), Brilliant Ultraviolet (BUV737) anti-*mouse* CD45 (30-F11, 1:1600, BD Biosciences Cat 748371, Lot 169519), FITC anti-*mouse* CD3 (REA641, 1:800, Miltenyi Cat 130-119-798, Lot 5191212768), AlexaFluor700 anti-*mouse* CD3 (500A2, 1:400, BD Pharmingen, Cat 557964, Lot 2060194), FITC anti-*mouse* Ly6C (HK1.4, 1.1600, Biolegend Cat 128022, Lot B290041), PE Dazzle anti-*mouse* Ly6G (1A8, 1:200, Biolegend Cat 127647, Lot B337525), Brilliant Violet 421 anti-*mouse* Siglec-F (E502440, 1:200, BD Biosciences Cat 565934, Lot 1138052), Brilliant Violet 711 anti-*mouse* CD11c (N418, 1:200, Biolegend Cat 117349, Lot B265348), PE-Cyanine7 anti-*mouse* CD11c (N418, 1:800, Biolegend Cat 117317, Lot B392337), PE-Cyanine7 anti-*mouse* CD25 (PC61.5, 1:200, Biolegend Cat 101915, Lot B383863), PerCP anti-*mouse* CD25 (PC61, 1:200, Biolegend Cat 102027, Lot B378944), APC-eFluor 780 anti-*mouse* F4/80 (BMB, 1:100, Biolegend Cat 157315 Lot B389904), Brilliant Violet 605 anti-*mouse* CD4 (RM4-5, 1:200, Biolegend Cat 116027, Lot B415523), Spark Blue550 anti-*mouse* CD4 (L3T4, 1:200 Biolegend Cat 100473 Lot B417887), Brilliant Violet 650 anti-*mouse* CD8 (53-6.7, 1:100 Biolegend Cat 100788, Lot B354035), Spark Yellow Green 570 anti-*mouse* CD8 (53-6.7, 1:200 Biolegend, Cat 100788, Lot B354035), Brilliant Violet 750 anti-*mouse* CD44 (IM7, 1:400, Biolegend Cat 103079, Lot B394087), Brilliant Violet 711 anti-*mouse* NK1.1 (PK136, 1:200, Biolegend Cat 108745, Lot B267734), Brilliant Violet 650 anti-*mouse* TCRγδ (GL3, 1:100, Biolegend Cat 118147, Lot B407692), Spark UV387 anti-*mouse* CD90.2 (S2008D, 1:11600, Biolegend Cat 164803, Lot B420409), PE-Fire 810 anti-*mouse* CD62L (W18021D, 1:400, Biolegend Cat 161205, Lot B413505), PE-Cyanine7 anti-*mouse* Ter119 (TER-119, 1:400, Biolegend Cat 116221, Lot B374823), PE-Cyanine7 anti-*mouse* CD19 (1D3/CD19, 1:800, Biolegend Cat 152417, Lot B389427), PE-Cyanine7 anti-*human* CD5 (53-7.3, 1:1600. Biolegend Cat 100621, Lot B402406) and Zombie UV Fixable Viability kit (1:4000, Biolegend Cat 423108, Lot B407371).

Intracellular staining was performed after fixation and permeabilization of the cells with the FoxP3 Transcription Factor Staining Buffer Set (00-5523-00, Invitrogen) using Alexa Fluor 647 anti-*mouse* FoxP3 (MF-14, 1:100, Biolegend Cat 126407, Lot B385770), PE-Cy5 anti-*mouse* FoxP3 (FJK-16S, 1:100, Life Technologies Cat 15-5773-82, Lot 2408301), PerCP-Cy5.5 anti-*mouse* GATA3 (TWAJ, 1:100, Invitrogen Cat 46-9966-41, Lot 2114184), PerCPeFluor710 anti-*mouse* GATA3 (TWAJ, 1:100, Life Technologies Cat, Lot), PE-efluor 610 anti-*mouse* RORγt (B2D, 1:200, Invitrogen Cat 61698182, Lot 2018435), APCeFluor780 anti-*mouse* IFNγ (XMG1.2, 1:300, Invitrogen Cat 47-7311-82, Lot 2071355) and PE-eFluor610 anti-*mouse* IL-13 (eBio13A, 1:100, Life Technologies Cat 4311635, Lot 61-7133-82).

Samples were acquired on LSR Fortessa Flow Cytometer (BD Biosciences) and on a Cytek Aurora spectral flow cytometer (Cytek Biosciences) and data were analyzed using FlowJo (v. 10.7.1).

### Immunofluorescence of mouse bladder sections

Upon incision, the innermost layer of the urinary bladder was fixed with 4% PFA and stored for structural conservation between two cover slips at room temperature for 30 min. After washing with PBS, the sections were incubated in blocking buffer (PBS + 20% normal goat serum + 0.5% Triton X-100) at room temperature for 2 hours. The staining of CD8^+^ T cells and endothelial cells within the urinary bladder was carried out by incubating a mixture of FITC-conjugated anti-*mouse* CD45 (30-F11, Biolegend), AF594-conjugated anti-*mouse* CD31 (MEC13.3; Biolegend), Alexa Fluor 647-conjugated anti-*mouse* CD8a conjugated (53-6.7, Biolegend) Brilliant Violet-conjugated anti-*mouse* FoxP3 (MF-14, Biolegend) antibodies diluted 1:100 in PBS containing 2% goat serum at 4 °C overnight. The staining of Treg cells and endothelial cells within the urinary bladder was performed by incubating a mix of FITC-conjugated anti-*mouse* CD4 (GK1.5; Biolegend), AF594-conjugated anti-*mouse* CD31 (MEC13.3; Biolegend), Alexa Fluor 647-conjugated anti-*mouse* FoxP3 (MF-14, Biolegend) antibodies diluted 1:100 in PBS containing 2% goat serum at 4 °C overnight. Upon nuclear staining with DAPI (Sigma) the samples were mounted in fluoromount aqueous mounting medium (Sigma-Aldrich). The infiltration of Tregs (CD4^+^/FoxP3^+^) into *murine* urinary bladders after tumor injection was analyzed as follows. The entire opened urinary bladder samples were imaged using an upright spinning disk confocal microscope (Axio Examiner Z1 Advanced Microscope Base, Zeiss) equipped with a confocal scanner unit CSU-X1 A1 (Yokogawa Electric Corporation). The fluorochrome excitation was conducted via four lasers with wavelengths of 405, 488, 561, and 640 nm (LaserStack v4 Base, 3i). Fluorescence was detected using a 10×/0.3 numerical aperture (NA) water immersion objective (W Plan Apochromat, Zeiss), an appropriate bandpass emission filter (Semrock), and an electron-multiplying charge-coupled device camera (Evolve 512 10 MHz Back Illuminated, Photometrics). Three-dimensional image stacks were obtained by sequential acquisition of multiple field of views (FOVs) covering the entire urinary bladder area along the z axis to screen through the tissue thickness using a motorized XY stage (ProScan, Prior). SlideBook software (v. 6.0.17, 3i) was used for image acquisition and the creation of maximum projections. The subsequent generation of montage images from contiguous positions was performed using the Fiji ‘Grid/collection’ stitching plugin^92^. Before analysis, all images were processed using a ‘rolling ball’ algorithm implemented into the Fiji plugin ‘Subtract Background’ to correct for uneven illuminated background^93^. The distribution of Tregs with the urinary bladder was automatically quantified across the entire organ using ImageJ’s (National Institutes of Health) implemented ‘Analyze Particles’ tool upon prior intensity-based thresholding and image segmentation of individual fluorescent channels and was calculated as the ratio of the total CD4^+^/Foxp3^+^-positive area detected to the total area of the urinary bladder imaged.

### NGF quantification in mouse samples

The concentration of *mouse* NGF in bladder tissues was determined using the *mouse* beta-NGF ELISA Kit (Invitrogen), according to the manufacturer’s protocol. Results were normalized using the protein concentration that was previously quantified using Pierce™ BCA Protein Assay Kit (Invitrogen).

### Statistical analysis

Statistical analysis was performed using GraphPad Prism software (v. 10.0.0.). Normality was first assessed with the Shapiro–Wilk test, then adequate statistical analysis was performed based on the dataset. Data are shown by plotting individual data points and the mean ± SEM. A *p*-value less than 0.05 was considered as statistically significant.

### Reporting summary

Further information on research design is available in the Nature Portfolio Reporting Summary linked to this article.

## Supplementary information

Supplementary Information

Reporting Summary

Transparent Peer Review file

## Source data

Source data

## Data Availability

The RNAseq data of *human* ILC2s generated in this study have been deposited in NCBI’s Gene Expression Omnibus database under the GEO accession number GSE311046. The TCGA Bladder Urothelial Carcinoma (BLCA) RNA sequencing dataset is available from the National Institutes of Health’s (NIH) dbGaP (Database of Genotypes and Phenotypes) database under accession number phs000178 [https://portal.gdc.cancer.gov/projects/TCGA-BLCA]. Microarray gene expression datasets are available from the National Center for Biotechnology Information (NCBI)‘s GEO database under accession numbers GSE31684 and GSE48075. - Human ILCs: ArrayExpress accession E-MTAB-8494 (bulk RNA sequencing data) [https://www.ebi.ac.uk/biostudies/ArrayExpress/studies/E-MTAB-8494], GSE112591 (bulk RNA sequencing data) [https://www.ncbi.nlm.nih.gov/geo/query/acc.cgi?acc=GSE112591], GSE150050 (single-cell RNA sequencing, Smart-Seq2 protocol) [https://www.ncbi.nlm.nih.gov/geo/query/acc.cgi?acc=GSE150050] The remaining data are available within the article, Supplementary information or Source Data file and/or from the corresponding author upon request. Source data are provided with this paper.
