# Supplementary material for: Mast-cell derived nerve growth factor drives ILC2 pro-tumoral functions in bladder cancer
Source: Nat Commun. 2026 Feb 21;17:3061. doi: 10.1038/s41467-026-69841-y (PMC13039139; doi:10.1038/s41467-026-69841-y)
Supplement: Supplementary file 1 — Supplementary Information [file 41467_2026_69841_MOESM1_ESM.pdf]

Supplementary Figure 1

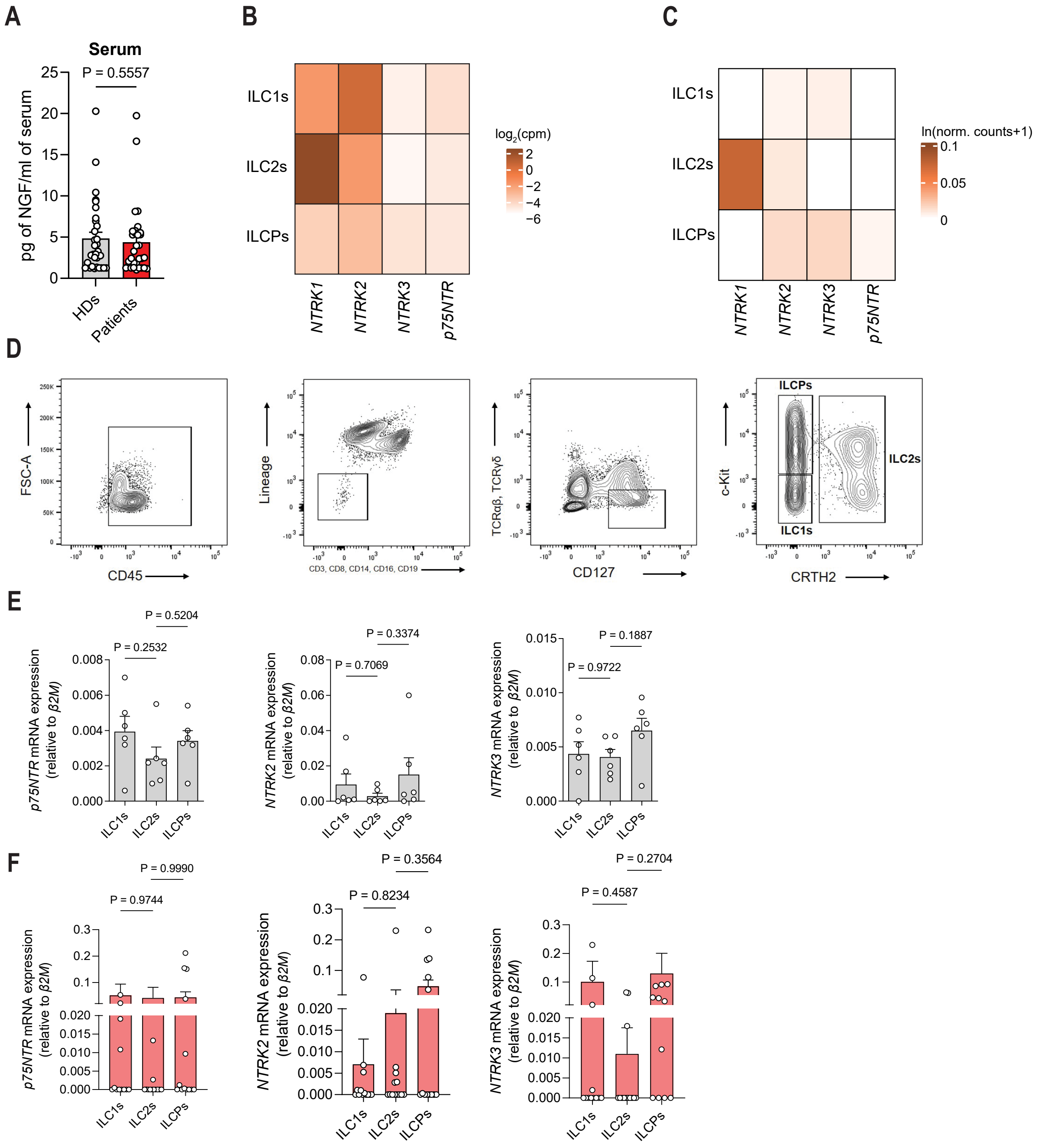

(A) NGF quantification in the serum of healthy donors (HDs, n=31) and BC patients (Patients, n=31).
(B) Heatmap generated using publicly available RNAseq (GSE112591) from healthy human peripheral ILCs representing the neurotrophic receptors' expression. The value of each gene is the average expression from 6 donors.
(C) Heatmap generated using publicly available single-cells RNAseq (GSE150050) from healthy human peripheral blood ILCs representing the neurotrophic receptors' expression. The value of each gene is the average expression of the single cells from 3 donors.
(D) Representative example of the gating strategy used to sort human ILC subsets from peripheral blood. Total ILCs were identified as live $CD45^{+}lineage^{-}CD127^{+}$ lymphocytes, after doublets exclusion. Within total ILCs, ILC1s were defined as $CRTH2^{-}c\text{-}Kit^{-}$, ILC2s as $CRTH2^{+}c\text{-}Kit^{+/-}$ and ILCPs as $CRTH2^{-}c\text{-}Kit^{+}$.
(E) Relative expression of *p57NTR, NTRK2 and NTRK3* in *ex vivo* ILC subsets from HDs (n=6).
(F) Relative expression of *p75NTR*, *NTRK2* and *NTRK3* in *ex vivo* sorted ILC subsets from BC patients (n=10).
Data are represented as the mean ± SEM and pooled from one to three independent experiments. P values were determined using two-tailed Mann Whitney test (A) and by Dunnett's multiple comparisons test (E, F).
Source data are provided as a Source Data file.

Supplementary Figure 2

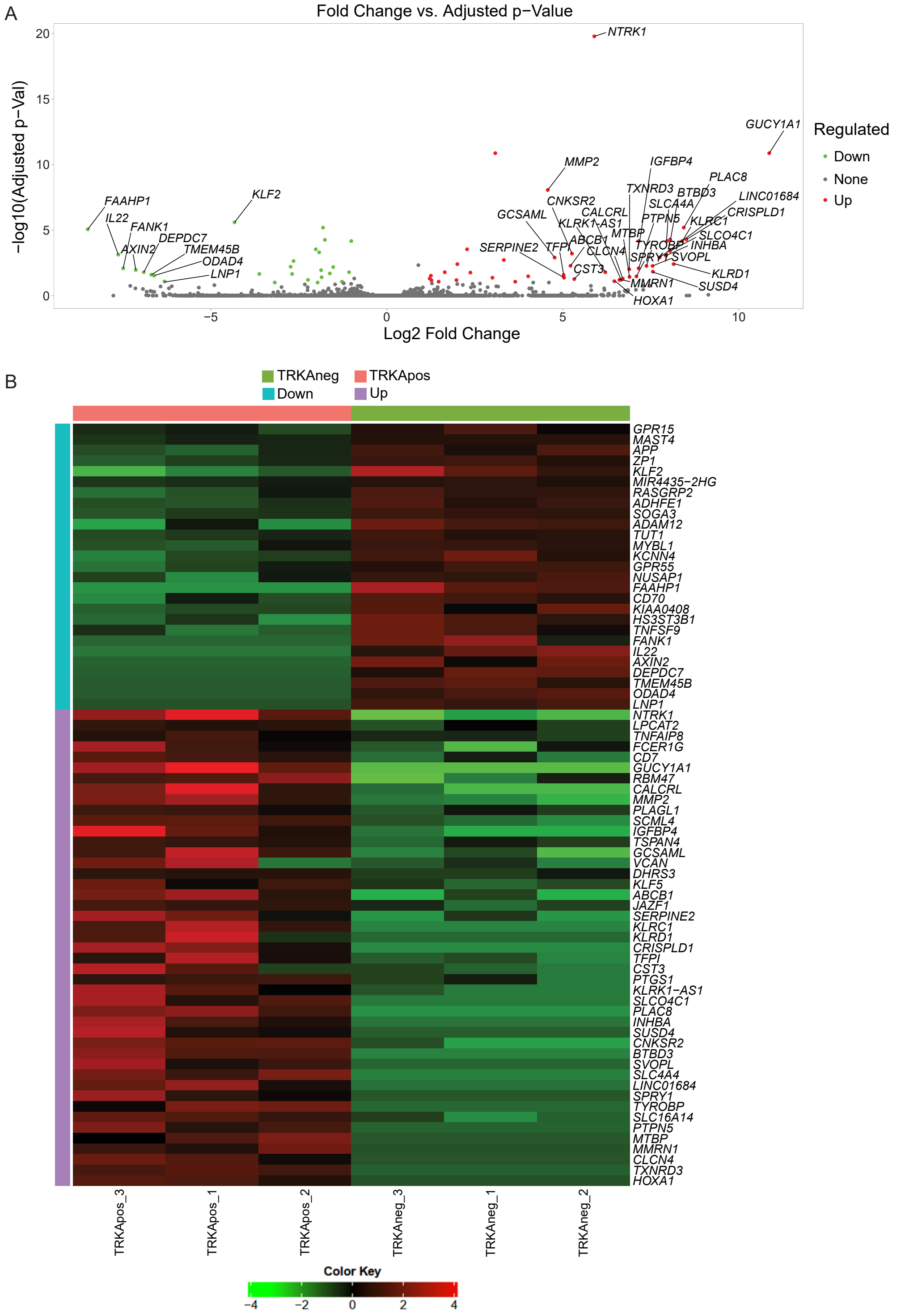

(A) Gene expression volcano plot visualizing the results of the differential expression analysis comparing TrkA^{+} (n=3) and TrkA^{-} (n=3) ILC2s. Top differentially expressed 50 genes were labelled. X-axis represents the log2 fold change in expression between the two groups. Positive values (red dots) indicate increased expression in TrkA^{+} ILC2s, while negative values (green dots) indicate decreased expression in TrkA^{+} ILC2s. Y-axis represents the statistical significance of the differential expression, typically expressed as the negative base-10 logarithm of the adjusted p-value (-log10(adjusted p-value). Higher values on the y-axis indicate more statistically significant differences in expression.

(B) Heatmap visualizing the differentially expressed genes (DEGs) (FC >2, padj < 0.1) between $TrkA^{+}$(n=3) and $TrkA^{-}$ (n=3) ILC2s. Green represents lower expression levels, while red represents higher expression levels. The color intensity indicates the magnitude of gene expression.
Source data are provided as a Source Data file.

Supplementary Figure 3

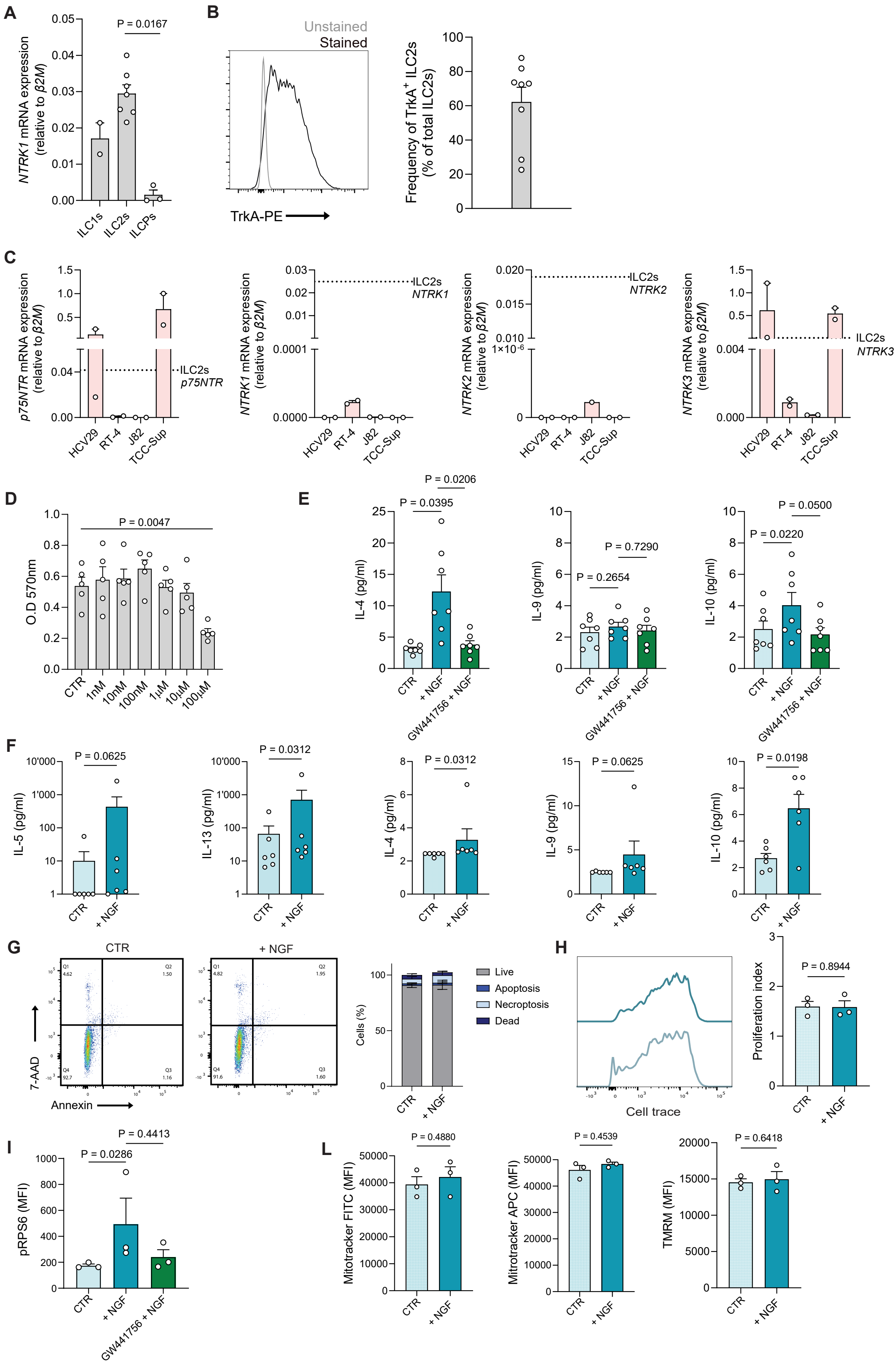

(A) Relative expression of *NTRK1* in expanded ILC subsets from HDs (ILC1s: n=2; ILC2s: n=7; ILCPs: n=3).
(B) Representative histogram (left panel) and quantification (right panel) of TrkA expression by expanded ILC2s (n=8).
(C) Relative expression of *p75NTR, NTRK1*, *NTRK2* and *NTRK3* in human healthy bladder and bladder cancer cell lines (n=2/cell line).
(D) MTT assay of short-term *in vitro* expanded ILC2s treated with a range of different concentrations of GW441756 for 48 hours (n=5).
(E) IL-4, IL-9 and IL-10 secretion quantification by short-term *in vitro* expanded ILC2s from HDs unstimulated (CTR) and stimulated with NGF for 48 hours (+ NGF) (n=7).
(F) IL-5, IL-13, IL-4, IL-9 and IL-10 secretion quantification by *ex vivo* sorted ILC2s from HDs unstimulated (CTR) and stimulated with NGF for 48 hours (+ NGF) (n=6).
(G) Representative dot plots of 7-AAD/Annexin V staining performed on short-term expanded ILC2s from HDs unstimulated (CTR) or stimulated with NGF for 48 hours (+ NGF) (left panel) and quantification (right panel) (n=3).
(H) Representative histogram of cell proliferation assay performed on short-term *in vitro* expanded ILC2s from HDs upon NGF stimulation for 5 days (left panel) and quantification (right panel) (n=3).
(I) Quantification of MFI of phosphorylated S6 (pS6) of short-term *in vitro* expanded ILC2s unstimulated (CTR), stimulated with NGF for 3 hours, and pre-treated or not with GW441756 for 45 minutes prior NGF stimulation (GW441756 + NGF) (n=3).
(L) Quantification of mitochondrial mass (Mitotracker) and mitochondrial potential (Mitotracker Deep red and TMRM) changes in short-term expanded ILC2s unstimulated (CTR) and stimulated with NGF for 48 hours (+ NGF) (n=3).
Data are represented as the mean ± SEM and pooled from two to three independent experiments. P values were determined by Dunnett's multiple comparisons test (A, D), Sidak’s multiple comparisons (E, I), two-tailed Wilcoxon matched-pairs signed rank test (F, H, L).
Source data are provided as a Source Data file.

Supplementary Figure 4

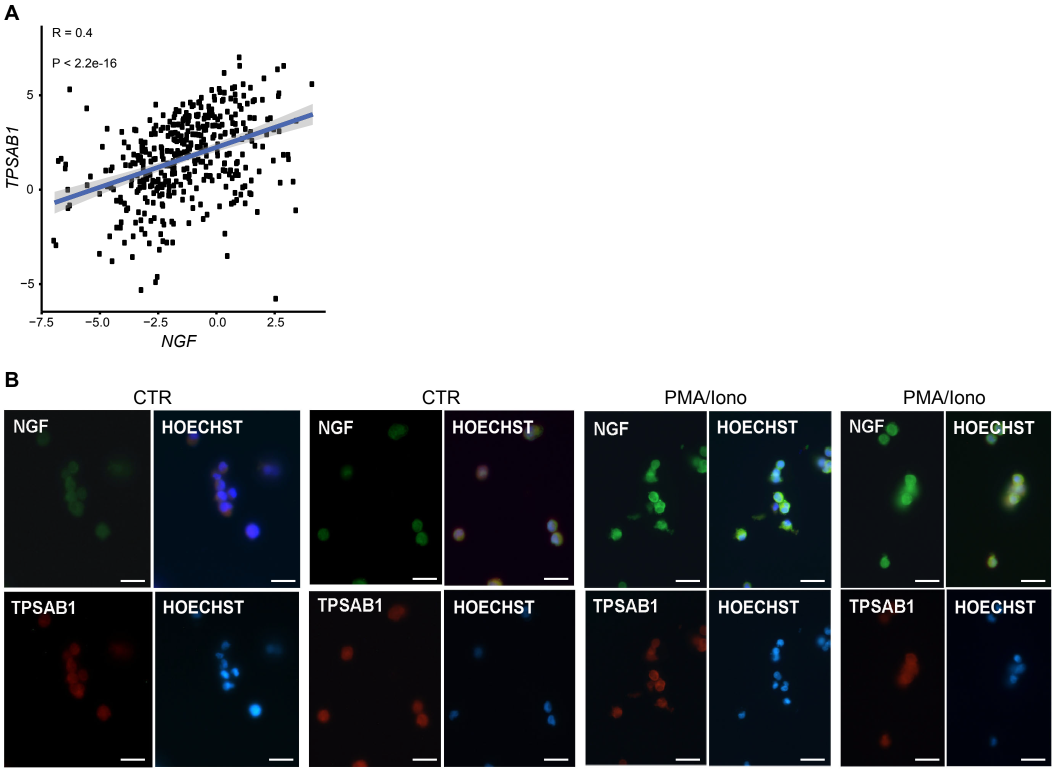

(A) Pearson's correlation between *NGF* and *TPSAB1* expression (in log_2[counts per million]) in tumors of bladder cancer patients of the TCGA BLCA cohort (n=405).
(B) HMC1 cells were analyzed by immunostaining for NGF (green) and tryptase (TPSAB1) (red) in absence (CTR) or presence (PM/Iono) treatment for 6 hours. Nuclei are labeled with Hoechst in blue (scale bar = 10 µm)

Supplementary Figure 5

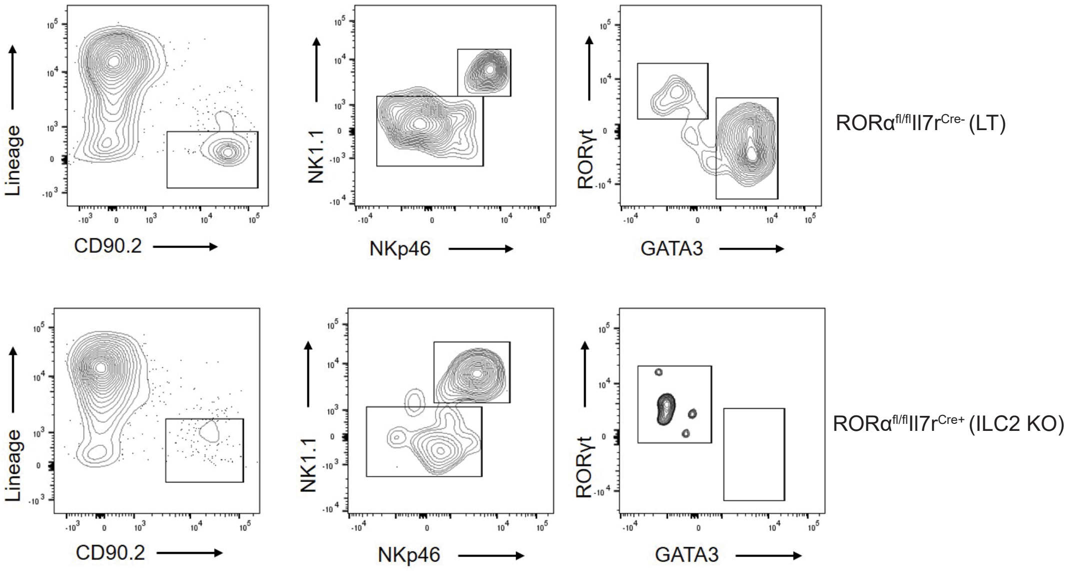

Representative example of ILC subsets' gating strategy in bladder from RORα$^{fl/fl}$Il7r^{Cre-} (LT) (upper panel) and RORα$^{fl/fl}$Il7r^{Cre+} (KO) mice (lower panel).

Supplementary Figure 6

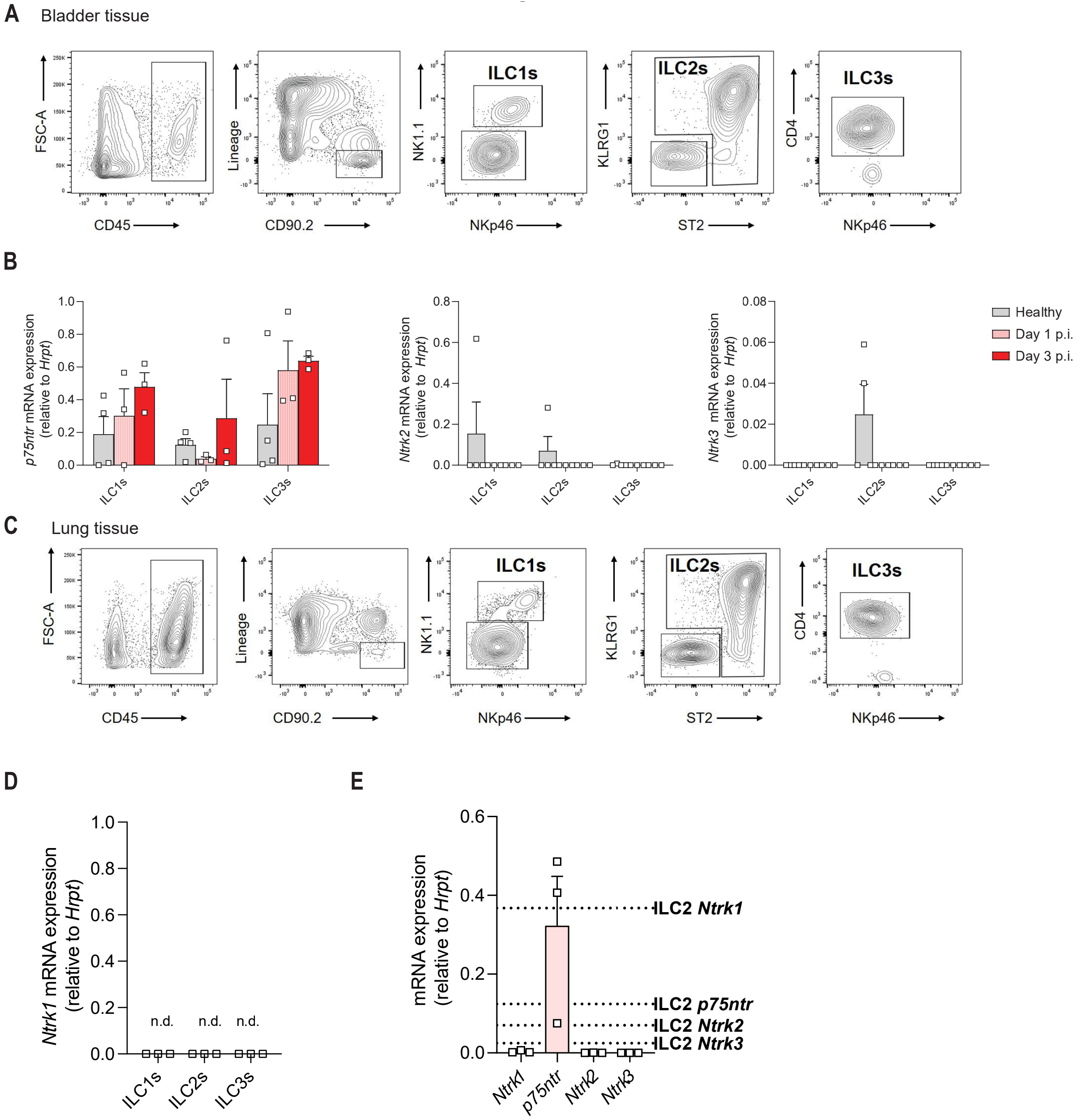

(A) Representative example of the gating strategy used to sort mouse bladder ILC subsets.
(B) Relative expression of *p75ntr*, *Ntrk2* and *Ntrk3* in *ex vivo* sorted ILC subsets from healthy bladder (n=4) and tumor-infiltrated bladders at day 1 (Day 1 p.i., n=3) and day 3 (Day 3 p.i., n=3) post-MB49 instillation, determined by qPCR.
(C) Representative example of the gating strategy used to sort mouse lung ILC subsets.
(D) Relative expression of *Ntrk1* by lung ILC2s (n=3).
(E) Relative expression of *p75ntr, Ntrk1*, *Ntrk2* and *Ntrk3* by MB49 mouse bladder tumor cell line (n=3). Dashed lines indicate the relative expression levels of the genes in murine ILC2s (mean value extracted from Figures 3F and S6B).
Data are represented as the mean ± SEM and pooled from one to three independent experiments.
Source data are provided as a Source Data file.

Supplementary Figure 7

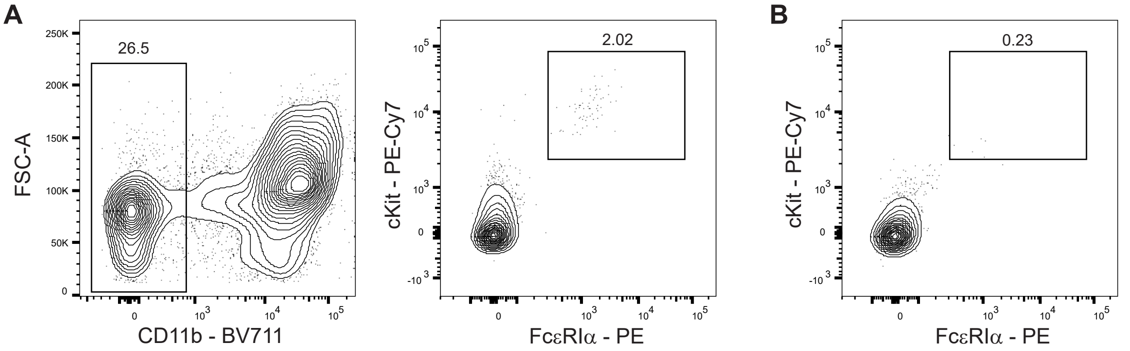

(A) Representative example of the gating strategy used to sort mouse bladder mast cells.
(B) Representative example of bladder mast cell staining in anti-cKit depleted animals (n=5).

Supplementary Figure 8

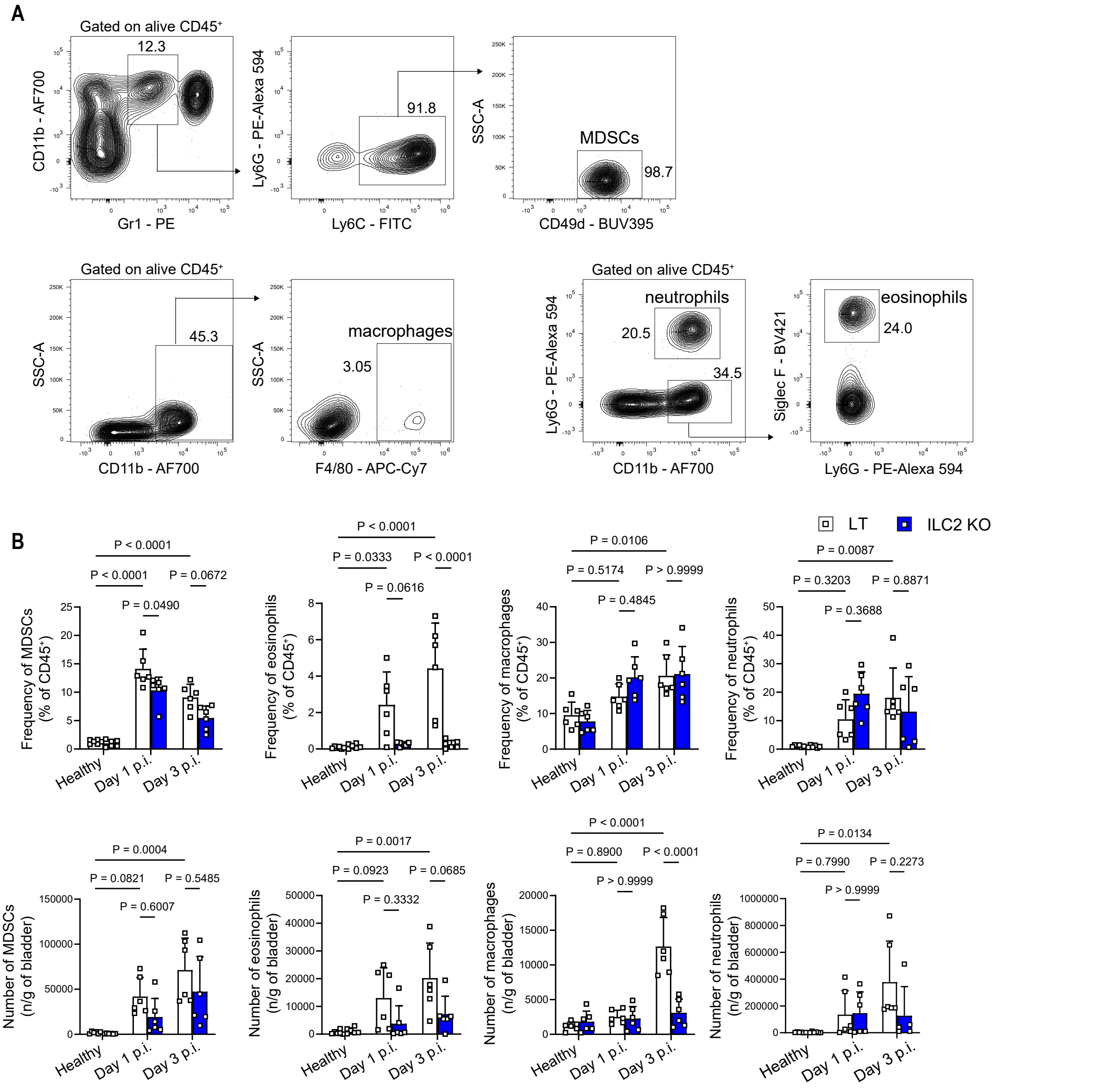

(A) Representative example of the gating strategy used to identify bladder myeloid cell populations.
(B) Frequency (upper graphs) and absolute numbers (lower graphs) of monocytic myeloid-derived suppressors cells (M-MDSCs), eosinophils, macrophages and neutrophils in healthy (n=6) and tumor bearing RORα$^{fl/fl}$Il7r^{Cre-} (LT) and RORα$^{fl/fl}$Il7r^{Cre+} (ILC2 KO) mice at day 1 (Day 1 p.i., n=6) and day 3 (Day 3 p.i., n=6) post-MB49 instillation, determined by flow cytometry.
Data are represented as the mean ± SEM and pooled from two independent experiments. P values were determined by two-way ANOVA with Tukey's multiple comparisons test (B).
Source data are provided as a Source Data file.

Supplementary Figure 9

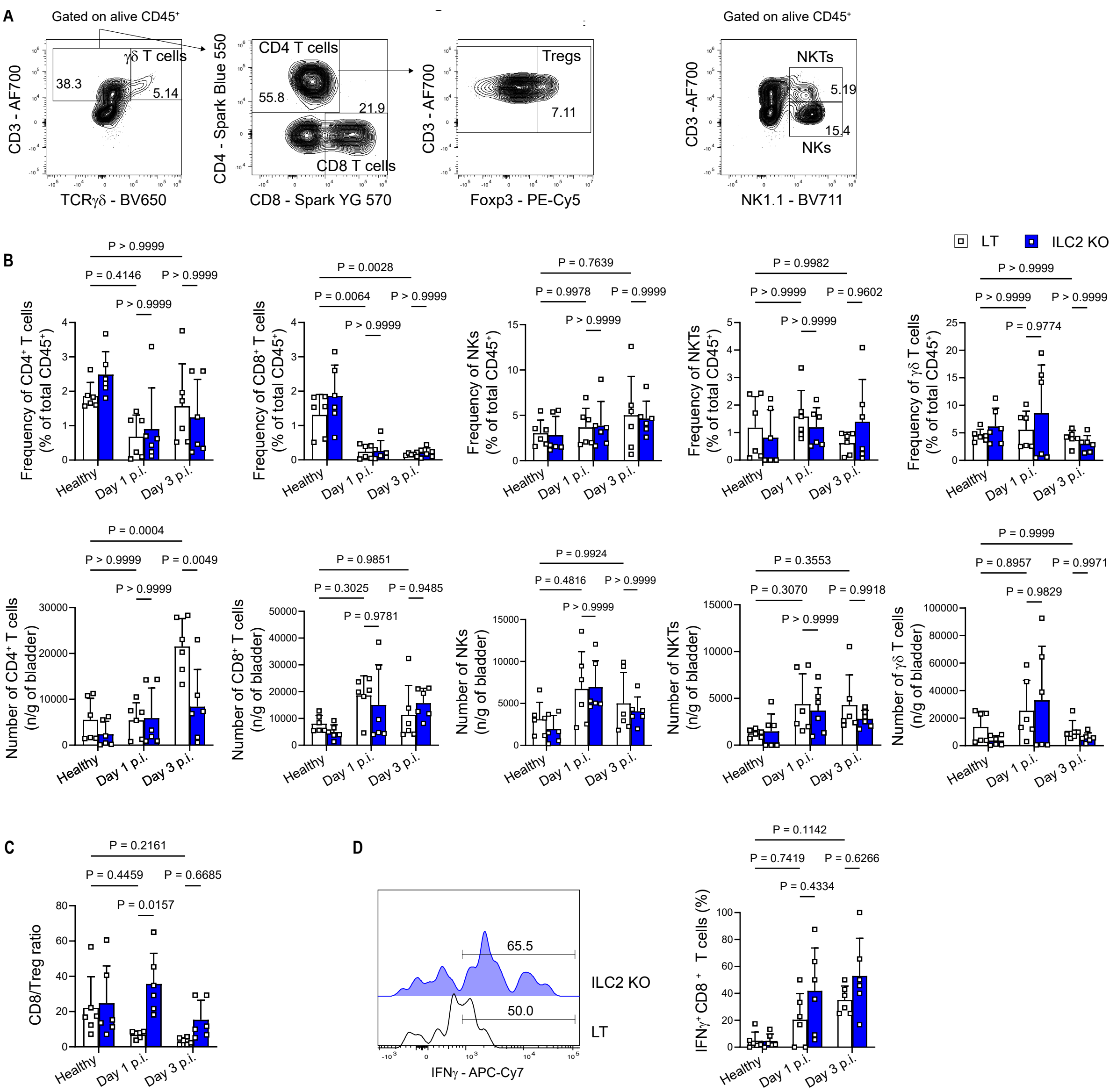

(A) Representative example of the gating strategy used to identify bladder lymphocytic cell populations.
(B) Frequency (upper graphs) and absolute numbers (lower graphs) of CD4+ T cells, CD8+ T cells, NKs, NKTs and γδ T cells in healthy (n=6) and tumor bearing RORα$^{fl/fl}$Il7r^{Cre-} (LT) and RORα$^{fl/fl}$Il7r^{Cre+} (ILC2 KO) mice at day 1 (Day 1 p.i., n=6) and day 3 (Day 3 p.i., n=6) post-MB49 instillation, determined by flow cytometry.
(C) CD8 T cell/Treg ratio in healthy (n=6) and tumor bearing RORα$^{fl/fl}$Il7r^{Cre-} (LT) and RORα$^{fl/fl}$Il7r^{Cre+} (ILC2 KO) mice at day 1 (Day 1 p.i., n=6) and day 3 (Day 3 p.i., n=6) post-MB49 instillation, determined by flow cytometry.
(D) Representative histograms (left panel) and quantification (right panel) of IFNγ+ CD8+ T cells in healthy (n=6) and tumor bearing RORα$^{fl/fl}$Il7r^{Cre-} (LT) and RORα$^{fl/fl}$Il7r^{Cre+} (ILC2 KO) mice at day 1 (Day 1 p.i., n=6) and day 3 (Day 3 p.i., n=6) post-MB49 instillation, determined by flow cytometry.
Data are represented as the mean ± SEM and pooled from two independent experiments. P values were determined by two-way ANOVA with Tukey's multiple comparisons test (B, C, D).
Source data are provided as a Source Data file.

Supplementary Figure 10

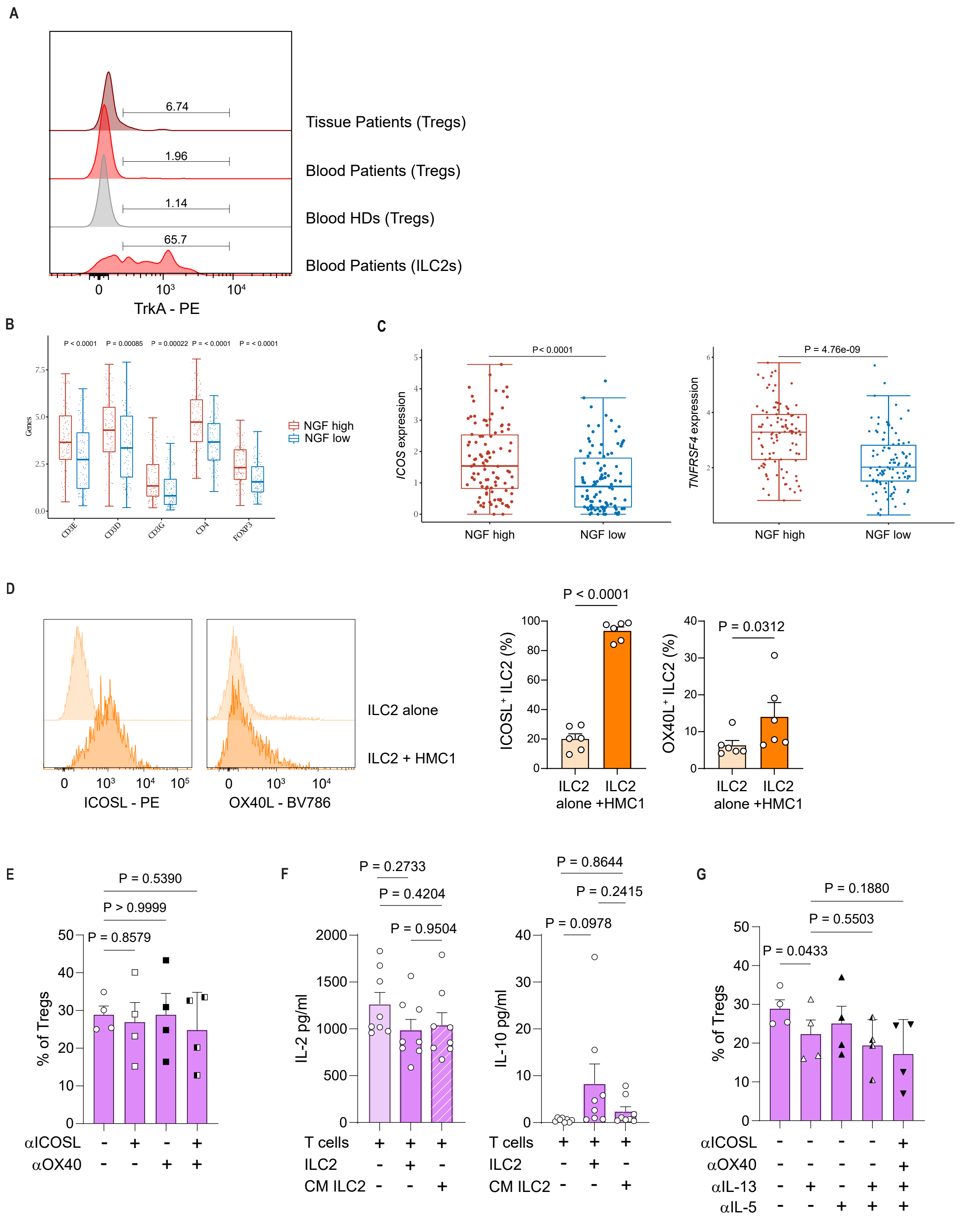

(A) Representative histograms of TrkA expression by *ex vivo* Tregs from healthy donors' (HDs) and bladder cancer patients' blood (Blood Patients) and tumor tissue (Tissue Patients). TrkA expression by *ex vivo* ILC2s is shown as comparison. Representative histograms of n=8 independent biological samples, per sample type.

(B) Treg-associated genes in tumors of TCGA BC patients stratified into high and low *NGF* groups (n=102 per group). The x-axis represents different sample groups, and the y-axis represents the distribution of gene expression, which is normalized using log2(TPM+1).

(C) *ICOS* (left panel) and *TNFRSF4* (*OX40)* expression in tumors of TCGA BC patients stratified into high and low *NGF* groups (n=102 per group). The y-axis represents the distribution of gene expression, which is normalized using log2(TPM+1).
(D) Representative histograms (left) and quantification (right) of ICOSL and OX40L expression by human short-term *in vitro* expanded ILC2s alone or upon 48-hour co-culture with the HMC1 mast cell line (n=6).
(E) Frequency of Tregs induced by the 6-day co-culture of human naïve $CD4^+$ T cells with autologous short-term *in vitro* expanded ILC2s, in the presence of the indicated blocking antibodies, quantified by flow cytometry (n=4).
(F) IL-2 and IL-10 quantification in the supernatants of 6-day co-cultures of human naive $CD4^+$ T cells with autologous short-term expanded ILC2s or CM (n=8).
(G) Frequency of Tregs induced by the 6-day co-culture of human naïve $CD4^+$ T cells with autologous short-term *in vitro* expanded ILC2s, in the presence of the indicated blocking antibodies, quantified by flow cytometry (n=4).
Data in D-G are represented as the mean ± SEM and pooled from three independent experiments. P values were determined by Wilcoxon Rank sum test for each gene (B, C), paired t-test and two-tailed Wilcoxon matched-pairs signed rank test (D, left and right graphs, respectively), one-way Anova with Geisser-Greenhouse correction and Dunnett's multiple comparisons test (E, G) and Tukey's multiple comparisons test (F).
Source data are provided as a Source Data file.

Supplementary Figure 11

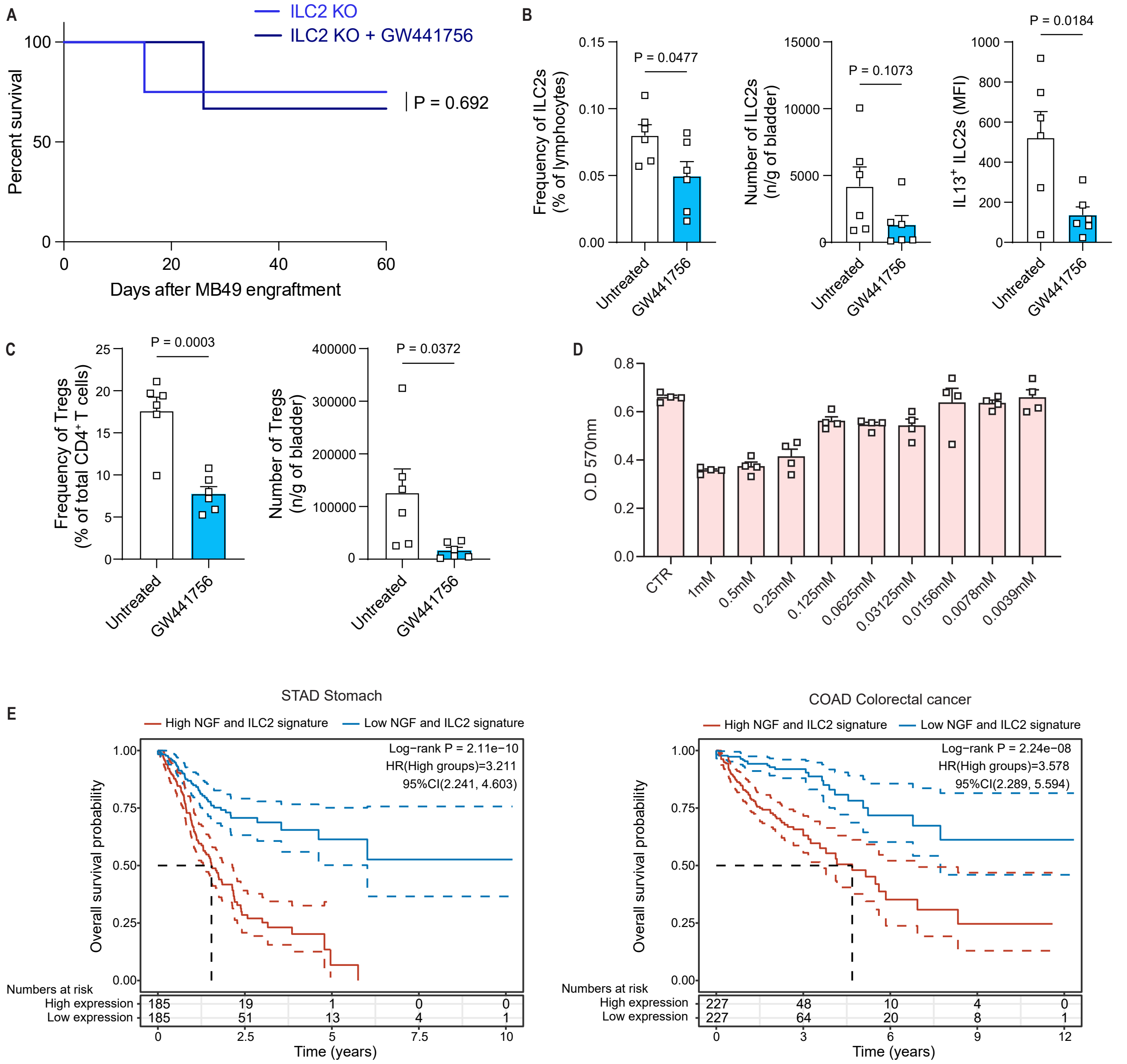

(A) Survival curve of MB49 tumor-bearing Rorα$^{f/f}$Il7r^{Cre+} (ILC2 KO) mice, treated (n=12) or not with GW441756 (n=12).
(B) Frequency (left graph) and absolute numbers (middle graph) of ILC2s, and IL-13 MFI in ILC2s (right graph) in untreated (n=6) and GW441756 treated (n=6) WT animals, 10 days post-MB49 engraftment.
(C) Frequency (left graph) and absolute numbers (right graph) of Tregs in untreated (n=6) and GW441756 treated (n=6) WT animals, 10 days post-MB49 engraftment.
(D) MTT assay on the MB49 mouse bladder tumor cell line treated with a range of different concentrations of GW441756 for 48 hours (n=4).
(E) Survival analysis performed on the impact of *NGF* expression and ILC2 infiltration in gastric (STAD Stomach) and colon adenocarcinoma (COAD Colorectal cancer) patients, based on TCGA Dataset.
Data are represented as the mean ± SEM and pooled from two experiment. P values were determined by Log-rank (Mantel-Cox) test (A) and Mann-Whitney U test (B, C).
Source data are provided as a Source Data file.

Supplementary Table 1. Clinical characteristics of the BC patients.

Parameter	Total cases	Percentage %
**Sexe**		
Female	21	29,58
Male	50	70,42
**Age**		
<50	3	4,23
50-70	36	50,70
>70	32	45,07
**Tumor stage**		
≤ PT1	55	77,46
>PT1	16	22,54
**Tumor grade**		
Low grade	29	40.85
High grade	41	57.75

[a]Calculated at the surgery date.
