# Supplementary material for: Mast-cell derived nerve growth factor drives ILC2 pro-tumoral functions in bladder cancer
Source: Nat Commun. 2026 Feb 21;17:3061. doi: 10.1038/s41467-026-69841-y (PMC13039139; doi:10.1038/s41467-026-69841-y)
Supplement: Supplementary file 2 — Reporting Summary [file 41467_2026_69841_MOESM2_ESM.pdf]

nature portfolio

Corresponding author(s): Camilla Jandus

Last updated by author(s): Jan 23, 2026

Reporting Summary

Nature Portfolio wishes to improve the reproducibility of the work that we publish. This form provides structure for consistency and transparency in reporting. For further information on Nature Portfolio policies, see our Editorial Policies and the Editorial Policy Checklist.

Statistics

For all statistical analyses, confirm that the following items are present in the figure legend, table legend, main text, or Methods section.

n/a	Confirmed	
[ ]	[x]	The exact sample size (*n*) for each experimental group/condition, given as a discrete number and unit of measurement
[ ]	[x]	A statement on whether measurements were taken from distinct samples or whether the same sample was measured repeatedly
[ ]	[x]	The statistical test(s) used AND whether they are one- or two-sided<br>*Only common tests should be described solely by name; describe more complex techniques in the Methods section.*
[ ]	[x]	A description of all covariates tested
[ ]	[x]	A description of any assumptions or corrections, such as tests of normality and adjustment for multiple comparisons
[ ]	[x]	A full description of the statistical parameters including central tendency (e.g. means) or other basic estimates (e.g. regression coefficient) AND variation (e.g. standard deviation) or associated estimates of uncertainty (e.g. confidence intervals)
[ ]	[x]	For null hypothesis testing, the test statistic (e.g. *F*, *t*, *r*) with confidence intervals, effect sizes, degrees of freedom and *P* value noted<br>*Give P values as exact values whenever suitable.*
[x]	[ ]	For Bayesian analysis, information on the choice of priors and Markov chain Monte Carlo settings
[x]	[ ]	For hierarchical and complex designs, identification of the appropriate level for tests and full reporting of outcomes
[ ]	[x]	Estimates of effect sizes (e.g. Cohen's *d*, Pearson's *r*), indicating how they were calculated

Our web collection on statistics for biologists contains articles on many of the points above.

Software and code

Policy information about availability of computer code

Data collection

Cell isolation: FACS Aria II (BD Biosciences)
Flow cytometry: LSR Fortessa Flow Cytometer (BD Biosciences), Cytek Aurora spectral flow cytometer (Cytek Biosciences) and FlowJo (v. 10.7.1).
Quantitative real-time PCR: 7900HT Fast Real-Time PCR Sequence Detection System (Applied Biosystems)
MTT assay: Microplate spectrophotometer (TitertekMultiskan MCC/340), equipped with a 570nm filter
Cytokine and NGF quantification: Attune NxT instrument and LEGENDplex™ Data Analysis software (v. 8.0).
Immunofluorescence and immunohistochemical analysis of TrkA: Images were captured with the Axiocam Fluo microscope (Zeiss).
Bladder cancer patient sections: Images were captured with the Panoramic scan 250 Flash II (3DHistech).
Immunohistochemical colocalization : Images were taken on an IX71 inverted microscope (Olympus, Hamburg, Germany) equipped with a ColorViewII camera (Olympus, Hamburg, Germany). Merged images were assembled using ImageJ.
RNAseq: Libraries were sequenced on a NovaSeq 6000 Illumina sequencer for SR100 reads.

Mouse bladder sections: Imaging with an upright spinning disk confocal microscope (Axio Examiner Z1 Advanced Microscope Base, Zeiss) equipped with a confocal scanner unit CSU-X1 A1 (Yokogawa Electric Corporation). The fluorochrome excitation was conducted via four lasers with wavelengths of 405, 488, 561, and 640 nm (LaserStack v4 Base, 3i). Fluorescence was detected using a 10×/0.3 numerical aperture (NA) water immersion objective (W Plan Apochromat, Zeiss), an appropriate bandpass emission filter (Semrock), and an electron-multiplying charge-coupled device camera (Evolve 512 10 MHz Back Illuminated, Photometrics). Three-dimensional image stacks were obtained by sequential acquisition of multiple fields of views (FOVs) along the z-axis to screen through the tissue thickness using a motorized XY stage (ProScan, Prior). SlideBook software (v. 6.0.17, 3i) was used for image acquisition and the creation of maximum projections. The subsequent generation of montage images from contiguous positions was performed using the Fiji 'Grid/collection' stitching plugin. Before analysis, all

images were processed using a ‘rolling ball’ algorithm implemented in the Fiji plugin ‘Subtract Background’ to correct for uneven illuminated background. Cell quantification within images was done with ImageJ’s (National Institutes of Health) implemented ‘Analyze Particles’ tool upon prior intensity-based thresholding and image segmentation of individual fluorescent channels.

Publicly available transcriptomics datasets:
- Human bladder cancer tumors: TCGA BLCA (bulk RNA sequencing data), GSE31684, (microarray: Affymetrix Human Genome U133 Plus 2.0 Array), GSE48075 (microarray: Illumina HumanHT-12 V3.0 expression beadchip).
- Human ILCs: ArrayExpress accession E-MTAB-8494 (bulk RNA sequencing data), GSE112591 (bulk RNA sequencing data), GSE150050 (single-cell RNA sequencing, Smart-Seq2 protocol).
-The RNAseq data of human ILC2s generated in this study have been deposited in NCBI's Gene Expression Omnibus database under the GEO accession number GSE311046 (https://www.ncbi.nlm.nih.gov/geo/query/acc.cgi?acc=GSE311046).

Data analysis

Transcriptomics profiles of tumors of 572 MIBC patients with available clinical information were pooled from three datasets, namely TCGA BLCA, GSE31684 and GSE48075. Survival analysis was performed using the coxph function of the survival package (v. 3.2.3) for R (v. 4.2).

Transcriptomics profiles of human ILCs: Raw gene counts of bulk RNA sequencing data were imported into R. Genes expressed at a level of at least 1 count per million (cpm) in at least 1 sample were retained and normalization factors were calculated using the trimmed mean of M values method of the edgeR package (v. 3.24.3). The counts data was subsequently transformed to log2(counts per million) using the voom function of the limma package (v. 3.38.3).
For the single-cell RNAseq dataset, the raw counts of peripheral blood ILCs were converted to ln(normalized counts+1) using the Seurat package (v.4.0.3) for R.
Heatmaps were drawn using the ComplexHeatmap package (v. 1.20.0).

Correlation of gene expression within bladder cancer tumors: Raw gene counts of bulk RNA sequencing data of 412 TCGA BLCA tumors were downloaded using the TCGAbiolinks package (v. 2.10.5) for R. Cases were filtered to only retain the ones that derived from primary tumors (sample type 01A) and that had clinical data present, resulting in 405 retained samples. Counts were converted to log2(counts per million) using the edgeR (v. 3.24.3) and limma packages (v. 3.38.3) for R. Pearson’s correlation coefficients among gene expression values per sample was calculated using the cor.test function of the stats (v. 3.5.3) package.

Statistical analysis not performed within R was performed using GraphPad Prism software (v. 10.0.0.). Data normality was assessed with the Shapiro-Wilk test, then adequate statistical analysis was performed based on the dataset.

A p-value less than 0.05 was considered as statistically significant. P-value adjustment using the Benjamini-Hochberg procedure was applied when multiple comparisons were performed.

For manuscripts utilizing custom algorithms or software that are central to the research but not yet described in published literature, software must be made available to editors and reviewers. We strongly encourage code deposition in a community repository (e.g. GitHub). See the Nature Portfolio guidelines for submitting code & software for further information.

Data

Policy information about availability of data

All manuscripts must include a data availability statement. This statement should provide the following information, where applicable:
- Accession codes, unique identifiers, or web links for publicly available datasets
- A description of any restrictions on data availability
- For clinical datasets or third party data, please ensure that the statement adheres to our policy

Links to publicly available data:
Human bladder cancer transcriptomics profiles:
https://portal.gdc.cancer.gov/projects/TCGA-BLCA
https://www.ncbi.nlm.nih.gov/geo/query/acc.cgi?acc=GSE31684
https://www.ncbi.nlm.nih.gov/geo/query/acc.cgi?acc=GSE48075

ILC transcriptomics profiles:
https://www.ebi.ac.uk/biostudies/arrayexpress/studies/E-MTAB-8494
https://www.ncbi.nlm.nih.gov/geo/query/acc.cgi?acc=GSE112591
https://www.ncbi.nlm.nih.gov/geo/query/acc.cgi?acc=GSE150050

The RNAseq data of human ILC2s generated in this study have been deposited in NCBI's Gene Expression Omnibus database under the GEO accession number GSE311046 (https://www.ncbi.nlm.nih.gov/geo/query/acc.cgi?acc=GSE311046).

Sample size/Replication

Human MIBC transcriptomics profiles, number of independent donors:
TCGA-BLCA: n=412
GSE31684: n=93
GSE48075: n=73 (MIBC)

ILC transcriptomics profiles:

E-MTAB-8494: n=3 per ILC subset
GSE112591: n=6 per ILC subset
GSE150050 (scRNAseq, circulating ILCs): 222 ILC1s, 177 ILC2s, 147 ILC3s, obtained from 3 donors

Data exclusions:

For MIBC transcriptomics profiles, we retained samples that classified as muscle-invasive bladder cancer and that had associated patient survival information.
All data are available in the main text or the supplementary materials. Correspondence and material requests should be addressed to camilla.jandus@unige.ch.

Research involving human participants, their data, or biological material

Policy information about studies with human participants or human data. See also policy information about sex, gender (identity/presentation), and sexual orientation and race, ethnicity and racism.

Reporting on sex and gender	Only sex was taken into consideration and was reported to the study nurse at hospital admission. A total of 71 patients participated in the reserach work, 21 female and 50 male (ages: 3 patients younger than 50; 36 patients aged 50-70; 32 patients older than 70).
Reporting on race, ethnicity, or other socially relevant groupings	Race was collected upon admission at the hosptial by the clinical nurse. This parameter was not taken into consideration during data analyses. It can be retrieved upon request.
Population characteristics	N/A
Recruitment	All patients were enrolled after the signature of an informed consent.
Ethics oversight	The study was approved by the local ethical committee (EC 2020-02375; VD431/13)

Note that full information on the approval of the study protocol must also be provided in the manuscript.

Field-specific reporting

Please select the one below that is the best fit for your research. If you are not sure, read the appropriate sections before making your selection.

- [x] Life sciences
- [ ] Behavioural & social sciences
- [ ] Ecological, evolutionary & environmental sciences

For a reference copy of the document with all sections, see nature.com/documents/nr-reporting-summary-flat.pdf

Life sciences study design

All studies must disclose on these points even when the disclosure is negative.

Sample size	Sample size was estimated with G power software in order to obtain relevant statistical analysis. Based on our previously published and unpublished data, the suggested sample size for in vitro assays is n=6 (size effect 4.87, power: 0.99).
Data exclusions	2 anti-cKit Ab treated animals in the first of the two in vivo experiments were excluded since they died during anesthesia (Figure 3J).
Replication	The measures taken to verify reproducibility included performing the same assay in independent experiments using different donors.
Randomization	The allocation was random.
Blinding	The allocation was random.

Behavioural & social sciences study design

All studies must disclose on these points even when the disclosure is negative.

Study description	N/A
Research sample	N/A
Sampling strategy	N/A
Data collection	N/A
Timing	N/A

Data exclusions	N/A
Non-participation	N/A
Randomization	N/A

Ecological, evolutionary & environmental sciences study design

All studies must disclose on these points even when the disclosure is negative.

Study description	N/A
Research sample	N/A
Sampling strategy	N/A
Data collection	N/A
Timing and spatial scale	N/A
Data exclusions	N/A
Reproducibility	N/A
Randomization	N/A
Blinding	N/A

Did the study involve field work? ☐ Yes ☒ No

Field work, collection and transport

Field conditions	N/A
Location	N/A
Access & import/export	N/A
Disturbance	N/A

Reporting for specific materials, systems and methods

We require information from authors about some types of materials, experimental systems and methods used in many studies. Here, indicate whether each material, system or method listed is relevant to your study. If you are not sure if a list item applies to your research, read the appropriate section before selecting a response.

Materials & experimental systems

n/a	Involved in the study	
☐	☒	Antibodies
☐	☒	Eukaryotic cell lines
☒	☐	Palaeontology and archaeology
☐	☒	Animals and other organisms
☒	☐	Clinical data
☒	☐	Dual use research of concern
☒	☐	Plants

Methods

n/a	Involved in the study	
☒	☐	ChIP-seq
☐	☒	Flow cytometry
☒	☐	MRI-based neuroimaging

Antibodies

Antibodies used	Lineage markers, all FITC-conjugated, include: anti-human CD3 (UCHT1, 1:200, Biolegend, Cat 300406, Lot B390953), anti-human CD4 (RPA-T4, 1:200, Biolegend, Cat 300538, Lot B374653), anti-human CD8 (SK1, 1:200, Biolegend, Cat 344704, Lot B367025), anti-human CD14 (HCD14, 1:400, Biolegend Cat 325604, Lot B416698), anti-human CD15 (HI98, 1:50, Biolegend Cat 394706, Lot B426569), anti-

human CD16 (3G8,1:400, Biolegend Cat 302006, Lot B368715), anti-human CD19 (HIB19, 1:100, Biolegend Cat 392508, Lot B373946), anti-human CD20 (2H7, 1:400, Biolegend Cat 302304, Lot B373724), anti-human CD33 (HIM3-4, 1:400, Biolegend Cat 303304, Lot B396884), anti-human CD34 (581, 1:100, Biolegend Cat 343504, Lot B407007), anti-human CD203c (NP4D6, 1:25, Biolegend Cat 324614, Lot B372625) and anti-human FcεRIα (AER-37, 1:200, Biolegend Cat 334608, Lot B302018). Additional markers used for ILC subsets' identification and characterization include: Brilliant Ultraviolet 395 (BUV395) anti-human CD45 (HI30, 1:100, BD Biosciences Cat 563792, Lot 3268377), Brilliant Violet 421 anti-human CD127 (IL-7Rα) (A019D5, 1:100, Biolegend Cat 351310, Lot B396895), Brilliant Violet 605 anti-human CD117 (c-Kit) (104D2, 1:200, Biolegend Cat 313218, Lot B362518), Alexa Fluor 647 anti-human CRTH2 (CD294) (BM16, 1:200, Biolegend Cat 350104, Lot B373153) and PE anti-human TrkA (REA430, 1: 100, Miltenyi Cat 130-117-705, Lot 5240509055). For some experiments, lineage markers were used as follows: PerCP-Cyanine5.5 anti-human CD3 (UCHT1, 1:200, Biolegend Cat 300430, Lot B361672), PerCP-Cyanine5.5 anti-human CD8 (SK1, 1:100, Biolegend Cat 344710, Lot B330644), PerCP-Cyanine5.5 anti-human CD14 (HCD14, 1:100, Biolegend Cat 325622, Lot B197134), PerCP-Cyanine5.5 anti-human CD16 (3G8, 1:800, Biolegend Cat 302028, Lot B337563) and PerCP-Cyanine5.5 anti-human CD19 (H1B19, 1:50, Biolegend Cat 302230, Lot B361746), PE-Cyanine7 TCRα/β (IP26, 1:100, Biolegend Cat 306719, Lot B298943) and PE-Cyanin7 TCRγ/δ (B1, 1:100, Biolegend Cat 331221, Lot B318208). Dead cells were excluded using the viability Zombie Green Dye (1:2000, Biolegend Cat 423112, Lot B314570).

Human naïve CD4+ T cells were isolated from fresh PBMCs using a FACS Aria II (BD Biosciences) as CD3+CD4+CD45RA+. The following antibodies were used: BUV395 anti-human CD3 (SK7, 1:200, BD Cat 563546, Lot 1214287), PerCP-Cyanine5.5 anti-human CD4 (OKT4, 1:100, Biolegend Cat 317428, Lot B326199) and FITC anti-human CD45RA (HI100, 1:200, Biolegend Cat 304105).

Additional markers used for Treg immune phenotyping include PE anti-human TrkA (REA430, 1:100, Miltenyi Cat 130-117-705, Lot 5240509055), PerCP-Cyanine 5.5 anti-human ICOS (C398.4A, 1:50, Biolegend Cat 313518, Lot B272324), FITC anti-human CD45RA (HI100, 1:200, Biolegend Cat 317428), PE Dazzle 594 anti-human CD127 (A019D5, 1:100, Biolegend Cat 351336, Lot B241297), Brilliant Violet 510 anti-human CD25 (BC96, 1:100, Biolegend Cat 302640, Lot B366218), PE-Cy7 anti-human ICOSL (2D3, 1:50, Biolegend Cat 309410, Lot B295275), PreCP-Cy5.5 anti-human OX40 (Ber-ACT35, 1:50, Biolegend Cat 350010, Lot B279300), Brilliant Violet 786 anti-human OX40L (11C3.1, 1:200, BD Biosciences Cat 743236, Lot 5268391) and Brilliant Violet 711 anti-human Ki67 (Ki-67, 1:200, Biolegend Cat 350516, Lot B468646). Intracellular staining was performed after fixation and permeabilization of the cells with the Foxp3 Transcription Factor Staining Buffer Set (00-5523-00, Invitrogen) using Alexa 700 anti-human FoxP3 (259D/C7, 1:50, BD Biosciences Cat 566935, Lot 2165173).

Phosphorylation staining was performed after fixation with Cytofix Fixation Buffer (BD Biosciences, 554655) and permeabilization with BD Phosflow™ Perm/Wash Buffer I (BD Biosciences, 557885) using PE anti-human RPS6 (Ser235/236, 1:25, Biolegend Cat 608603, Lot B398629).

Murine ILCs were sorted using a FACS Aria II (BD Biosciences) and were identified as lineage negative, CD90.2 positive cells. Lineage markers, all FITC-conjugated include: anti-mouse CD3e (REA641, 1:800, Miltenyi Cat 130-119-798, Lot 5191212768), anti-mouse CD5 (53-7.3, 1:100, Miltenyi Cat 130-102-574, Lot 52107000024), anti-mouse CD8a (53-6.7, 1:200, Miltenyi Cat 130-118-468, Lot 52107000103), anti-mouse CD11b (M1/70.15.11.5, 1:100, Miltenyi Cat 130-113-796, Lot 5191212765), anti-mouse CD11c (REA754, 1:1600, Miltenyi Cat 130-110-700, Lot 5191212780), anti-mouse CD19 (6D5, 1:800, Miltenyi Cat 130-119-800, Lot 5191212715), anti-mouse B220 (RA3-6B2, 1:400, Miltenyi Cat 130-118-462, Lot 5191212709), anti-mouse TCRδ/γ (GL3, 1:50, Miltenyi Cat 130-104-015, Lot 5160322159), anti-mouse TCRβ (REA318, 1:400, Miltenyi Cat 130-104-812, Lot 5191212689), anti-mouse TER119 (Ter-119, 1:800, Miltenyi Cat 130-117-538, Lot 5191212704) and FcεRIα (36951, 1:50, Miltenyi 130-102-264, Lot 5191212685).

Additional markers used for ILC subsets' identification include Brilliant Ultraviolet 737 anti-mouse CD45 (30-F11, 1:1600, BD Biosciences Cat 748371, Lot 169519), Brilliant Violet 605 anti-mouse CD90.2 (53-2.1, 1:1000, Biolegend Cat 140318, Lot B264059), PE-Cyanine7 anti-mouse KLRG1 (2F1/KLRG1, 1:200, Biolegend Cat 138416, Lot B323077), PE anti-mouse ST2 (RMST2-2, 1:100, Invitrogen Cat 12-9335-82, Lot 4324692), Brilliant Violet 711 anti-mouse NK1.1 (PK136, 1:200, Biolegend, Cat 108745, Lot B267734) and Brilliant Violet 650 NKp46 (29A1.A, 1:50, Biolegend Cat 137635, Lot B374401).

Additional markers used include Alexa Fluor 647 anti-mouse FcεRIα (MAR-1, 1:200, Biolegend Cat 134310, Lot B434819), Brilliant Violet 421 anti-mouse CD117 (2B8, 1:400, Biolegend Cat 105828, Lot B361614), Alexa 700 anti-mouse CD11b (M1/70, 1:200 Biolegend Cat 101222, Lot B428765), Brilliant Ultraviolet (BUV737) anti-mouse CD45 (30-F11, 1:1600, BD Biosciences Cat 748371, Lot 169519), FITC anti-mouse CD3 (REA641, 1:800, Miltenyi Cat 130-119-798, Lot 5191212768), AlexaFluor700 anti-mouse CD3 (500A2, 1:400, BD Pharmingen, Cat 557964, Lot 2060194), FITC anti-mouse Ly6C (HK1.4, 1.1600, Biolegend Cat 128022, Lot B290041), PE Dazzle anti-mouse Ly6G (1A8, 1:200, Biolegend Cat 127647, Lot B337525), Brilliant Violet 421 anti-mouse Siglec-F (E502440, 1:200, BD Biosciences Cat 565934, Lot 1138052), Brilliant Violet 711 anti-mouse CD11c (N418, 1:200, Biolegend Cat 117349, Lot B265348), PE-Cyanine7 anti-mouse CD11c (N418, 1:800, Biolegend Cat 117317, Lot B392337), PE-Cyanine7 anti-mouse CD25 (PC61.5, 1:200, Biolegend Cat 101915, Lot B383863), PerCP anti-mouse CD25 (PC61, 1:200, Biolegend Cat 102027, Lot B378944), APC-eFluor 780 anti-mouse F4/80 (BMB, 1:100, Biolegend Cat 157315 Lot B389904), Brilliant Violet 605 anti-mouse CD4 (RM4-5, 1:200, Biolegend Cat 116027, Lot B415523), Spark Blue550 anti-mouse CD4 (L3T4, 1:200 Biolegend Cat 100473 Lot B417887), Brilliant Violet 650 anti-mouse CD8 (53-6.7, 1:100 Biolegend Cat 100788, Lot B354035), Spark Yellow Green 570 anti-mouse CD8 (53-6.7, 1:200 Biolegend, Cat 100788, Lot B354035), Brilliant Violet 750 anti-mouse CD44 (IM7, 1:400, Biolegend Cat 103079 , Lot B394087), Brilliant Violet 711 anti-mouse NK1.1 (PK136, 1:200, Biolegend Cat 108745 , Lot B267734), Brilliant Violet 650 anti-mouse TCR(GL3, 1:100, Biolegend Cat 118147, Lot B407692), Spark UV387 anti-mouse CD90.2 (S2008D, 1:11600, Biolegend Cat 164803, Lot B420409), PE-Fire 810 anti-mouse CD62L (W18021D, 1:400, Biolegend Cat 161205, Lot B413505), PE-Cyanine7 anti-mouse Ter119 (TER-119, 1:400, Biolegend Cat 116221, Lot B374823), PE-Cyanine7 anti-mouse CD19 (1D3/CD19, 1:800, Biolegend Cat 152417, Lot B389427), PE-Cyanine7 anti-human CD5 (53-7.3, 1:1600. Biolegend Cat 100621, Lot B402406) and Zombie UV Fixable Viability kit (1:4000, Biolegend Cat 423108, Lot B407371).

Intracellular staining was performed after fixation and permeabilization of the cells with the FoxP3 Transcription Factor Staining Buffer Set (00-5523-00, Invitrogen) using Alexa Fluor 647 anti-mouse FoxP3 (MF-14, 1:100, Biolegend Cat 126407, Lot B385770), PE-Cy5 anti-mouse FoxP3 (FJK-16S, 1:100, Life Technologies Cat 15-5773-82, Lot 2408301), PerCP-Cy5.5 anti-mouse GATA3 (TWAJ, 1:100, Invitrogen Cat 46-9966-41, Lot 2114184), PerCPeFluor710 anti-mouse GATA3 (TWAJ, 1:100, Life Technologies Cat, Lot), PE-efluor 610 anti-mouse RORγt (B2D, 1:200, Invitrogen Cat 61698182, Lot 2018435), APCeFluor780 anti-mouse IFN(XMG1.2, 1:300, Invitrogen Cat 47-7311-82, Lot 2071355) and PE-eFluor610 anti-mouse IL-13 (eBio13A, 1:100, Life Technologies Cat 4311635, Lot 61-7133-82).

Validation

Each antibody was validated by titrating it on appropriate cells.

Eukaryotic cell lines

Policy information about cell lines and Sex and Gender in Research

Cell line source(s)	Cell lines were owned by the host laboratory or were purchased for this study at Sigma or ATCC: HCV29 (Cat CVCL_8228), RT-4 (Cat HTB-2™), J82 (Cat HTB-1™), TCC-Sup (Cat HTB-5™), MB49 (Cat SCC148) and HMC1 (Cat SCC067).
Authentication	None of the cell lines used were authenticated.
Mycoplasma contamination	All the cell lines were regularly tested for Mycoplasma
Commonly misidentified lines (See ICLAC register)	N/A

Palaeontology and Archaeology

Specimen provenance	*Provide provenance information for specimens and describe permits that were obtained for the work (including the name of the issuing authority, the date of issue, and any identifying information). Permits should encompass collection and, where applicable, export.*
Specimen deposition	*Indicate where the specimens have been deposited to permit free access by other researchers.*
Dating methods	*If new dates are provided, describe how they were obtained (e.g. collection, storage, sample pretreatment and measurement), where they were obtained (i.e. lab name), the calibration program and the protocol for quality assurance OR state that no new dates are provided.*

☐ Tick this box to confirm that the raw and calibrated dates are available in the paper or in Supplementary Information.

Ethics oversight	*Identify the organization(s) that approved or provided guidance on the study protocol, OR state that no ethical approval or guidance was required and explain why not.*

Note that full information on the approval of the study protocol must also be provided in the manuscript.

Animals and other research organisms

Policy information about studies involving animals; ARRIVE guidelines recommended for reporting animal research, and Sex and Gender in Research

Laboratory animals	The studies involved Mus musculus laboratory females and males between 6 and 12 weeks of age breed in house.<br>Wild-type mice C57BL/6 were purchased from the Jackson laboratory. Rorafl/flIl7rCre/+ mice were kindly provided by Prof. A. McKenzie.<br>For tumor experiments only female mice were used, since from a technical point of view the intravesical instillation is compatible with female animal urogenital anatomy.<br>Animals were maintained under a 12h dark/light cycle, at 21°C ± 1 °C and 55% ± 10% of relative humidity. Tumor progression was closely monitored according to an established and approved scoring system. Because tumor burden could not be directly measured due to tumor location, body weight, clinical signs, hydration status and behavioral parameters were assessed daily to ensure animal welfare and to determine humane endpoints. The monitoring was done as described and approved by the Veterinary Authority of the Swiss Geneva Canton (authorizations GE4 and GE300) and predefined burden limits were not exceeded.
Wild animals	The study did not involve wild animals.
Reporting on sex	For tumor experiments only female mice were used, since from a technical point of view the intravesical instillation is straightforward in female animals.
Field-collected samples	This study did not involve field-collected samples.
Ethics oversight	This study was approved by the Veterinary Authority of the Swiss Geneva Canton (authorization GE4, GE300).

Note that full information on the approval of the study protocol must also be provided in the manuscript.

Clinical data

Policy information about clinical studies

All manuscripts should comply with the ICMJE guidelines for publication of clinical research and a completed CONSORT checklist must be included with all submissions.

Clinical trial registration	N/A

Study protocol	N/A
Data collection	N/A
Outcomes	N/A

Dual use research of concern

Policy information about [dual use research of concern](#)

Hazards

Could the accidental, deliberate or reckless misuse of agents or technologies generated in the work, or the application of information presented in the manuscript, pose a threat to:

No	Yes	
☒	☐	Public health
☒	☐	National security
☒	☐	Crops and/or livestock
☒	☐	Ecosystems
☒	☐	Any other significant area

Experiments of concern

Does the work involve any of these experiments of concern:

No	Yes	
☒	☐	Demonstrate how to render a vaccine ineffective
☒	☐	Confer resistance to therapeutically useful antibiotics or antiviral agents
☒	☐	Enhance the virulence of a pathogen or render a nonpathogen virulent
☒	☐	Increase transmissibility of a pathogen
☒	☐	Alter the host range of a pathogen
☒	☐	Enable evasion of diagnostic/detection modalities
☒	☐	Enable the weaponization of a biological agent or toxin
☒	☐	Any other potentially harmful combination of experiments and agents

Plants

Seed stocks	*Report on the source of all seed stocks or other plant material used. If applicable, state the seed stock centre and catalogue number. If plant specimens were collected from the field, describe the collection location, date and sampling procedures.*
Novel plant genotypes	*Describe the methods by which all novel plant genotypes were produced. This includes those generated by transgenic approaches, gene editing, chemical/radiation-based mutagenesis and hybridization. For transgenic lines, describe the transformation method, the number of independent lines analyzed and the generation upon which experiments were performed. For gene-edited lines, describe the editor used, the endogenous sequence targeted for editing, the targeting guide RNA sequence (if applicable) and how the editor was applied.*
Authentication	*Describe any authentication procedures for each seed stock used or novel genotype generated. Describe any experiments used to assess the effect of a mutation and, where applicable, how potential secondary effects (e.g. second site T-DNA insertions, mosiacism, off-target gene editing) were examined.*

ChIP-seq

Data deposition

☐ Confirm that both raw and final processed data have been deposited in a public database such as [GEO](#).

☐ Confirm that you have deposited or provided access to graph files (e.g. BED files) for the called peaks.

Data access links<br>*May remain private before publication.*	*For "Initial submission" or "Revised version" documents, provide reviewer access links. For your "Final submission" document, provide a link to the deposited data.*
Files in database submission	*Provide a list of all files available in the database submission.*

Genome browser session (e.g. UCSC) | *Provide a link to an anonymized genome browser session for "Initial submission" and "Revised version" documents only, to enable peer review. Write "no longer applicable" for "Final submission" documents.*

Methodology

Replicates	*Describe the experimental replicates, specifying number, type and replicate agreement.*
Sequencing depth	*Describe the sequencing depth for each experiment, providing the total number of reads, uniquely mapped reads, length of reads and whether they were paired- or single-end.*
Antibodies	*Describe the antibodies used for the ChIP-seq experiments; as applicable, provide supplier name, catalog number, clone name, and lot number.*
Peak calling parameters	*Specify the command line program and parameters used for read mapping and peak calling, including the ChIP, control and index files used.*
Data quality	*Describe the methods used to ensure data quality in full detail, including how many peaks are at FDR 5% and above 5-fold enrichment.*
Software	*Describe the software used to collect and analyze the ChIP-seq data. For custom code that has been deposited into a community repository, provide accession details.*

Flow Cytometry

Plots

Confirm that:

- [x] The axis labels state the marker and fluorochrome used (e.g. CD4-FITC).
- [x] The axis scales are clearly visible. Include numbers along axes only for bottom left plot of group (a 'group' is an analysis of identical markers).
- [x] All plots are contour plots with outliers or pseudocolor plots.
- [ ] A numerical value for number of cells or percentage (with statistics) is provided.

Methodology

Sample preparation	Isolated PBMCs were immediately stained for 20 minutes at room temperature in sorting buffer (PBS, 50 µM EDTA, 0.2% BSA) with the proper antibody mix and dead cell staining reageant. Intracellular staining was performed after fixation and permeabilization of the cells with the FoxP3 Transcription Factor Staining Buffer Set.
Instrument	Samples were acquired on a Fortessa flow cytometer (BD) or a Cytek Aurora spectral flow cytometer (Cytek Biosciences).
Software	BD FACSDiva and Cytek Spectroflo were used to collect the data. FlowJo software_v10.7.1 (TreeStar) was used to analyse the data.
Cell population abundance	In the post-sort fraction the abundance of the relevant cell population was equal or higher than 95%. the purity was determined by requiring an aliquot of the sorted sample.
Gating strategy	Human ILCs were identified as lineage (Lin) negative and CD127 positive cells. ILC subsets were identified as CRTH2+ (ILC2), CRTH2-cKIT- (ILC1), CRTH2-cKIT+ (ILCP). Human naïve CD4+ T cells were isolated from fresh PBMCs as CD3+CD4+CD45RA+. Murine mast cells were identified as CD45+ FcεRIα+CD117+. Murine ILCs were identified as lineage negative, CD90.2 positive cells.

- [x] Tick this box to confirm that a figure exemplifying the gating strategy is provided in the Supplementary Information.

Magnetic resonance imaging

Experimental design

Design type	*Indicate task or resting state; event-related or block design.*
Design specifications	*Specify the number of blocks, trials or experimental units per session and/or subject, and specify the length of each trial or block (if trials are blocked) and interval between trials.*
Behavioral performance measures	*State number and/or type of variables recorded (e.g. correct button press, response time) and what statistics were used to establish that the subjects were performing the task as expected (e.g. mean, range, and/or standard deviation across subjects).*

Acquisition

Imaging type(s) — *Specify: functional, structural, diffusion, perfusion.*

Field strength — *Specify in Tesla*

Sequence & imaging parameters — *Specify the pulse sequence type (gradient echo, spin echo, etc.), imaging type (EPI, spiral, etc.), field of view, matrix size, slice thickness, orientation and TE/TR/flip angle.*

Area of acquisition — *State whether a whole brain scan was used OR define the area of acquisition, describing how the region was determined.*

Diffusion MRI ☐ Used ☐ Not used

Preprocessing

Preprocessing software — *Provide detail on software version and revision number and on specific parameters (model/functions, brain extraction, segmentation, smoothing kernel size, etc.).*

Normalization — *If data were normalized/standardized, describe the approach(es): specify linear or non-linear and define image types used for transformation OR indicate that data were not normalized and explain rationale for lack of normalization.*

Normalization template — *Describe the template used for normalization/transformation, specifying subject space or group standardized space (e.g. original Talairach, MNI305, ICBM152) OR indicate that the data were not normalized.*

Noise and artifact removal — *Describe your procedure(s) for artifact and structured noise removal, specifying motion parameters, tissue signals and physiological signals (heart rate, respiration).*

Volume censoring — *Define your software and/or method and criteria for volume censoring, and state the extent of such censoring.*

Statistical modeling & inference

Model type and settings — *Specify type (mass univariate, multivariate, RSA, predictive, etc.) and describe essential details of the model at the first and second levels (e.g. fixed, random or mixed effects; drift or auto-correlation).*

Effect(s) tested — *Define precise effect in terms of the task or stimulus conditions instead of psychological concepts and indicate whether ANOVA or factorial designs were used.*

Specify type of analysis: ☐ Whole brain ☐ ROI-based ☐ Both

Statistic type for inference — *Specify voxel-wise or cluster-wise and report all relevant parameters for cluster-wise methods.*

(See Eklund et al. 2016)

Correction — *Describe the type of correction and how it is obtained for multiple comparisons (e.g. FWE, FDR, permutation or Monte Carlo).*

Models & analysis

n/a	Involved in the study
☐	☐ Functional and/or effective connectivity
☐	☐ Graph analysis
☐	☐ Multivariate modeling or predictive analysis

Functional and/or effective connectivity — *Report the measures of dependence used and the model details (e.g. Pearson correlation, partial correlation, mutual information).*

Graph analysis — *Report the dependent variable and connectivity measure, specifying weighted graph or binarized graph, subject- or group-level, and the global and/or node summaries used (e.g. clustering coefficient, efficiency, etc.).*

Multivariate modeling and predictive analysis — *Specify independent variables, features extraction and dimension reduction, model, training and evaluation metrics.*
