# Supplementary material for: Mast-cell derived nerve growth factor drives ILC2 pro-tumoral functions in bladder cancer
Source: Nat Commun. 2026 Feb 21;17:3061. doi: 10.1038/s41467-026-69841-y (PMC13039139; doi:10.1038/s41467-026-69841-y)
Supplement: Supplementary file 3 — Transparent Peer Review file [file 41467_2026_69841_MOESM3_ESM.pdf]

nature portfolio

Peer Review File

Mast-cell derived nerve growth factor drives ILC2 pro-tumoral functions in bladder cancer

Corresponding Author: Professor Camilla Jandus

This file contains all reviewer reports in order by version, followed by all author rebuttals in order by version.

Version 0:

Reviewer comments:

Reviewer #1

(Remarks to the Author)
In their manuscript, Falquet et al. explore how NGF-induced TrkA signaling in iLC2 cells facilitates Treg induction in bladder cancer, positing that counteracting TrkA signaling in vivo could prime tumors for more effective ICB treatment. While the involvement of NGF-TrkA in tumorigenesis has been previously documented in various cancer types, its specific impact on iLC2 cells and its novel application to bladder cancer offer a fresh perspective. The study, supported by data from both human patients and animal models, suggests a promising avenue for enhancing anti-tumor immunity. However, the robustness of their experimental evidence and scientific analyses could be enhanced.

The main concerns are as follows:

1. The observation that iLC2 deficiency leads to a significant reduction in intratumoral Tregs and MDSCs, without a corresponding increase in the frequency of CD4+, CD8+, NKT, or NK cells (as shown in Figure S5), raises questions about the mechanism of enhanced anti-tumor immunity. A more thorough analysis of the effector T/NK cell function, absolute numbers, and their frequencies is required to clarify this paradox.

2. The hypothesis that mast cells are the primary source of NGF production within bladder tumors lacks conclusive support from the data presented. Figure 4B indicates that mast cells comprise less than 3% of the tumor-infiltrating leukocytes, suggesting a minor contribution to the overall tumor mass. Figure 4C reveals a statistically insignificant twofold reduction in NGF mRNA production by cancer cells compared to mast cells, a difference that becomes even less compelling when considering the substantial mass of tumor cells. Figure 4F demonstrates that the majority of NGF-producing cells are not mast cells, necessitating a more rigorous investigation into the cellular sources of NGF within the tumor.

3. Several figures, including Figures 3C, 4C, 4D, 4F, and 5F, rely on sample sizes too small to draw firm conclusions, undermining the strength of the findings.

Minor issues that need addressing include:

1. The strong correlation between mast cell genes and NGF mRNA levels in the TCGA database, while intriguing, does not definitively indicate production. It's worth considering whether mast cells might also respond to NGF signals. Do intratumoral mast cells express TrkA on their surface?

2. The authors suggest that mast cells recruit iLC2 to the tumor bed. If this is the case, iLC2 cells should be located near mast cells. Is there any evidence supporting this spatial relationship?

Reviewer #2

(Remarks to the Author)
This paper describes the negative role of ILC2 in bladder cancer and the underlying mechanism driving ILC2-mediated tumor progression using animal models and human samples. This work builds directly on previous findings from the same group, showing that ILC2s in bladder cancer drive the recruitment of MDSCs into the tumor, thereby promoting an

immunosuppressive microenvironment. Here the novelty relies on the mechanism triggering ILC2 effector function in bladder cancer which involves the secretion of NGF by mast cells that activates TrKa-expressing ILC2s, resulting in tumor growth and progression. Most experiments are well performed and appropriate. However, some key experiments are missing, preventing conclusions about the specificity and importance of the described mechanisms.

Comments:

Figure 1. Survival analyses. Can the authors describe the rationale for categorizing NGF expression into four quartiles? Can the authors perform survival analyses based on median expression. Is there evidence/data to suggest that normal bladder tissue express low RNA levels of NGF that would support the use of a cutoff value that could be applied to the bladder cancer patient cohort. This threshold would correspond to the level of NGF expression found in healthy bladder tissue, perhaps supporting the categorization into four quartiles and explaining why only the first quartile performs better in terms of survival. In addition, does the level of NGF correlate with disease stage, tumor grade, or other clinical covariates?

Figure S1. Based on qPCR analyses, the authors found that “human bladder cancer cell lines showed barely detectable transcripts of neurotrophic receptors” and, based on these data, concluded that “NGF might preferentially signal through TrKa+ ILC2s, rather than directly on tumor cells”. However, the authors did not use the same housekeeping gene for normalisation and therefore, it is complicated to compare the levels of expression found in ILC2s to tumor cell lines. To validate their observations, multiplex IHC staining should be performed using CD45, CD3, GATA3 and TRKA on tumor samples to estimate Trka expression on ILC2s and other cell types in the tumor microenvironment. This multiplex IHC can be extended to NGF expression and TPSAB1 (figure 4) to show the colocations or interaction between Mast cell derived NGF expression and Trka expressing ILC2s.

Figure 2 and Figure S2. As mentioned by the authors in the discussion, TrKa expression on ILC2s is heterogenous. Did the authors observe a difference in cytokine production between TrKa positive and negative ILC2s? Does use of GW441756 impair NGF-induced ILC2 transmigration and chemotaxis and reduce pS6, pERK and pCREB levels. Did the authors perform a dose response for NGF and TrKa inhibitor GW441756 (IC50 2nM and the authors used it at a concentration of 10uM). Did the authors confirm their findings using mouse ILC2s that would support the use of mouse models in subsequent experiments to study the role of NGF and ILC2s in bladder cancer progression. On a side note, error bars for multiple panels are missing.

Figure 3. Is there a positive correlation between the level of NGF found in bladder and tumor burden (tumor weight)? This would further suggest a role for NGF in tumor progression.

Figures 4 and 5. A direct link between mast cell-derived NGF secretion and ILC2 function is missing. Given that TPSAB1 negative cells also express NGF (Figure 4F), a mast cell independent pathway might be involved. What is the identity of these cells? To demonstrate the specificity of this pathway, the authors should use a combination of in vitro culture systems (e.g co-culture of mast cells and ILC2s or the culture of ILC2s in mast cell conditioned media in the presence or not of TrKa inhibtor) and conditional mouse models (Ngf floxed animals [RMRC 13175] crossed to mast cell specific cre transgenic mice]. Furthermore, does the depletion of Tregs (e.g. Foxp3DTR model) or the ablation of mast cells (e.g. KO mouse models) phenocopy the outcomes observed in ILC2 deficient mice. In addition, the RORafl/flIL7Rcre mice display reduced ILC1s and dysfunctional ILC3s compared to littermate controls. Can the authors perform rescue experiments with WT or CRISPRKO Trka ILC2 in ILC2 deficient mice to ascertain of the specificity of the pathway as both ILC1s and ILC3s express low levels of Ntrk1 (Figure 3F).

Figure S5. Can the authors indicate the absolute cell numbers for all the indicated populations.

Figure 6. It would be important to show that TrKa inhibition phenocopies the observations made in ILC2 deficient mice. Specifically, the authors should assess the frequency and number of Tregs in the tumor after treatment. Are improved survival outcomes associated with decreased ILC2 cytokine production? The data presented in Figure S6A are critical but, given the small sample size, no firm conclusions can be drawn. In addition, does intravesical delivery of NGF promote tumor growth, associated with increased production of ILC2-derived cytokines and accumulation of intratumoral Tregs?

Discussion. Regarding the data presented in Figure S6C, the authors conclude that “strong survival reduction [are observed] in gastric and colorectal cancer patients with high NGF transcript levels and ILC2 infiltration”. As there is no direct correlation between NGF levels, ILC2 infiltration and survival in these tumors, the authors either soften this statement or provide the data supporting this claim.

Material and methods. The authors should indicate whether male or female mice were used in experiments as sex-differences have been reported on ILC2s. Also, in MMT assay, it is GW441756 and not GW5441756.

Figure legends. The authors should indicate the exact sample size for each panel in the figure legends as well as the number of times that each reported experiment were repeated. All experiments should be repeated 2 or 3 times for data reliability.

Lastly, a more balanced view or increased discussion of the pro and anti-tumorigenic functions of ILC2s would have been appropriate, as well as reference to original research articles rather than reviews.

Reviewer #3

(Remarks to the Author)
The manuscript by Falquet et al investigates ILC2 functions in bladder cancer, using patient-derived specimen, in vitro experiments and mouse models, as well as inferring publicly available RNAseq data including large cohorts of patients. The main claim of the paper is that a mast cell-NGF-ILC2-Treg axis supports bladder cancer growth via mast cell derived NGF, which activates ILC2 to induce Treg, favoring tumor escape.
The story is interesting and holds translational value given by in vivo experiments with a TrkA inhibitor, also in association with anti PD-1. Yet, most of the results are correlative than mechanistic and conclusions are not fully supported by the data provided. Several issues should be addressed:

1) It is not clear why the authors chose to study NGF, among all the many neuronal derived molecules that can potentially activate ILC2s. The rationale should be expanded in the introduction or at the beginning of the result section.

2) Fig 1. How many human samples were used for NGF quantification in human bladder tissues/urines from cancer patients and healthy donors? This should be specified in the figure legend.

3) Throughout the manuscript, please clearly indicate how many mice were used for in vivo/ex vivo experiments

4) Immunohistochemistry/immunofluorescence on tumor tissues (human and mouse) should be provided to show TrkA expression. Indeed, authors only consider ILC2s as possible sensors of NGF in the tumor microenvironment. Even if tumor cells are shown to have negligible levels of TrkA, several other stomal cells could express it.

5) Double immunofluorescence for Triptase and NFG is instead provided (Fig 4F). Yet, the quality of the staining is poor, with high background. The staining also shows NGF+ Tryptase- cells, therefore arguing for other sources of NGF rather than mast cells within the tumor. This issue should be expanded in the discussion.

6) Fig 1F and S1B. Differently from what stated in the text, NTRK1 seems to be expressed by all the ILCs subsets and just overexpressed in ILC2s.

7) Fig. 1G. How many patients were used for this analysis?

8) Fig. 1H. TrkA protein is not significantly higher in ILC2s from patients related to healthy donors. Does that mean that the NGF-TrkA axis is in place also in absence of the tumor? This seems at odds with survival data shown in Fig. 1. This is even more important considering that NGF stimulation induces cytokine production in ILC2s from bladder cancer patients and healthy donors as well (Fig. S2B).
Mast cell accumulation shown in tumors could count for the mechanism being enhanced in patients. This aspect should be addressed in the discussion.

9) Fig S1I and S3E. Real time PCR for TrkA on tumor cells should include a ILC2 sample to facilitate comparison.

10) Fig2 D-F. Histograms show very tiny differences between control and NFG-stimulated conditions. To best evaluate the differences, data should be quantified using mean fluorescence intensity rather than percentage of positive cells. An FMO control (i.e. a sample not stained with the desired antibody) should also be included.

11) It is not clear why mitochondrial fitness was measured as redout of NGF stimulation in ILC2s (Fig. S2E).

12) Fig 3C. The comparison should be between mast cells and tumor cells both sorted ex vivo from the bladder of the MB49 instilled mice. Indeed, microenvironment stimulation could enhance NGF production in tumor cells that are conversely negative during in vitro culture.

13) Mast cell data seems most correlative than mechanistic. Authors should consider in vitro co-culture experiments between mast cells and ILC2s, also in presence of the TrkA inhibitor, to actually prove that MC can activate ILC2s via NGF production. Readout of such experiments should be cytokine production as shown in figure 2.

14) Data from ILC2KO mice are impressive (Fig.5). Yet, these results do not directly prove that the reduction of Treg is due to the absence of ILC2s. Indeed, ILC2KO mice have also reduced frequencies of MDSC and eosinophils. Authors cannot exclude a bystander effect on Tregs, given the well-known MDSC-Treg crosstalk and the fact that eosinophils can produce TGF , a master Treg-polarizing cytokine. This issue should be at least discussed.

15) The mast cell- Treg crosstalk should be discussed as well, in the frame of the existing literature.

16) A key issue is that ILC2s functions that count for Treg accumulation/activity are underexplored. Authors report an in vitro experiment (Fig. 5I) showing that ILC2s can induce Tregs from naïve CD4 T cells. Nevertheless, this experiment does not address the involvement of the NGF/TrkA axis in this phenomenon. Control conditions in which ILC2 are pretreated with NGF or pre-cultured with mast cells, in presence or absence of the TrkA inhibitor, should be added.

17) Also, this experiment does not explore the mechanisms by which ILC2s promote Treg conversion. Is this mediated by

cytokine production? Treg conversion is usually induced, at least in vitro, by TGF and IL2, which however were not measured in NGF-treated ILC2s (experiments in Fig.2).
Also, authors suggest a contact-dependent mechanism (page 8 line 225), but this should be proven or excluded with transwell experiments, and the rationale of this conclusion is lacking.

18) Figure 6. Which are the frequencies of tumor infiltrating Tregs in mice treated with the TrkA inhibitor? This is a key information to be provided to add mechanistic cues to the author’s hypothesis.

19) As BCG is the treatment choice for bladder cancer patients, did the authors consider treating tumor bearing mice with the combination of TrkA inhibitor and BCG?

Reviewer #4

(Remarks to the Author)
This paper holds significant relevance in the field of ILCs in bladder cancer. Given the limited existing research in this area, the manuscript builds upon the foundation laid by a previous paper on the modulation of the T cell/MDSC ratio by ILC2s in bladder cancer. The study explores the novel NGF-TrkA-ILC2s axis's role in the development and progression of bladder cancer. It highlights the potential of TrkA blockade as an innovative therapeutic approach for bladder cancer patients, especially those resistant to immune checkpoint therapy, and the potential of the combination of anti-PD1 and GW441756. The study effectively combines human and mouse models and will be a relevant addition to the intratumoral ILC field in bladder cancer.
Fig. 1 - Include the number of healthy donors and cancer patients in figure legends, may also be a good idea to include the identifying markers for human ILCs in the legend since there is so much confusion about the gating strategy. (also in the S1D legend need details about the identification strategy for ILC1, 2, ILC3 clearly)
How long were the ILC2s cultured for short-term expansion?
Fig 5C – will be useful to include a regular graph showing the difference in bladder tumor weights instead of just a picture.
While day 1 and day 3 are important to understand the immune landscape of the early tumor microenvironment, immune analysis at a more traditional sacrifice endpoint may yield critical information on the NGF-TrkA-ILC2s axis.
If possible, Single-cell spatial techniques will be extremely helpful in delineating the interactions of ILC2s with other immune cells (including Tregs) and stromal cells and will give a broader picture of the interaction of ILC2s with the immune landscape.
To confirm that ILC2s are working through Tregs, Tregs need to be depleted or Treg KO mice need to be used. Since there are other immune cells involved, this will investigate the extent to which Tergs contribute to ILC2-mediated effects.
Overall, the paper has exciting findings that will take the field forward and is also translationally relevant in the case of BCG nonresponsive tumors.

Version 1:

Reviewer comments:

Reviewer #2

(Remarks to the Author)
I thank the authors for their revised version of the manuscript. The authors responded satisfactorily to all my comments. The quality of the manuscript has been significantly improved, and the conclusions are supported by the experimental data. I only have three minor comments:
• Line 528 – “Additional markers used for Treg immune phenotyping used include…” – the second “used” should be deleted.
• Line 635 – There is a typo – it is microcentrifuge
• Before publication, the authors should ensure that the Bulk RNA seq dataset has been deposited in the NCBI Gene Expression Omnibus

Reviewer #3

(Remarks to the Author)
The manuscript has been substantially improved with the addition of numerous new experiments. Many of these address my initial requests. Indeed, the relevance of mast cells (MC) as the main source of NGF, which in turn stimulates/activates TrkA+ ILC2, in this tumor setting has been proved. However, some of the new results raise further questions, as they appear to conflict with the authors’ original hypotheses.

1. The new in vitro experiment shown in Figure 4G clearly demonstrates that ILC2 induce the conversion of naive T cells into Tregs in vitro, through a mechanism that is independent of NGF. If this is true, it suggests that the MC-NGF-ILC2- axis and the ILC2-Treg axis operate independently from each other. Nevertheless, both are relevant to sustain tumor growth as demonstrated by new experiments in mice depleted of either MC (treated with anti cKIT antibody) or Treg (DEREG mice).

2. In this new scenario, the MC-derived NGF axis activates ILC2 to produce type 2 cytokines with pro-tumoral functions (the authors previously showed that ILC2-derived IL-13 rewires an immunosuppressive TME towards bladder cancer growth, ref.6 of the manuscript), but these cytokines are not required for Treg conversion. Indeed, while the authors show that T cell-ILC2 contact induces IL-5 and IL-13 production (Figure 4J), these same cytokines are also produced by ILC2 following HMC1 stimulation and are reduced if the NGF receptor is blocked (Figure 2F). Yet, stimulation with NGF or blockade of its receptor does not alter the ability of ILC2 to convert Tregs (Figure 4G).

3. Yet it is undoubted from data in figure 4 (experiments in ILC2 KO mice) that the absence of ILC2 impairs the accumulation/differentiation of Treg. The most likely relevant mechanism for Treg induction by ILC2 therefore remains stimulation via OX40 and ICOS. The authors should show that ILC2 express OX40L and ICOSL, and whether these molecules are modulated (probably not) by NGF stimulation or co-culture with MC. An in vitro experiment using blocking antibodies against OX40 and ICOS, as well as IL-5 and IL-13 blockers, should definitively clarify the mechanism.

4. A possible interpretation linking MC-derived NGF to Tregs might instead arise from the data shown in Figure S11 B-C. Mice treated with GW441756 exhibit fewer ILC2 and also fewer Tregs. It is possible that NGF has a chemoattractive effect on ILC2. This data were present in the original manuscript (in vitro experiments in Figure 2C) but were later removed at revision stage for unclear reasons. If this is the case, the new mechanistic hypothesis could be that the MC-NGF axis serves to recruit ILC2 to the tumor, which then mediate Treg conversion via OX40L-OX40 and ICOSL-ICOS interactions.

These points need to be clarified experimentally; otherwise, some of the conclusions in the manuscript and in the abstract should be revised and toned down.
Finally, as a minor point, I suggest to rephrase text and legend od figure S11 B-C to clarify that GW441756 was given in wild type (not ILC2 KO) tumor bearing mice.

Version 2:

Reviewer comments:

Reviewer #3

(Remarks to the Author)
I commend the authors for their careful revisions. I have no further concerns.

Open Access This Peer Review File is licensed under a Creative Commons Attribution 4.0 International License, which permits use, sharing, adaptation, distribution and reproduction in any medium or format, as long as you give appropriate credit to the original author(s) and the source, provide a link to the Creative Commons license, and indicate if changes were made.
In cases where reviewers are anonymous, credit should be given to 'Anonymous Referee' and the source.
The images or other third party material in this Peer Review File are included in the article’s Creative Commons license, unless indicated otherwise in a credit line to the material. If material is not included in the article’s Creative Commons

license and your intended use is not permitted by statutory regulation or exceeds the permitted use, you will need to obtain permission directly from the copyright holder.
To view a copy of this license, visit https://creativecommons.org/licenses/by/4.0/

REVIEWER COMMENTS

Reviewer #1 (Remarks to the Author): with expertise in cancer immunology

In their manuscript, Falquet et al. explore how NGF-induced TrkA signaling in iLC2 cells facilitates Treg induction in bladder cancer, positing that counteracting TrkA signaling in vivo could prime tumors for more effective ICB treatment. While the involvement of NGF-TrkA in tumorigenesis has been previously documented in various cancer types, its specific impact on iLC2 cells and its novel application to bladder cancer offer a fresh perspective. The study, supported by data from both human patients and animal models, suggests a promising avenue for enhancing anti-tumor immunity. However, the robustness of their experimental evidence and scientific analyses could be enhanced.

We would like to thank the reviewer for their interest in our manuscript and all the constructive comments. We have conducted several new experiments and modified the manuscript to address their questions, as detailed below.

The main concerns are as follows:

1. The observation that iLC2 deficiency leads to a significant reduction in intratumoral Tregs and MDSCs, without a corresponding increase in the frequency of CD4+, CD8+, NKT, or NK cells (as shown in Figure S5), raises questions about the mechanism of enhanced anti-tumor immunity. A more thorough analysis of the effector T/NK cell function, absolute numbers, and their frequencies is required to clarify this paradox.
Answer to reviewer: as suggested by the reviewer, we have performed a more comprehensive, longitudinal analyses of the tumor immune infiltrate in littermate and ILC2 KO animals. We have developed and validated a 28-marker panel for ex-vivo monitoring of immune cell subsets by spectral flow cytometry. These analyses have been performed on day 0 (prior tumor instillation) and on day 1 and 3 post-tumor instillation. We confirm the increase of MDSCs, Tregs and eosinophils, alongside with neutrophils, in tumor-bearing littermates as compared to ILC2KO mice (revised Figure 4B, Supplementary Figure 8). For T and NK cells, we did not observe major differences in terms of subset differentiation (see **Figure 1 here below**), but we found a trend for heightened IFNγ^+ CD8 T cells and a significant increase of the CD8/Treg ratio, a parameter that has been shown to highly correlate with strong anti-tumor immunity in many cancer types (revised Supplementary Figure 9, page 8-9).

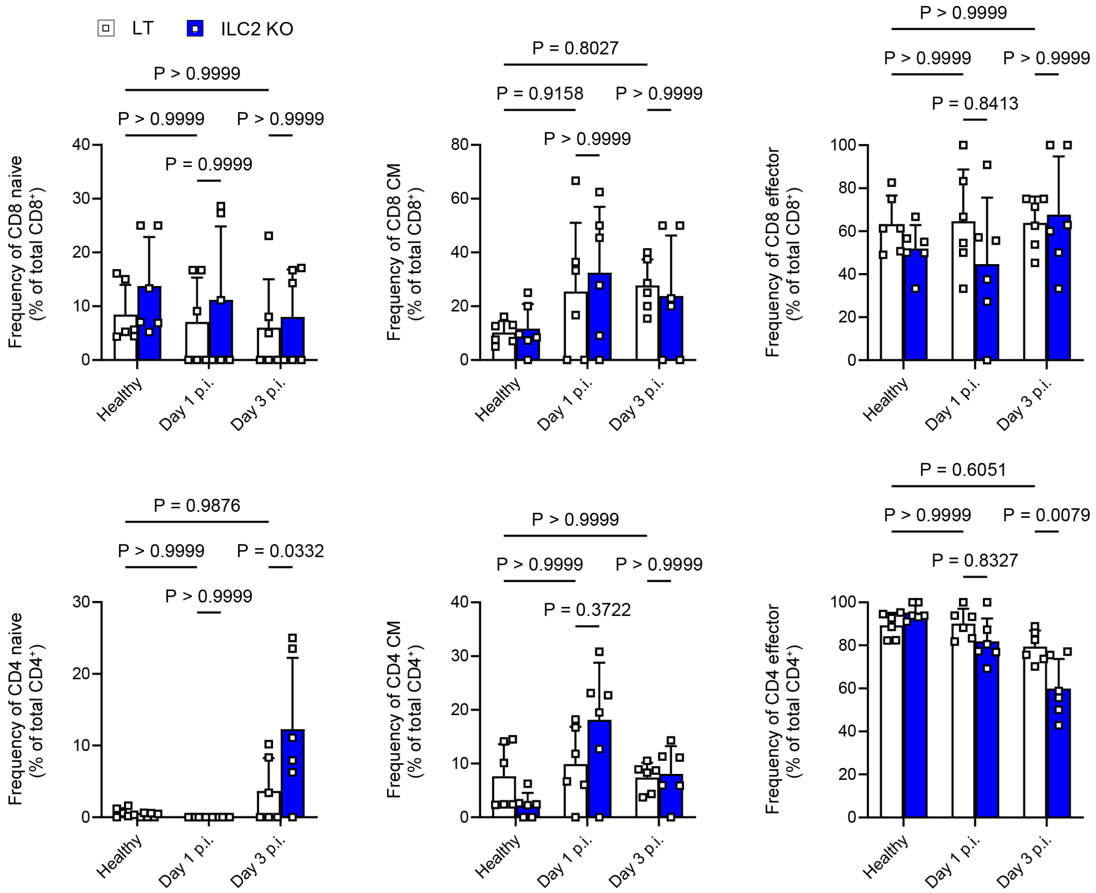

Figure 1. T cell subset distribution in tumor-bearing littermates (LT) and ILC2KO animals. Frequency of naïve, central memory (CM) and effector CD8 (upper graphs) and CD4 (lower graphs) T cells in healthy and tumor bearing $ROR\alpha^{fl/fl}Il7r^{Cre-}$ (LT, white bars) and $ROR\alpha^{fl/fl}Il7r^{Cre+}$ (ILC2 KO, blue bars) mice on day 1 (Day 1 p.i.) and day 3 (Day 3 p.i.) post-MB49 instillation, determined by flow cytometry.

2. The hypothesis that mast cells are the primary source of NGF production within bladder tumors lacks conclusive support from the data presented. Figure 4B indicates that mast cells comprise less than 3% of the tumor-infiltrating leukocytes, suggesting a minor contribution to the overall tumor mass. Figure 4C reveals a statistically insignificant twofold reduction in NGF mRNA production by cancer cells compared to mast cells, a difference that becomes even less compelling when considering the substantial mass of tumor cells. Figure 4F demonstrates that the majority of NGF-producing cells are not mast cells, necessitating a more rigorous investigation into the cellular sources of NGF within the tumor.

Answer to reviewer: we thank the reviewer for the suggestion. To address this, we have performed several experiments, both using human and mouse samples:

1) we have sorted bladder-infiltrating mast cells and bladder cancer cells directly from tumor-bearing bladders and we confirmed an increase in NGF mRNA transcripts in mast cells as compared to tumor cells (p=0.0556), further suggesting their involvement as NGF source in our setting (revised Figure 3H)

As for patients, we agree with the reviewer that the quality of our in situ staining for NGF (shown in original Figure 4F) was not optimal. To strengthen our data:

2) we have performed co-staining for Tryptase (as surrogate marker for mast cells) and NGF. We observed co-localization of NGF with Tryptase^{+} cells (as shown in the revised Figure 2D). Moreover, by comparing the NGF content in mast cells in healthy versus tumor bearing bladder from patients, we observed significant enrichment of NGF in the disease setting (revised Figure 2D, right graph).

3) To strengthen the missing link between mast cells-NGF and ILC2s we have performed both *in vitro* and *in vivo* experiments.

   For *in vitro* experiments, we have used the mast cell line HMC1 and showed that upon activation it produces NGF (revised Supplementary Figure S4B). Hence, we have co-cultured HMC1 cells with ILC2s, pre-treated or not with the TrkA inhibitor GW441756. As a readout, we quantified the cytokines in the supernatant after 48 hours (revised Figure 2F). No Type 2 cytokines were secreted by mast cells only. Instead, a strong increase in Type 2 cytokine secretion was observed when mast cells were co-cultured with ILC2s, an effect that was at least partially abrogated by the TrkA inhibitor. Altogether, these results suggest that NGF is secreted by mast cells in quantities sufficient to induce in vitro ILC2 functional activation. We present this data in the manuscript at page 7.

   To define the *in vivo* relevance of this finding, we performed an antibody-based depletion of mast cells, using the ACK2 anti-cKit antibody, previously reported by others for mast cell *in vivo* depletion experiments. We observed a significant survival advantage in mice treated with the anti-cKit antibody in comparison to the untreated animals, supporting the fact that mast cells have a protumoral function in our model (revised Figures 3I-J, page 8).

3. Several figures, including Figures 3C, 4C, 4D, 4F, and 5F, rely on sample sizes too small to draw firm conclusions, undermining the strength of the findings.
<u>Answer to reviewer</u>: we fully agree with the reviewer that some of our initial results lacked sufficient sample size to have statistical power to draw sound conclusions. We have included new experimental replicates and indicated in the Figure legends the number of replicates and how many times the experiments were performed.

Minor issues that need addressing include:

1. The strong correlation between mast cell genes and NGF mRNA levels in the TCGA database, while intriguing, does not definitively indicate production. It's worth considering whether mast cells might also respond to NGF signals. Do intratumoral mast cells express TrkA on their surface?
<u>Answer to reviewer</u>: the reviewer is raising an interesting point. It is indeed well known that mast cells can express TrkA. To assess if bladder mast cells express TrkA, we have performed co-staining for Tryptase and TrkA on human bladder tissue sections. As shown in **Figure 2 here below** and in the revised Figure 1I, we observed TrkA expression on mast cells, but also on other cell types, particularly ILC2s. Moreover, as outlined in the graph in **Figure 2**, the expression level of TrkA tend to be higher in cells other than mast cells.

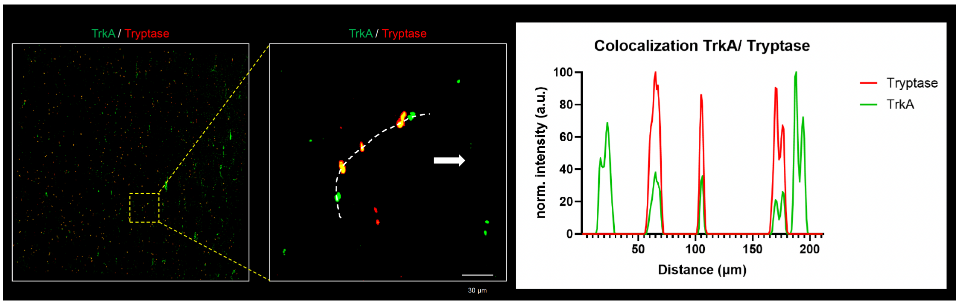

Figure 2. Tissue section was immunostained for mast cells (Tryptase, red) and TrkA (green) and imaged using confocal fluorescence microscopy. A selected region of interest within the merged imaged is indicated by a dashed square and corresponds to the magnified area shown in the adjacent image. Colocalization of TrkA and Tryptase

is demonstrated by overlapping intensity profiles (TrkA: green line; tryptase: red line) measured along the segmented white dotted line shown in the upper panels of individual channels. Scale bar, 30 µm.

2. The authors suggest that mast cells recruit iLC2 to the tumor bed. If this is the case, iLC2 cells should be located near mast cells. Is there any evidence supporting this spatial relationship?

Answer to reviewer: to determine if ILC2s can sense mast-cell derived NGF, bladder tissue sections were co-stained for Tryptase, CD45, GATA3 and CD3. As illustrated in the revised Figure 2E, mast cells are located near ILC2s in the bladder tissues of patients, suggesting that NGF released by mast cells might be sensed by neighboring ILC2s.

Reviewer #2 (Remarks to the Author): with expertise in ILC2, cancer immunology

This paper describes the negative role of ILC2 in bladder cancer and the underlying mechanism driving ILC2-mediated tumor progression using animal models and human samples. This work builds directly on previous findings from the same group, showing that ILC2s in bladder cancer drive the recruitment of MDSCs into the tumor, thereby promoting an immunosuppressive microenvironment. Here the novelty relies on the mechanism triggering ILC2 effector function in bladder cancer which involves the secretion of NGF by mast cells that activates TrKa-expressing ILC2s, resulting in tumor growth and progression. Most experiments are well performed and appropriate. However, some key experiments are missing, preventing conclusions about the specificity and importance of the described mechanisms.

We thank the reviewer for their interest in our manuscript and for all the valuable insights. In response to the comments, we have conducted additional experiments to provide clearer answers to the questions raised.

Comments:

Figure 1. Survival analyses. Can the authors describe the rationale for categorizing NGF expression into four quartiles? Can the authors perform survival analyses based on median expression. Is there evidence/data to suggest that normal bladder tissue express low RNA levels of NGF that would support the use of a cutoff value that could be applied to the bladder cancer patient cohort. This threshold would correspond to the level of NGF expression found in healthy bladder tissue, perhaps supporting the categorization into four quartiles and explaining why only the first quartile performs better in terms of survival. In addition, does the level of NGF correlate with disease stage, tumor grade, or other clinical covariates?
<u>Answer to reviewer</u>: We apologize to the reviewer if the rationale for the survival analyses was not sufficiently clear. A survival analysis based on median expression confirms worse prognosis in patients with NGF expression levels higher than median, both in terms of overall and progression-free survival (**Figures 3A** and **3B here below**, respectively).

A **B**

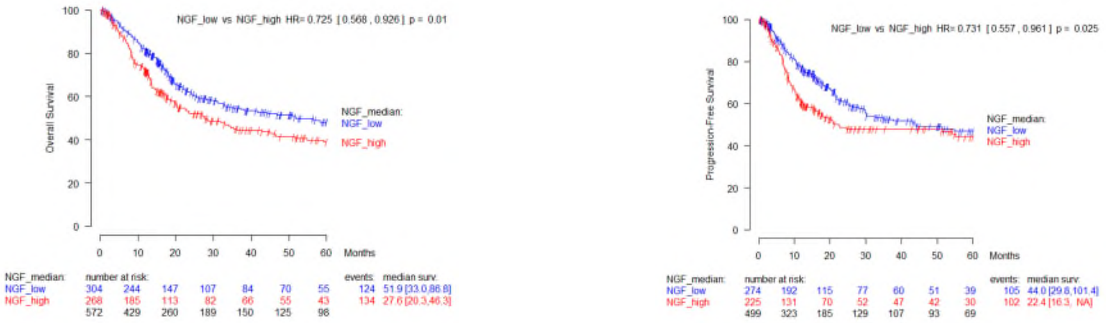

Figure 3. Overall survival (**A**) and progression-free survival (**B**) based on median NGF expression level.

We have separated patients into quartiles because this type of analysis may provide a more detailed view of the relationship between NGF expression varying across different quartiles and survival times. Indeed, as shown in our quantification of NGF in HD and patient bladder tissue (**Figure 1D** in our manuscript), NGF level is heterogeneous in BC patients, with some patients reaching extreme values. In addition, because NGF expression is a continuous variable, survival can be plotted as a continuous function of NGF expression level (**Figures 4A** and **3B here below,** for OS and PFS, respectively). Principles of the plotting method are similar to Figure 4 of prior work from Paik S. et al, N Engl J Med. 2004. We demonstrate that despite a

globally negative gradient in survival, there is a hyperbolic behavior among quartiles of expression level. Furthermore, the densest part of the plot corresponds to the second and third quartiles, and the extremes are inconsistent. A median-based categorization of NGF expression would hence obscure this heterogeneity and a quartile-based analysis is considered more informative.

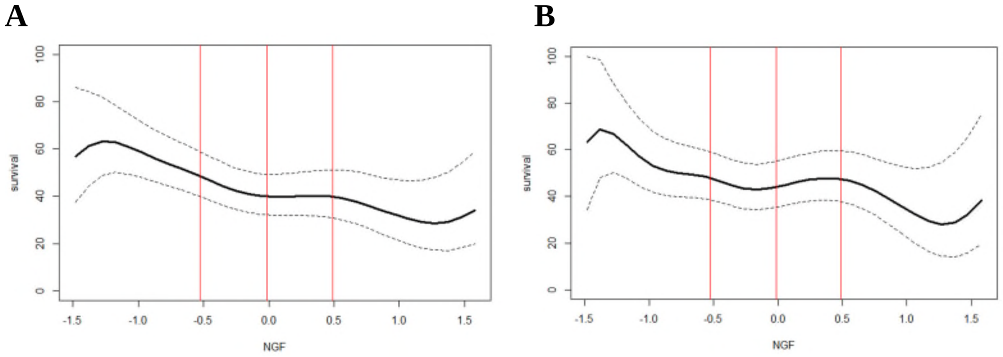

Figure 4. Overall survival (**A**) and progression-free survival (**B**) at 60 months as a continuous function of NGF expression level. The dashed curves mark the 95 percent confidence interval. Vertical red lines correspond, from left to right, to first quartile, median and third quartile, respectively.

The reviewer's suggestion of using a healthy NGF mRNA level as threshold to determine quartiles to separate BC patients and test survival is interesting. However, the BC patient datasets that we combined and used for survival analysis do not contain matched healthy bladder tissue. In addition, gene expression within these datasets was measured using different technologies (i.e., RNAseq or microarrays). We therefore cannot estimate which NGF mRNA level is considered as being "healthy". However, we provide comparative NGF levels measured as ng per protein between healthy and cancer bladder tissue in our manuscript in Figure 1D.

Finally, the reviewer asked whether NGF level differed among stages, grade or clinical data. We therefore provide visual illustration for NGF expression based on grade, T stage and age groups (**Figure 5 here below**). NGF expression does not differ among grade, T stage or age groups, and was therefore not shown in the manuscript.

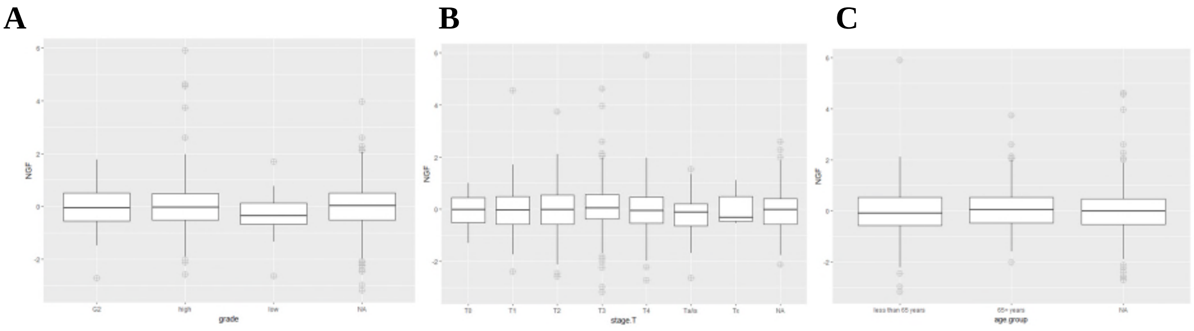

Figure 5. NGF expression level based on grade (**A**), T stage (**B**) and age (**C**) groups.

Figure S1. Based on qPCR analyses, the authors found that "human bladder cancer cell lines showed barely detectable transcripts of neurotrophic receptors" and, based on these data, concluded that "NGF might preferentially signal through TrKa+ ILC2s, rather than directly on tumor cells". However, the authors did not use the same housekeeping gene for normalization and therefore, it is complicated to compare the levels of expression found in ILC2s to tumor cell lines. To validate their observations, multiplex IHC staining should be performed using CD45, CD3, GATA3 and TRKA on tumor samples to estimate Trka expression on ILC2s and

other cell types in the tumor microenvironment. This multiplex IHC can be extended to NGF expression and TPSAB1 (figure 4) to show the colocations or interaction between Mast cell derived NGF expression and Trka expressing ILC2s.

Answer to reviewer: we fully agree with the reviewer. We are showing in the revised Supplementary Figure S3C gene expression data using the same housekeeping gene, by highlighting with a dashed line the mean gene expression level in ILC2s (extracted from graphs presented in revised Figure 1G, Supplementary Figure S1E).
To assess protein expression of TrkA directly in situ we have performed multiplex staining on human bladder tissue sections. We have co-stained for TrkA, CD45, GATA3 and CD3 on human bladder sections and show in the revised Figure 1I that TrkA is co-localizing with GATA3 but not with CD3, suggesting preferential expression of TrkA protein on ILC2s, as also observed by flow cytometry (revised Figure 1H). Furthermore, to investigate the relationship between NGF-expressing mast cells and ILC2s, we have performed co-staining for Tryptase (as surrogate marker for mast cells) and NGF. We observed co-localization of NGF with Tryptase^{+} cells in bladder tissue, as shown in the revised Figure 2D. By comparing the NGF content in mast cells in healthy versus tumor bearing bladder from patients, we observed significant enrichment of NGF in the disease setting (revised Figure 2D, right graph). Finally, to determine if ILC2s can sense mast-cell derived NGF, bladder tissue sections were co-stained for Tryptase, CD45, GATA3 and CD3. As illustrated in the revised Figure 2E, mast cells are located near ILC2s in the bladder tissues of patients, suggesting that NGF released by mast cells might be sensed by neighboring ILC2s.

Figure 2 and Figure S2. As mentioned by the authors in the discussion, TrKa expression on ILC2s is heterogenous. Did the authors observe a difference in cytokine production between TrKa positive and negative ILC2s?

Answer to reviewer: we thank the reviewer for raising this point on TrkA expression heterogeneity. To define if there is an intrinsic phenotypic and functional difference in TrkA^{+} vs TrkA^{-} ILC2s we performed mRNAsequencing on freshly sorted pure TrkA^{+} vs TrkA^{-} ILC2s from 3 healthy individuals. Our analyses showed that 45 genes are significantly upregulated in TrkA^{+} ILC2s while 27 genes in TrkA^{-} ILC2s (revised Supplementary Figure S2). TrkA^{+} ILC2s showed markedly higher expression of *KLRC1* (encoding for NKG2A), *KLRK1* (NKG2D) and *KLRD1* (CD94), surface receptors typically enriched in NK cells, as well as *TYROBP (KARAP/DAP12)*, which encodes a transmembrane signaling adaptor containing an immunoreceptor tyrosine-based activation motif (ITAM) in its cytoplasmic domain. TYROBP is known to associate with the killer-cell inhibitory receptors (KIRs) and mediate activating signal transduction, suggesting that TrkA^{+} ILC2s may be functionally modulated through NK-like pathways. In contrast, TrkA^{-} ILC2s exhibited property of pro-inflammation, indicated by increased expression of *IL22* and TNF superfamily members such as *CD70* and *TNFSF9* (encoding 41BB-L). This information has been added to the main manuscript at pages 6-7.

To experimentally assess the function of TrkA^{+} vs TrkA^{-} ILC2s, we short-term expanded ILC2s from 3 different healthy donors and at the end of the expansion we sorted TrkA^{+} and TrkA^{-} ILC2s. We cultured them for 48h in medium alone (RPMI 8% human serum, 20U/ml rhIL-2) or in medium supplemented with IL-33, a typical ILC2 activator (5 ng/ml). As illustrated in **Figure 6 here below**, TrkA^{+} ILC2s show a trend to produce less Type 2 cytokines at resting state, and less IL-13 in response to IL-33, in comparison to TrkA^{-} ILC2s. However, the reactivity of TrkA^{-} cells is very variable depending on the donor tested.

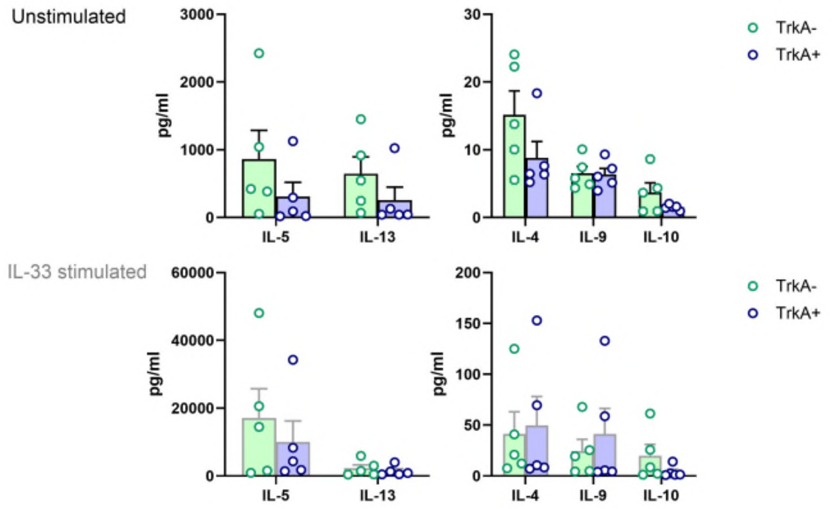

Figure 6. Type 2 cytokine secretion was measured in the supernatant of purified TrkA^{+} or TrkA^{-} ILC2s after 48h culture, in medium alone (unstimulated) or supplemented with IL-33.

Does use of GW441756 impair NGF-induced ILC2 transmigration and chemotaxis and reduce pS6, pERK and pCREB levels. Did the authors perform a dose response for NGF and TrKa inhibitor GW441756 (IC50 2nM and the authors used it at a concentration of 10uM).

Answer to reviewer: As Reviewer 1 also noted, some of our functional assays, such as transmigration, chemotaxis, and signaling, were conducted with a limited sample size. To address this, we repeated these experiments to increase the number of biological replicates and to further investigate if GW441756 exposure impairs NGF-mediated ILC2 functions. Our results confirmed that NGF consistently induces cytokine secretion and S6 phosphorylation in ILC2s, both of which are significantly reduced following pre-treatment with GW441756 (revised Figures 2B-C, Supplementary Figure S3E). In contrast, we found that GW441756 did not affect ILC2 transmigration or chemotaxis, suggesting that these processes are likely not mediated through NGF signaling via its high affinity receptor TrkA. Therefore, we have removed the transmigration and chemotaxis data from the manuscript. Similarly, since GW441756 selectively inhibited S6 phosphorylation, but not pERK and pCREB, we concluded that the latter may be downstream of NGF signaling via the low affinity receptor p75. Therefore, we have also removed the pERK and pCREB data. These revised findings support the conclusion that NGF activates ILC2s primarily via the mTOR pathway. This data is discussed in the revised manuscript at page 8.
Regarding the dose selection, for GW441756 we selected the highest concentration, that was not toxic on ILC2s in the MTT assay (revised Supplementary Figure S3D).

Did the authors confirm their findings using mouse ILC2s that would support the use of mouse models in subsequent experiments to study the role of NGF and ILC2s in bladder cancer progression. On a side note, error bars for multiple panels are missing.
Answer to reviewer: while we confirmed already in the original manuscript preferential expression of *Ntrk1* also in murine bladder ILC2s (revised Figure 3F), our aim was to validate the functional findings obtained with human ILC2s also using mouse ILC2s. However, while protocols to sort and expand murine lung ILC2s are well established, also in our hands, we found it more difficult when working with bladder infiltrating ILC2s. We successfully isolated murine lung ILC2s, but we found out that these cells were TrkA^{-}, hence unsuitable to assess TrkA-mediated functional readouts (revised Supplementary Figure S6C-D). To circumvent this

limitation, we sorted ILC2s from the mouse bladder and cultured them in the presence of different expansion cocktails and media (e.g., different concentrations of IL-2 and IL-7 in the presence or the absence of IL-33 or TSLP, using either RPMI or SFEM II media). Unfortunately, despite extensive efforts, we were unable to obtain enough cells to perform functional assays or transcriptomic analyses to delineate the profile of murine bladder $TrkA^+$ vs $TrkA^-$ ILC2s. Yet, the fact that freshly sorted ILC2s from murine bladders express high transcripts for TrkA (revised Figure 3F), that mouse tumor-bearing bladders contain higher levels of NGF than healthy ones (revised Figure 3D), and that mast cell express higher transcripts of NGF than other cell types (revised Figure 3H), recapitulating our findings in patients, shows a strong inter-species parallelism, attesting the validity of our *in vivo* model.

Figure 3. Is there a positive correlation between the level of NGF found in bladder and tumor burden (tumor weight)? This would further suggest a role for NGF in tumor progression.
Answer to reviewer: we thank the reviewer for raising this point. As suggested, we analyzed the correlation between NGF levels and tumor weight. As shown in the **Figure 7 here below**, we did not find a positive correlation suggesting that NGF levels in the bladder do not directly correlate with tumor burden in our model.

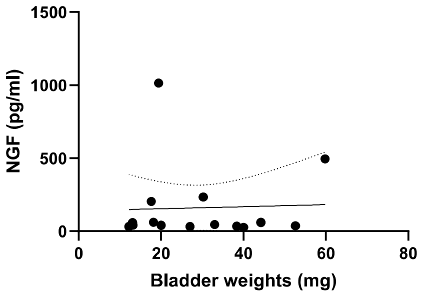

Figure 7. Correlations between NGF levels and bladder tumor weight.

Figures 4 and 5. A direct link between mast cell-derived NGF secretion and ILC2 function is missing. Given that TPSAB1 negative cells also express NGF (Figure 4F), a mast cell independent pathway might be involved. What is the identity of these cells? To demonstrate the specificity of this pathway, the authors should use a combination of in vitro culture systems (e.g co-culture of mast cells and ILC2s or the culture of ILC2s in mast cell conditioned media in the presence or not of TrKa inhibtor) and conditional mouse models (Ngf floxed animals [RMRC 13175] crossed to mast cell specific cre transgenic mice]. Furthermore, does the depletion of Tregs (e.g. Foxp3DTR model) or the ablation of mast cells (e.g. KO mouse models) phenocopy the outcomes observed in ILC2 deficient mice. In addition, the RORafl/flIL7Rcre mice display reduced ILC1s and dysfunctional ILC3s compared to littermate controls. Can the authors perform rescue experiments with WT or CRISPRKO Trka ILC2 in ILC2 deficient mice to ascertain of the specificity of the pathway as both ILC1s and ILC3s express low levels of Ntrk1 (Figure 3F).
Answer to reviewer: we agree with the reviewer concerning the missing link between mast cells-NGF and ILC2s, and the ILC2-downstream effect on Tregs. As suggested, to tackle these questions we have performed both *in vitro* and *in vivo* experiments.
For *in vitro* experiments, we have used the mast cell line HMC1 and showed that upon activation it produces NGF (revised Supplementary Figure S4B). Hence, we have co-cultured HMC1 cells with ILC2s, pre-treated or not with the TrkA inhibitor GW441756. As a readout, we quantified the cytokines in the supernatant after 48 hours (revised Figure 2F). No Type 2 cytokines were secreted by mast cells only. Instead, a strong increase in Type 2 cytokine secretion was observed when mast cells were co-cultured with ILC2s, an effect that was at least partially abrogated by the TrkA inhibitor. Altogether, these results suggest that NGF is secreted

by mast cells in quantities sufficient to induce in vitro ILC2 functional activation. We present this data in the manuscript at page 8.
To define the *in vivo* relevance of this finding, we performed an antibody-based depletion of mast cells, using the ACK2 anti-cKit antibody, previously reported by others for mast cell *in vivo* depletion experiments. We observed a significant survival advantage in mice treated with the anti-cKit antibody in comparison to the untreated animals, supporting the fact that mast cells have a protumoral function in our model (revised Figure 3I-J, page 9).

Regarding the ILC2-Treg axis, we obtained Foxp3DTR mice (DEREG mice). We intravesically instilled the MB49 bladder cell line in the animals and depleted Tregs by i.p. Diphtheria injections. We observed a significant survival advantage in Treg depleted mice, suggesting that Tregs support tumor growth in our model (revised Figure 4K-L, page 10).

Lastly, we agree with the reviewer that rescue experiments with WT or CRISPR based gene edited ILC2s to knock out TrkA would be very interesting. However, genetic deletion should be performed on bladder-infiltrating ILC2s, knowing that ILC2s isolated from other tissues, e.g., the lungs, do not express TrkA (Supplementary Figure S6C-D). Therefore, these cells, even WT, once transferred, might not express TrkA and might not migrate to the bladder, thus impairing a comparison of the TrkA gene function. CRISPR based editing of bladder ILC2s was not possible in our hands, since we were unable to expand purified bladder ILC2s in sufficient numbers, despite extensive efforts to culture them with different expansion cocktails. Therefore, we believe that, though very interesting, addressing this question would necessitate a dedicated experimental development outside the scope of this manuscript.

Figure S5. Can the authors indicate the absolute cell numbers for all the indicated populations.
Answer to reviewer: as also suggested by Reviewer 1, comment 1, we have performed a more comprehensive, longitudinal analyses of the tumor immune infiltrate in littermate and ILC2 KO animals. We have developed and validated a 28-marker panel for ex-vivo monitoring of immune cell subsets by spectral flow cytometry. These analyses have been performed at day 0 (prior tumor instillation) and at day 1 and 3 post-tumor instillation. We confirm the increase of MDSCs, Tregs and eosinophils, alongside with neutrophils, in tumor-bearing littermates as compared to ILC2KO mice (revised Figure 4B, Supplementary Figure 8). For T and NK cells, we did not observe major differences in terms of subset differentiation (**Figure 1 of this PBP reply, shown again here below)**, but we found a trend for heightened IFNγ^{+} CD8^{+} T cells and a significant increase of the CD8/Treg ratio and, a parameter that has been shown to highly correlate with strong anti-tumor immunity in many cancer types (revised Supplementary Figure 9, page 9-10).

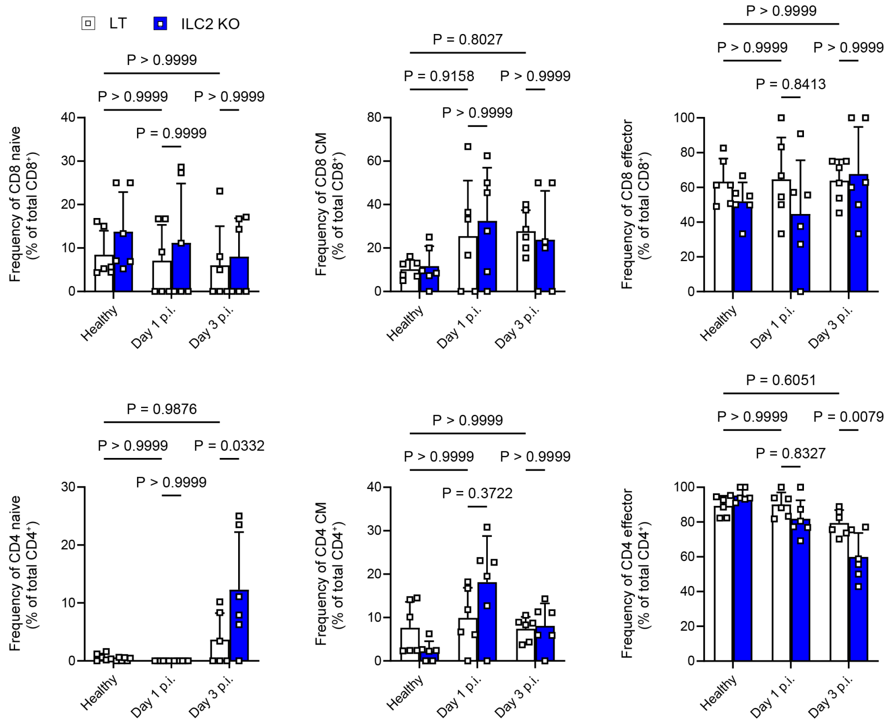

Figure 1. T cell subset distribution in tumor-bearing littermates (LT) and ILC2KO animals. Frequency of naïve, central memory (CM) and effector CD8 (upper graphs) and CD4 (lower graphs) T cells in healthy and tumor bearing RORα$^{fl/fl}$Il7r^{Cre-} (LT, white bars) and RORα$^{fl/fl}$Il7r^{Cre+} (ILC2 KO, blue bars) mice on day 1 (Day 1 p.i.) and day 3 (Day 3 p.i.) post-MB49 instillation, determined by flow cytometry.

Figure 6. It would be important to show that TrKa inhibition phenocopies the observations made in ILC2 deficient mice. Specifically, the authors should assess the frequency and number of Tregs in the tumor after treatment. Are improved survival outcomes associated with decreased ILC2 cytokine production? The data presented in Figure S6A are critical but, given the small sample size, no firm conclusions can be drawn. In addition, does intravesical delivery of NGF promote tumor growth, associated with increased production of ILC2-derived cytokines and accumulation of intratumoral Tregs?

Answer to reviewer: we thank the review for the interesting suggestions. We have performed immune phenotyping of WT animals treated or not with the TrkA inhibitor and quantified the tumor-infiltrating Treg in the 2 mouse groups as well as ex-vivo cytokine secretion. We observed a decrease in ILC2s, and more importantly IL-13^{+} ILC2s, alongside a significant reduction in Tregs upon treatment (Supplementary Figure S11B-C, page 11).

We agree with the reviewer that the intravesical instillation of NGF would be an interesting approach to recapitulate *in vivo* the ILC2 activation that we observed *in vitro*. However, not having this experimental procedure in our animal licenses, and not having found any publicly available publication providing pharmacokinetic evidence for NGF effects if delivered intravesically, we consider that this experiment would necessitate extensive set up that falls out of the scope of the manuscript. NGF instillation not being the ultimate therapeutic purpose for patients' treatment (rather its blockade) we leave this work for the continuation of these studies.

Discussion. Regarding the data presented in Figure S6C, the authors conclude that "strong survival reduction [are observed] in gastric and colorectal cancer patients with high NGF transcript levels and ILC2 infiltration". As there is no direct correlation between NGF levels, ILC2 infiltration and survival in these tumors, the authors either soften this statement or provide the data supporting this claim.

Answer to reviewer: we agree with the reviewers and have softened our statement (page 12).

Material and methods. The authors should indicate whether male or female mice were used in experiments as sex-differences have been reported on ILC2s. Also, in MMT assay, it is GW441756 and not GW5441756.
Answer to reviewer: we thank the reviewer for pointing to this aspect. Indeed, we performed the experiments in female mice, since the technical gesture of intravesical instillation is possible only in female animals.
As suggested, we have corrected the typo on GW441756.

Figure legends. The authors should indicate the exact sample size for each panel in the figure legends as well as the number of times that each reported experiment were repeated. All experiments should be repeated 2 or 3 times for data reliability.
Answer to reviewer: we thank the reviewer for the suggestion and have included this information in the Figure legends.

Lastly, a more balanced view or increased discussion of the pro and anti-tumorigenic functions of ILC2s would have been appropriate, as well as reference to original research articles rather than reviews.
Answer to reviewer: we fully agree with the reviewer and have provided a more balanced description of pro- vs anti-tumorigenic functions of ILC2s, by providing original articles rather than reviews as references.

Reviewer #3 (Remarks to the Author): with expertise in cancer immunology

The manuscript by Falquet et al investigates ILC2 functions in bladder cancer, using patient-derived specimen, in vitro experiments and mouse models, as well as inferring publicly available RNAseq data including large cohorts of patients. The main claim of the paper is that a mast cell-NGF-ILC2-Treg axis supports bladder cancer growth via mast cell derived NGF, which activates ILC2 to induce Treg, favoring tumor escape.
The story is interesting and holds translational value given by in vivo experiments with a TrkA inhibitor, also in association with anti PD-1. Yet, most of the results are correlative than mechanistic and conclusions are not fully supported by the data provided.

We would like to thank the reviewer for their positive feedback on our work and all the constructive comments. We have performed new experiments and modified the manuscript to address the questions, as detailed below.

1) It is not clear why the authors chose to study NGF, among all the many neuronal derived molecules that can potentially activate ILC2s. The rationale should be expanded in the introduction or at the beginning of the result section.
Answer to reviewer: we apologize with the reviewer if the rationale for the NGF choice was not well explained. In fact, we initially screened for traditional ILC2 activators (e.g., IL-33, TSLP, IL-25, PGD2) in the sera and urine of bladder cancer patients, as compared to healthy donors, but no difference was observed. Therefore, we searched for alternative triggers and by reviewing old and more recent literature we realized that neurotrophins have been associated with bladder disorders (as outline in the revised main manuscript at page 4). Next, by using the TCGA BLCA dataset we searched for correlations of neurotrophins with survival and observed that NGF transcripts were negatively correlating with survival in patients (see revised Figures 1A-B).

2) Fig 1. How many human samples were used for NGF quantification in human bladder tissues/urines from cancer patients and healthy donors? This should be specified in the figure legend
Answer to reviewer: we thank the reviewer for the suggestion, also raised by reviewer 2. We have included this information in all Figure legends.

3) Throughout the manuscript, please clearly indicate how many mice were used for in vivo/ex vivo experiments.
Answer to reviewer: this information has been included in all Figure legends.

4) Immunohistochemistry/immunofluorescence on tumor tissues (human and mouse) should be provided to show TrkA expression. Indeed, authors only consider ILC2s as possible sensors of NGF in the tumor microenvironment. Even if tumor cells are shown to have negligible levels of TrkA, several other stomal cells could express it.
Answer to reviewer: we agree with the reviewer that this information is very relevant. To assess protein expression of TrkA directly in situ we have performed multiplex staining on bladder tissue sections from HDs and patients. We have co-stained for TrkA, CD45, GATA3 and CD3 on bladder sections and show in the revised Figure 1I that TrkA is co-localizing with GATA3 but not with CD3, suggesting preferential expression of TrkA protein on ILC2s, as also observed by flow cytometry (revised Figure 1H).
Further, as indicated by the reviewer 1, it is well known that mast cells can express TrkA. To assess if bladder mast cells express TrkA, we have performed co-staining for Tryptase and TrkA

on human bladder tissue sections. As shown in **Figure 2 of this PBP reply (shown again here below)** we observed TrkA expression on mast cells. However, as outlined in the graph in **Figure 2**, the expression level of TrkA tend to be higher in cells other than mast cells.

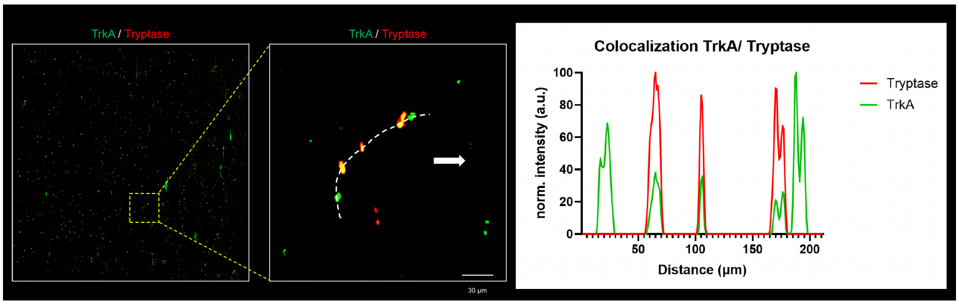

Figure 2. Tissue section was immunostained for mast cells (Tryptase, red) and TrkA (green) and imaged using confocal fluorescence microscopy. A selected region of interest within the merged imaged is indicated by a dashed square and corresponds to the magnified area shown in the adjacent image. Colocalization of TrkA and Tryptase is demonstrated by overlapping intensity profiles (TrkA: green line; tryptase: red line) measured along the segmented white dotted line shown in the upper panels of individual channels. Scale bar, 30 µm.

Furthermore, to investigate the relationship between NGF-expressing mast cells and ILC2s, we have performed co-staining for Tryptase (as surrogate marker for mast cells) and NGF. We observed co-localization of NGF with Tryptase^{+} cells in bladder tissue, as shown in the revised Figure 2D. By comparing the NGF content in mast cells in healthy versus tumor bearing bladder from patients, we observed significant enrichment of NGF in the disease setting (revised Figure 2D, right graph). Finally, to determine if ILC2s can sense mast-cell derived NGF, bladder tissue sections were co-stained for Tryptase, CD45, GATA3 and CD3. As illustrated in the revised Figure 2E, mast cells are located near ILC2s in the bladder tissues of patients, suggesting that NGF released by mast cells might be sensed by neighboring ILC2s.

5) Double immunofluorescence for Triptase and NFG is instead provided (Fig 4F). Yet, the quality of the staining is poor, with high background. The staining also shows NGF+ Tryptase- cells, therefore arguing for other sources of NGF rather than mast cells within the tumor. This issue should be expanded in the discussion.
Answer to reviewer: we thank the reviewer for this comment, that relates to the previous one. As mentioned above, to investigate the relationship between NGF-expressing mast cells and ILC2s, we have performed co-staining for Tryptase (as surrogate marker for mast cells) and NGF. We observed co-localization of NGF with Tryptase^{+} cells in bladder tissue, as shown in the revised Figure 2D. By comparing the NGF content in mast cells in healthy versus tumor bearing bladder from patients, we observed significant enrichment of NGF in the disease setting (revised Figure 2D, right graph). Finally, to determine if ILC2s can sense mast-cell derived NGF, bladder tissue sections were co-stained for Tryptase, CD45, GATA3 and CD3. As illustrated in the revised Figure 2E, mast cells are located near ILC2s in the bladder tissues of patients, suggesting that NGF released by mast cells might be sensed by neighboring ILC2s.

6) Fig 1F and S1B. Differently from what stated in the text, NTRK1 seems to be expressed by all the ILCs subsets and just overexpressed in ILC2s.
Answer to reviewer: we agree with the reviewers that our phrasing interpreting original Figure 1F and S1B is not correct. We have modified the text accordingly (page 6).

7) Fig. 1G. How many patients were used for this analysis?

Answer to reviewer: we have indicated in all Figure legends with information about sample size.

8) Fig. 1H. TrkA protein is not significantly higher in ILC2s from patients related to healthy donors. Does that mean that the NGF-TrkA axis is in place also in absence of the tumor? This seems at odds with survival data shown in Fig. 1. This is even more important considering that NGF stimulation induces cytokine production in ILC2s from bladder cancer patients and healthy donors as well (Fig. S2B).
Mast cell accumulation shown in tumors could count for the mechanism being enhanced in patients. This aspect should be addressed in the discussion.
Answer to reviewer: following the reviewer's comment, we stained more samples and assessed the expression of TrkA in peripheral ILC2s but also in tumor-infiltrating ILC2s in patients. As shown in the revised Figure 1H TrkA is upregulated in patients' ILC2s, suggesting that the NGF-ILC2 axis is preferentially active in bladder cancer patients. Nevertheless, we believe that also in healthy donors, ILC2s have the potential to respond NGF but in a milder way, as a consequence of lower NGF concentration (revised Figures 1D-E, 2D right graph) and a minor expression of the TrkA receptor (revised Figure 1H).

9) Fig S1I and S3E. Real time PCR for TrkA on tumor cells should include a ILC2 sample to facilitate comparison.
Answer to reviewer: we agree with the reviewer and have performed a new experiment to provide a complete Figure (revised Supplementary Figure S3C and S6E).

10) Fig2 D-F. Histograms show very tiny differences between control and NFG-stimulated conditions. To best evaluate the differences, data should be quantified using mean fluorescence intensity rather than percentage of positive cells. An FMO control (i.e. a sample not stained with the desired antibody) should also be included.
Answer to reviewer: we agree with the reviewer. We have performed additional experiments, using FMO as control and monitoring both MFI and % of positive cells (revised Figure 2C and Supplementary Figure S3I). We observed no major leakage of fluorophores on the minus color channels.

11) It is not clear why mitochondrial fitness was measured as redout of NGF stimulation in ILC2s (Fig. S2E).
Answer to reviewer: Given that previous studies have shown that ILC2s utilize both oxidative phosphorylation (OXPHOS) and glycolysis depending on their activation state, we decided to assess whether NGF treatment affects mitochondrial fitness. We have clarified this in the main text at page 8.

12) Fig 3C. The comparison should be between mast cells and tumor cells both sorted ex vivo from the bladder of the MB49 instilled mice. Indeed, microenvironment stimulation could enhance NGF production in tumor cells that are conversely negative during in vitro culture.
Answer to reviewer: we agree with the reviewer comment, also raised by Reviewer 1 (comment 2). To address this, we have sorted bladder-infiltrating mast cells and bladder cancer cells directly from tumor-bearing bladders and observed increase in NGF mRNA transcripts in mast cells as compared to tumor cells, further suggesting their involvement as NGF source in our setting (revised Figure 3H).
Second, as mentioned above, we agree with the reviewer that the quality of our in situ staining for NGF (shown in original Figure 4F) was not optimal. To strengthen our data we have performed co-staining for Tryptase (as surrogate marker for mast cells) and NGF. We observed

co-localization of NGF with Tryptase$^+$ cells in bladder tissue, as shown in the revised Figure 2D. By comparing the NGF content in mast cells in healthy versus tumor bearing bladder from patients, we observed significant enrichment of NGF in the disease setting (revised Figure 2D, right graph). Finally, to determine if ILC2s can sense mast-cell derived NGF, bladder tissue sections were co-stained for Tryptase, CD45, GATA3 and CD3. As illustrated in the revised Figure 2E, mast cells are located near ILC2s in the bladder tissues of patients, suggesting that NGF released by mast cells might be sensed by neighboring ILC2s.

13) Mast cell data seems most correlative than mechanistic. Authors should consider in vitro co-culture experiments between mast cells and ILC2s, also in presence of the TrkA inhibitor, to actually prove that MC can activate ILC2s via NGF production. Readout of such experiments should be cytokine production as shown in figure 2.
Answer to reviewer: we agree with the reviewer concerning the missing link between mast cells-NGF and ILC2s, and the ILC2-downstream effect on Tregs. As suggested, to tackle these questions we have performed both *in vitro* and *in vivo* experiments.
For *in vitro* experiments, we have used the mast cell line HMC1 and showed that upon activation it produces NGF (revised Supplementary Figure S4B). Hence, we have co-cultured HMC1 cells with ILC2s, pre-treated or not with the TrkA inhibitor GW441756. As a readout, we quantified the cytokines in the supernatant after 48 hours (revised Figure 2F). No Type 2 cytokines were secreted by mast cells only. Instead, a strong increase in Type 2 cytokine secretion was observed when mast cells were co-cultured with ILC2s, an effect that was at least partially abrogated by the TrkA inhibitor. Altogether, these results suggest that NGF is secreted by mast cells in quantities sufficient to induce in vitro ILC2 functional activation. We present this data in the manuscript at pages 8.
To define the *in vivo* relevance of this finding, we performed an antibody-based depletion of mast cells, using the ACK2 anti-cKit antibody, previously reported by others for mast cell *in vivo* depletion experiments. We observed a significant survival advantage in mice treated with the anti-cKit antibody in comparison to the untreated animals, supporting the fact that mast cells have a pro-tumoral function in our model (revised Figures 3I-J, page 9).

14) Data from ILC2KO mice are impressive (Fig.5). Yet, these results do not directly prove that the reduction of Treg is due to the absence of ILC2s. Indeed, ILC2KO mice have also reduced frequencies of MDSC and eosinophils. Authors cannot exclude a bystander effect on Tregs, given the well-known MDSC-Treg crosstalk and the fact that eosinophils can produce TGFβ, a master Treg-polarizing cytokine. This issue should be at least discussed.
Answer to reviewer: as suggested by the reviewer we have discussed this limitation in the updated manuscript (page 14).

15) The mast cell- Treg crosstalk should be discussed as well, in the frame of the existing literature.
Answer to reviewer: as suggested by the reviewer we have discussed this limitation in the updated manuscript (page 14).

16) A key issue is that ILC2s functions that count for Treg accumulation/activity are underexplored. Authors report an in vitro experiment (Fig. 5I) showing that ILC2s can induce Tregs from naïve CD4 T cells. Nevertheless, this experiment does not address the involvement of the NGF/TrkA axis in this phenomenon. Control conditions in which ILC2 are pretreated with NGF or pre-cultured with mast cells, in presence or absence of the TrkA inhibitor, should be added.

17) Also, this experiment does not explore the mechanisms by which ILC2s promote Treg conversion. Is this mediated by cytokine production? Treg conversion is usually induced, at least in vitro, by TGFβ and IL2, which however were not measured in NGF-treated ILC2s (experiments in Fig.2).
Also, authors suggest a contact-dependent mechanism (page 8 line 225), but this should be proven or excluded with transwell experiments, and the rationale of this conclusion is lacking.
Answer to reviewer for points 16 and 17: we thank the reviewer for these comments that we addressed performing additional experiments. We cultured ILC2s for 48h in medium only or in the presence of NGF, after pre-treatment of not with GW544175. After washing, we cocultured them with autologous naïve $CD3^+CD4^+CD45RA^+$ T cells for 6 days. Alternatively, we collected the SN of 48h-stimulated ILC2s to be used as conditioned medium on the naïve T cells. We show that ILC2s induce Tregs in a contact-dependent, NGF-independent manner (revised Figure 4G) Moreover, to assess the mechanisms by which Treg were induced, we monitored Ki67 expression and excluded that the observed Treg increase was due to proliferation (revised Figure 4H). To determine which molecules (surface molecules or soluble factors) could be involved in Treg induction upon ILC2 contact, we compared the phenotype of T cells cultured alone, with ILC2s or with their CM. As shown in the revised Figure 4I, among the markers known to be involved in Treg induction/accumulation, the markers preferentially upregulated upon contact with ILC2s were ICOS and OX40, suggesting that these molecules are involved in the Treg induction, as previously shown.
Nevertheless, the role of OX40-OX40L and ICOS-ICOSL in Treg induction does not exclude the possibility that also cytokines, produced following the contact between ILC2s and naïve T cells, could be responsible of sustaining Treg induction. Therefore, we evaluated the concentration of several cytokines, IL-2 included, in the 3 different conditions. As shown in the revised Figure 4J and Supplementary Figure 10D cytokines highly upregulated following the contact between ILC2s and naïve T cells were IL-5 and IL-13, while no changes were observed for IL-2 and IL-10. Interestingly, it was previously demonstrated that Tregs can be induced via IL-5 and IL-13.
Overall, in our setting, ILC2s induced Tregs irrespective of NGF presence, in a contact dependent manner most likely involving ICOS-ICOSL and OX40-OX40L with the contribution of IL-5 and IL-13. We have discussed these findings at page 10.

18) Figure 6. Which are the frequencies of tumor infiltrating Tregs in mice treated with the TrkA inhibitor? This is a key information to be provided to add mechanistic cues to the author's hypothesis.
Answer to reviewer: we agree with the reviewer for this interesting question, raised also by Reviewer 2. We have performed immune phenotyping of WT animals treated or not with the TrkA inhibitor, and quantified the tumor-infiltrating Treg in the 2 mouse groups. We observed a decrease in ILC2s, and more importantly in $IL\text{-}13^+$ ILC2s, alongside a significant reduction in Tregs upon treatment (Supplementary Figure S11B-C, page 11).

19) As BCG is the treatment choice for bladder cancer patients, did the authors consider treating tumor bearing mice with the combination of TrkA inhibitor and BCG?
Answer to reviewer: we fully agree with the reviewer, particularly on a translational perspective. A combination of TrkA inhibitor and BCG instillation should be tested, by carefully optimizing dosing and treatment schedule. Given that we don't have animal licenses in our Institution allowing the intravesical instillation of BCG, we are planning to set up this experiment in a follow up work, in collaboration with experts in the field (e.g., Prof M Ingersoll, Paris).

Reviewer #4 (Remarks to the Author): with expertise in bladder cancer, immunology, ILC2

This paper holds significant relevance in the field of ILCs in bladder cancer. Given the limited existing research in this area, the manuscript builds upon the foundation laid by a previous paper on the modulation of the T cell/MDSC ratio by ILC2s in bladder cancer. The study explores the novel NGF-TrkA-ILC2s axis's role in the development and progression of bladder cancer. It highlights the potential of TrkA blockade as an innovative therapeutic approach for bladder cancer patients, especially those resistant to immune checkpoint therapy, and the potential of the combination of anti-PD1 and GW441756. The study effectively combines human and mouse models and will be a relevant addition to the intratumoral ILC field in bladder cancer.

We appreciate the reviewer's positive evaluation of our work, and we have addressed their questions, as detailed below.

Fig. 1 - Include the number of healthy donors and cancer patients in figure legends, may also be a good idea to include the identifying markers for human ILCs in the legend since there is so much confusion about the gating strategy. (also in the S1D legend need details about the identification strategy for ILC1, 2, ILC3 clearly).
How long were the ILC2s cultured for short-term expansion?
Answer to reviewer: we thank the reviewer for pointing these aspects. We have clarified these methodological details in the updated version of the manuscript and provided all numbers of samples used in the updated Figure legends. We outlined the details of the gating strategy for ILCs in the Figure legend of Supplementary Figure S1D.

Fig 5C – will be useful to include a regular graph showing the difference in bladder tumor weights instead of just a picture.
Answer to reviewer: since we did not consistently weight the bladder tumors in our experiments, and only had some representative pictures available, we have decided to remove the illustrative picture.

While day 1 and day 3 are important to understand the immune landscape of the early tumor microenvironment, immune analysis at a more traditional sacrifice endpoint may yield critical information on the NGF-TrkA-ILC2s axis.
Answer to reviewer: as also suggested by reviewer 2 and reviewer 3, point 18), we have performed immunomonitoring on day +10 post-intravesical instillation in untreated versus TrkA inhibitor treated animals. We observed a decrease in ILC2s, and more importantly IL-13^{+} ILC2s, alongside a significant reduction in Tregs upon treatment (Supplementary Figure S11B-C, page 11).
We have also quantified Tregs and the CD8/Tregs ratio (the 2 parameters significantly different at earlier timepoints, when comparing tumor-bearing LT and ILC2KO animals) at a close to sacrifice endpoint (i.e., on day 10). In line with the observations on days 1 and 3, we observed a reduction in Treg numbers and a trend for an increase in the CD8/Tregs ratio in the animals lacking ILC2s (**Figure 8 here below**).

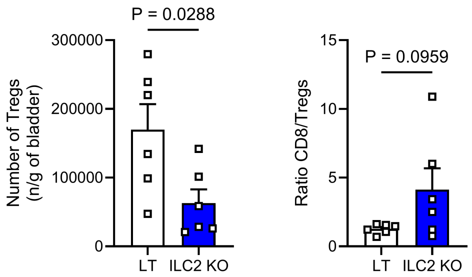

Figure 8. Tregs and CD8/Tregs ratio in tumor-bearing littermates (LT, white bars) and ILC2KO animals (blue bars) on day 10 post-MB49 instillation, determined by flow cytometry.

If possible, Single-cell spatial techniques will be extremely helpful in delineating the interactions of ILC2s with other immune cells (including Tregs) and stromal cells and will give a broader picture of the interaction of ILC2s with the immune landscape.
Answer to reviewer: we fully agree with the reviewer. To assess spatial interactions directly in situ we have performed multiplex staining on human bladder cancer sections. We have co-stained for TrkA, CD45, GATA3 and CD3 on tumor-infiltrated bladder sections and show in the revised Figure 1I that TrkA is co-localizing with GATA3 but not with CD3, suggesting preferential expression of TrkA protein on ILC2s, as also observed by flow cytometry (revised Figure 1H). Furthermore, to investigate the relationship between NGF-expressing mast cells and ILC2s, we have performed co-staining for Tryptase (as surrogate marker for mast cells) and NGF. We observed co-localization of NGF with Tryptase^{+} cells in bladder tissue, as shown in the revised Figure 2D. By comparing the NGF content in mast cells in healthy versus tumor bearing bladder from patients, we observed significant enrichment of NGF in the disease setting (revised Figure 2D, right graph). Finally, to determine if ILC2s can sense mast-cell derived NGF, bladder tissue sections were co-stained for Tryptase, CD45, GATA3 and CD3. As illustrated in the revised Figure 2E, mast cells are located near ILC2s in the bladder tissues of patients, suggesting that NGF released by mast cells might be sensed by neighboring ILC2s.

To confirm that ILC2s are working through Tregs, Tregs need to be depleted or Treg KO mice need to be used. Since there are other immune cells involved, this will investigate the extent to which Tergs contribute to ILC2-mediated effects.
Answer to reviewer: we thank the reviewer for raising this point. To address this aspect, we obtained Foxp3DTR mice. We intraperitoneally injected Diphtheria toxin to deplete Tregs in FoxP3DTR mice, and intravesically instilled the MB49 bladder cell line in WT and FoxP3DTR animals. We observed a significant survival advantage in mice Treg depleted, suggesting that Tregs support tumor growth in our model (revised Figure 4K-L, page 10).

Overall, the paper has exciting findings that will take the field forward and is also translationally relevant in the case of BCG nonresponsive tumors.
Answer to reviewer: We thank the reviewer for his/her enthusiasm in our work and for considering the translational relevance of our observations.

REVIEWER COMMENTS

Reviewer #2 (Remarks to the Author):

I thank the authors for their revised version of the manuscript. The authors responded satisfactorily to all my comments. The quality of the manuscript has been significantly improved, and the conclusions are supported by the experimental data. I only have three minor comments.
We would like to thank the reviewer for their appreciation of our efforts to improve the quality of our manuscript. We addressed the three remaining minor comments, as detailed below.

• Line 528 – "Additional markers used for Treg immune phenotyping used include…" – the second "used" should be deleted.
Answer to reviewer: the second "used" has been deleted.

• Line 635 – There is a typo – it is microcentrifuge
Answer to reviewer: the typo has been corrected.

• Before publication, the authors should ensure that the Bulk RNA seq dataset has been deposited in the NCBI Gene Expression Omnibus
Answer to reviewer: as suggested, the RNAseq data of human ILC2s have been deposited in NCBI's Gene Expression Omnibus and are accessible through GEO accession number GSE311046 (https://www.ncbi.nlm.nih.gov/geo/query/acc.cgi?acc=GSE311046). This information has been included in the main text, lines 501-503.

Reviewer #3 (Remarks to the Author):

The manuscript has been substantially improved with the addition of numerous new experiments. Many of these address my initial requests. Indeed, the relevance of mast cells (MC) as the main source of NGF, which in turn stimulates/activates TrkA+ ILC2, in this tumor setting has been proved. However, some of the new results raise further questions, as they appear to conflict with the authors' original hypotheses.
We would like to thank the reviewer for their positive feedback on our revised work. We addressed the remaining points, as detailed below.

1. The new in vitro experiment shown in Figure 4G clearly demonstrates that ILC2 induce the conversion of naive T cells into Tregs in vitro, through a mechanism that is independent of NGF. If this is true, it suggests that the MC-NGF-ILC2- axis and the ILC2-Treg axis operate independently from each other. Nevertheless, both are relevant to sustain tumor growth as demonstrated by new experiments in mice depleted of either MC (treated with anti cKIT antibody) or Treg (DEREG mice).
Answer to reviewer: we agree with the reviewer that the 2 axes act independently, NGF being necessary for ILC2 activation, but dispensable for Treg induction.

2. In this new scenario, the MC-derived NGF axis activates ILC2 to produce type 2 cytokines with pro-tumoral functions (the authors previously showed that ILC2-derived IL-13 rewires an immunosuppressive TME towards bladder cancer growth, ref.6 of the manuscript), but these cytokines are not required for Treg conversion. Indeed, while the authors show that T cell-ILC2 contact induces IL-5 and IL-13 production (Figure 4J), these same cytokines are also produced by ILC2 following HMC1 stimulation and are reduced if the NGF receptor is blocked (Figure 2F). Yet, stimulation with NGF or blockade of its receptor does not alter the ability of ILC2 to convert Tregs (Figure 4G).
Answer to reviewer: we fully agree with the reviewer that NGF-induced IL-5/IL-13 production is insufficient to drive ILC2-mediated Treg induction, as shown in the original **Figure 4G**, right panel.

3. Yet it is undoubted from data in figure 4 (experiments in ILC2 KO mice) that the absence of ILC2 impairs the accumulation/differentiation of Treg. The most likely relevant mechanism for Treg induction by ILC2 therefore remains stimulation via OX40 and ICOS. The authors should show that ILC2 express OX40L and ICOSL, and whether these molecules are modulated (probably not) by NGF stimulation or co-culture with MC.
Answer to reviewer: As suggested, we quantified the expression of OX40L and ICOSL on ILC2s, either unstimulated, after 48-hour stimulation with NGF or upon 48-hour co-culture with the HMC1 cell line. We observed upregulation of both OX40L and ICOSL on ILC2s after exposure to HMC1 cell line (new **Supplementary Figure S10D**). In contrast, treatment with NGF did not alter either OX40L or ICOSL expression on ILC2s, as shown in the **Figure 1 here below**.
The main text has been edited to include these observations, lines 262-264.

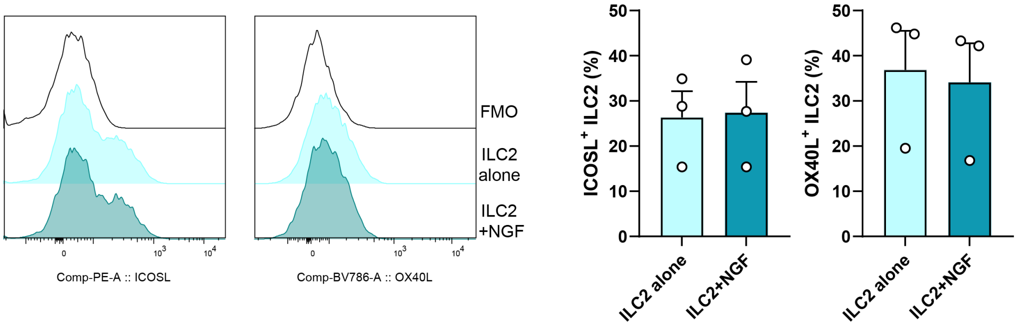

Figure 1. Representative histograms (left) and quantification (right) of ICOSL and OX40L expression by human short-term *in vitro* expanded ILC2s unstimulated (ILC2 alone) or stimulated with NGF (ILC2+NGF) for 48 hours (n=3).

An in vitro experiment using blocking antibodies against OX40 and ICOS, as well as IL-5 and IL-13 blockers, should definitively clarify the mechanism.
Answer to reviewer: To define by which mechanisms ILC2s ultimately convert CD4 T cells into Tregs, we co-cultured ILC2s with autologous naïve T cells, in the presence of various blocking antibodies. While the addition of anti-ICOSL, anti-OX40 and anti-IL-5 did not affect Treg induction, anti-IL-13 antibody significantly impaired Treg induction. These data suggest that upon interaction with naïve T cells, ILC2s secrete IL-13 that drives Treg differentiation.
These results have been added as Supplementary **Figures S10E and S10G** and discussed in the main text at lines 264-267, 270-274 and 394-395.

4. A possible interpretation linking MC-derived NGF to Tregs might instead arise from the data shown in Figure S11 B-C. Mice treated with GW441756 exhibit fewer ILC2 and also fewer Tregs. It is possible that NGF has a chemoattractive effect on ILC2. This data were present in the original manuscript (in vitro experiments in Figure 2C) but were later removed at revision stage for unclear reasons. If this is the case, the new mechanistic hypothesis could be that the MC-NGF axis serves to recruit ILC2 to the tumor, which then mediate Treg conversion via OX40L-OX40 and ICOSL-ICOS interactions.
Answer to reviewer: We agree with the reviewer that NGF might act as chemoattractant. However, the initial *in vitro* data presented in the original manuscript could not be reproduced due to high variability among donors. This is the reason for removing them from the resubmitted manuscript.

These points need to be clarified experimentally; otherwise, some of the conclusions in the manuscript and in the abstract should be revised and toned down.
Answer to reviewer: we believe that with the additional results we have clarified the remaining reviewer's concerns.

Finally, as a minor point, I suggest to rephrase text and legend od figure S11 B-C to clarify that GW441756 was given in wild type (not ILC2 KO) tumor bearing mice.
Answer to reviewer: as suggested, this information has been corrected both in the text and in the Figure legend.
